# Lipid dynamics and cardiovascular risk profiles on carbohydrate- or saturated fat-based diets: secondary analysis of the CARBFUNC trial

**DOI:** 10.1016/j.ebiom.2026.106524

**Published:** 2026-10-24

**Authors:** Cathrine Loddervik, Johnny Laupsa-Borge, Cathrine H. Sommersten, Kristin Amundsen, Arve Ulvik, Kristine K. Johannessen, Synnøve Halvorsen, Terje H. Larsen, Ottar K. Nygård, Jutta Dierkes, Gunnar Mellgren, Simon N. Dankel

**Affiliations:** a Hormone Laboratory, Department of Medical Biochemistry and Pharmacology, Haukeland University Hospital, Bergen, Norway; b Department of Clinical Science, University of Bergen, Norway; c Department of Clinical Medicine, Centre for Nutrition, University of Bergen, Norway; d Department of Ageing, Health and Care, City of Bergen, Norway; e Bevital AS, Bergen, Norway; f Department of Heart Disease, Haukeland University Hospital, Bergen, Norway; g Department of Biomedicine, University of Bergen, Norway

**Keywords:** Low-carbohydrate high-fat diet, Lipoprotein metabolism, Lipoprotein subfractions, Triglyceride-rich lipoproteins, Cardiometabolic risk, Obesity

## Abstract

**Background:**

Obesity involves complex changes in energy metabolism and increased cardiovascular disease (CVD) risk independently of low-density lipoprotein cholesterol (LDL-C). Diets high or low in carbohydrates or fats can be effective for reversing obesity, but their distinct effects on metabolic health and detailed lipid profiles may differentially impact overall CVD risk, particularly with large differences in saturated fatty acid (SFA) intake.

**Methods:**

In a 2-year randomised controlled trial, 193 males and females with obesity were assigned to 1) an acellular carbohydrate-based diet (A-HCLF, comparator arm), 2) a cellular carbohydrate-based diet (C-HCLF), or 3) a low-carbohydrate high-fat (LCHF) diet (75 energy percent fat, thereof 30 SFAs). As secondary outcomes of the trial, plasma lipids and lipoprotein particle subfractions were quantified by nuclear magnetic resonance spectroscopy assay, and coronary artery calcium score by CT imaging. Data were analysed by constrained linear mixed-effects modelling.

**Findings:**

Attrition was 74 (39%), 114 (59%), 128 (67%), 135 (70%), and 162 (84%) at 3, 6, 9, 12 and 24 months, respectively. Self-reported adherence ranged from 63 to 85% up to 12 months and 40–60% for the last 12 months. During the first 9–12 months, the LCHF group showed significantly greater reductions than the A-HCLF comparator and C-HCLF diets in very low-density lipoprotein particles (VLDLs), triacylglycerols (TAGs), remnant cholesterol, and the TAG/high-density lipoprotein cholesterol (HDL-C) ratio, alongside increases in HDL-C, total apolipoprotein A1 (apoA1), apoA1 in large HDL particles, LDL-C (attributed to large LDL particles), and the LDL-C/apolipoprotein B ratio. At 24 months, there were still greater reductions on the LCHF compared to the A-HCLF diet for TAGs, VLDL particles, remnant cholesterol, and the TAG/HDL-C ratio, as well as a greater increase in large LDL particles.

**Interpretation:**

In adults with obesity, an LCHF diet rich in SFA markedly lowered TAG-rich remnants and raised HDL-C and apoA1 compared to higher-carbohydrate diets, while shifting LDL toward larger particles without significantly increasing apoB or small LDLs—a pattern that may indicate greater lipid turnover.

**Funding:**

This work was supported by the Trond Mohn Research Foundation, Bergen (Norway), the 10.13039/501100005036University of Bergen (Norway), Kostfonden, Stockholm (Sweden), and the 10.13039/501100013263Norwegian Health Association.

**Research in context**

**Evidence before this study**

We searched PubMed, Embase, and reference lists of relevant articles for studies published from database inception to August 2026, without language restrictions, using terms related to “low-carbohydrate diets,” “saturated fat,” “lipoproteins,” “lipid subfractions,” and “cardiovascular risk.” We included randomised controlled trials, cohort studies, and meta-analyses examining lipoproteins and cardiovascular disease (CVD) risk, as well as dietary macronutrient composition and blood lipids in adults, particularly those with overweight or obesity, and excluded short-term feeding studies (<4 weeks) and studies without detailed lipid measurements. Previous dietary intervention studies have shown that carbohydrate restriction can alter conventional lipid markers, including LDL-cholesterol (LDL-C), its apolipoprotein apoB, and triacylglycerols (TAGs), all of which are associated with elevated risk of CVD. LDL-C and apoB are expected to rise with higher intake of saturated fatty acids (SFAs), and TAGs to fall with lower carbohydrate intake. However, most studies of low-carbohydrate diets did not include very high intakes of SFA, leaving a knowledge gap despite strong recommendations to limit SFA intake, and the relative impact of carbohydrate quality and amount is also unclear. Existing evidence further indicates that dietary links to CVD risk extend beyond LDL-C, being influenced by other lipid features such as TAG-rich lipoproteins, remnant cholesterol, HDL composition, and LDL particle characteristics. The overall quality of this evidence is moderate, with heterogeneity across studies and few longer-term randomised controlled dietary intervention trials comprehensively characterising lipoprotein subclasses alongside other CVD risk factors, including blood pressure, insulin-glucose homoeostasis, and coronary artery calcium (CAC) scores.

**Added value of this study**

This secondary analysis of the 2-year CARBFUNC randomised controlled trial provides a detailed characterisation of lipid and lipoprotein responses in males and females with obesity assigned to diets differing markedly in carbohydrate quality, amount, and saturated fat intake. Relative to baseline, a low-carbohydrate high-fat (LCHF) diet very high in SFA was associated with lower circulating TAG-rich remnants and higher HDL cholesterol and apolipoprotein A1. LDL-C concentrations, involving larger LDL particles, increased to some extent, with no significant increases in apoB or small LDL particles. These changes differed significantly from higher-carbohydrate diets based on either more processed/flour-based (“acellular”) or whole-food-based (“cellular”) carbohydrate sources, between which no consistent differences were observed. HOMA2-IR improved to a similar extent across all diet groups, while CAC scores showed no group-level worsening over 12 months.

**Implications of all the available evidence**

The study provides insight into the complex metabolic adaptations that occur when switching from a standard high-carbohydrate diet to an LCHF diet very high in SFA, including lowered TAG and remnant cholesterol without an increase in apoB. Our findings indicate that in people with obesity, an LCHF diet, even when very high in SFA, was associated with favourable changes in several lipid features commonly linked to insulin resistance and cardiovascular risk, particularly TAGs and remnant lipoproteins, while shifting LDL particles toward a larger size without increasing particle number. This pattern may reflect more efficient lipid handling, and warrants further investigation into the health implications of diet- and SFA-dependent increases in LDL-C in the context of broader lipid and metabolic profiles. Future research should focus on long-term clinical endpoints, more diverse populations, and further mechanistic investigation of how dietary patterns influence lipid metabolism and vascular health.

## Introduction

The persistent global rise in obesity heightens concerns over the associated health risk and growing burden of obesity-related diseases. Among these, cardiovascular disease (CVD) is particularly prominent, accounting for 41% of obesity-related deaths.^1^ For decades, circulating lipids, such as low-density lipoprotein cholesterol (LDL-C), have been the cornerstone target in mitigating CVD risk, either through pharmacological treatment or dietary modification. The cholesterol-centric paradigm has fundamentally shaped dietary recommendations, emphasising a reduction in the intake of saturated fatty acids (SFAs) in favour of polyunsaturated fatty acids (PUFAs) with the aim to lower LDL-C and consequently CVD risk.^2^, ^3^ However, alongside the widespread increase in obesity, the interplay between risk factors, such as insulin resistance, diabetes, and inflammation, involves complex interactions that contribute to overall risk. Also, a systematic review of the evidence indicates that people with obesity generally do not show the same diet-induced increase in LDL-C as leaner individuals,^4^ and that there is substantial CVD risk also with well-controlled LDL-C.^5^, ^6^

The obesity-associated CVD risk has been linked to a cluster of metabolic disturbances and blood lipid concentrations associated with insulin resistance syndrome (also known as metabolic syndrome), including the “lipid triad” of elevated levels of triacylglycerol (TAG)-rich lipoproteins and their remnant particles, low high-density lipoprotein cholesterol (HDL-C), and qualitative changes of LDL particles, particularly increased small LDL particles.^6^, ^7^, ^8^, ^9^ These are all characteristics that reflect and may even influence atherogenic processes, beyond LDL-C levels.^6^ Moreover, it is important to recognise that LDL-C levels also fluctuate with various physiological states, including dietary changes in carbohydrates independent of SFAs,^10^ periods of fasting and starvation,^11^ and with infections, sepsis,^12^, ^13^ and other causes of immune activation.^14^ Therefore, appreciating the more complex physiological changes in lipid and energy metabolism, beyond circulating LDL-C as a risk indicator, is necessary to accurately understand the potential of diet to impact CVD risk.

The physiological regulation of lipoproteins involves a finely tuned, dynamic equilibrium, where they are efficiently produced, transported, and utilised to meet the body's cellular demands under various physiological and nutritional circumstances. In this process, the apolipoprotein B (apoB) in LDL particles serves to direct the lipid cargo of the particles to peripheral tissues expressing the LDL receptor. It has been proposed that proper function of this mechanism of lipid turnover may be more important for CVD risk than the concentration of apoB or LDL-C per se.^10^ Notably, recent studies have found that lower ratios of LDL-C/apoB^15^ and non-HDL-C/apoB^16^ correlate with increased risk for future cardiac events, while higher ratios are inversely correlated. These ratios essentially reflect LDL particle size and the efficiency of lipid turnover processes, which may be adversely affected by insulin resistance—an independent CVD risk factor and a common feature of obesity.^17^

Understanding how diet influences these broader changes in lipid profiles is critically important for addressing the rising metabolic disturbances and CVD risk associated with obesity. Yet, there remains a lack of longer-term randomised controlled trials (RCTs) that evaluate the effect of diet on more extensive metabolic and CVD-risk patterns, particularly diets with very high SFA content and with simultaneous assessment of clinical/functional parameters such as insulin-glucose homeostasis and coronary artery calcium (CAC) score. Here, we present multimarker changes in circulating lipid-/, lipoprotein profiles and other CVD risk factors in a 2-year RCT (Dietary Carbohydrate and Internal Body Fat, CARBFUNC), comparing three nutritious, isocaloric diets with different amounts and types of carbohydrates, including a low-carbohydrate high-fat (LCHF) diet with 30% of the energy from SFA.

## Methods

### Study design and participants

This paper includes secondary analyses from the two-year RCT CARBFUNC^18^ that investigated differential effects of three dietary patterns differing in carbohydrate quantity or quality and SFA content on visceral and hepatic adiposity in males and females with obesity. Recruitment took place from 28 August 2017 to 20 May 2019, and study visits were conducted between January 2018 and March 2021. The study participants were adults (aged 20–55 years) with obesity (body mass index ≥30 kg/m^2^ and/or waist circumference [WC] >88 cm for females and >102 cm for males), who had maintained a stable body weight over the past two months prior to the inclusion. Sex was self-reported by participants at baseline. Excluded individuals were those who had undergone any medical treatment (surgical or antibiotic) within the past two months, pregnant or lactating females, postmenopausal females, smokers, or individuals with excessive alcohol consumption (>2 units per day). Additionally, individuals with inflammatory bowel disease, allergies, or those taking any medication affecting lipid metabolism, anti-diabetic drugs, or other medications related to serious illness were excluded.

All eligible participants were randomised to one of three study diets using block randomisation (R package *blockrand* v1.5, RStudio, Inc., Boston, MA, USA) with block sizes ranging from 6 to 9 and stratification by sex. Due to the intervention design, participants and staff were not blinded after randomisation. The statistician remained blinded to group assignments until all analyses presented here were completed. Fasting plasma samples and anthropometric measures were collected at study visits at baseline, and at 3, 6, 9, 12 and 24 months.

### Study interventions

The three-armed dietary intervention was designed to be normo- and iso-caloric (2000 kcal for females, 2500 kcal for males), with similar amount of protein (17 energy percentage [E%]) and PUFAs (7–10 E%), and low in added sugar (<5 E% for the “acellular” and <1 E% for the “cellular” and LCHF diets). There were no pre-specified targets for monounsaturated fatty acids (MUFA). All three diets were designed to meet nutritional requirements for essential nutrients such as amino acids, fatty acids, vitamins, and minerals by encouraging daily servings of fruits and vegetables, fish consumption 2–3 times per week and 2–3 portions of dairy per day. Both carbohydrate-based diets were designed to be broadly consistent with current dietary recommendations with respect to macronutrient profile, added sugar intake, and wholegrain and fibre-rich sources of digestible carbohydrates. With similar profiles for these standard carbohydrate quality metrics, the carbohydrate-based diets were designed to primarily distinguish the degree of cellularity, where the acellular carbohydrate sources encompassed flour-based products such as bread, pasta, and bakery products, primarily with at least 50% wholegrain content, whereas the cellular carbohydrate sources had preserved cellular structure, such as intact legumes, whole grains (i.e., not flour-based), and unpolished rice. Additional information regarding the study diets is published elsewhere.^18^

The three diets were as follows: 1) acellular carbohydrate-based (A-HCLF) diet (45 E% carbohydrate, 38 E% fat, 10–12 E% SFA), 2) cellular carbohydrate-based (C-HCLF) diet (45 E% carbohydrate, 38 E% fat, 10–12 E% SFA), and 3) a low-carbohydrate high-fat (LCHF) diet (8 E% carbohydrate, 75 E% fat, 30 E% SFA).

To assess dietary adherence, the participants were asked to record their food intake through an online dietary recording system (www.diet.no; operated by Dietika AS) every second week for three days (two weekdays and one weekend day). Physical activity levels were recorded in the same system, as previously described.^18^ Every third month, participants also rated their own adherence to the prescribed diet through a questionnaire, using a rating scale ranging from 0% (indicating no adherence) to 100% (indicating complete adherence), with 20% increments. Additionally, as an objective estimate of carbohydrate restriction, ketone bodies were measured from the plasma samples collected at the study visits.

### Anthropometry, blood pressure, insulin resistance, and ketones

Body weight, height, WC, and blood pressure were assessed at each study visit. Body weight was measured using a Class III approved calibrated scale (Seca 877, Seca, Hamburg, Germany). WC was measured thrice at the point between the lower rib and the iliac crest using a non-elastic tape, recording the average of the last two measurements. Height was measured while standing upright with a portable stadiometer (Seca 217, Seca, Hamburg, Germany). Blood pressure was measured three times in a rested, seated position using a digital blood pressure monitor (Omron HEM-970XL, Omron Healthcare), reporting the mean of the last two measures. Fasting serum glucose and insulin C-peptide were measured at the Department of Medical Biochemistry and Pharmacology and at the Hormone Laboratory, Haukeland University Hospital according to standardised procedures. The homeostatic model assessment (HOMA) serves as an index of insulin resistance. We utilised the HOMA2 calculator developed by the University of Oxford (http://www.dtu.ox.ac.uk/homacalculator/index.php) to estimate insulin resistance (HOMA2-IR). Total ketones were calculated as the sum of β-hydroxybutyrate and acetoacetate, measured in fasting plasma samples by gas chromatography-tandem mass spectrometry at BEVITAL AS, Bergen (https://bevital.no), as previously described.^19^

### Lipid and lipoprotein analyses

Particle concentrations and content of lipids and apolipoproteins of lipoprotein main fractions and subfractions were measured from fasting plasma samples at the MR Core Facility, Norwegian University of Science and Technology (Trondheim, Norway), using nuclear magnetic resonance (NMR) spectroscopy assay. For some participants or timepoints, plasma samples were unavailable or of insufficient volume for these analyses. The samples were analysed in 5 mm NMR tubes in a Bruker Avance Neo 600 MHz spectrometer (Bruker BioSpin GmbH, Rheinstetten, Germany), equipped with a 5 mm BBI probe. Concentrations of lipoproteins and associated lipids and apolipoproteins were calculated from the measured amplitudes of their spectroscopically distinct lipid methyl group NMR signals.

Based on the described method, the following parameters were measured or calculated: total apoB-containing lipoproteins, lipoprotein main fractions (total very low-density lipoprotein particles [VLDLs], intermediate-density lipoprotein particles [IDLs], and low-density lipoprotein particles [LDLs]), LDL subfractions (LDL-1, LDL-2, LDL-3, LDL-4, LDL-5, and LDL-6), total lipids (TAGs, total cholesterol [TC], free cholesterol [FC], LDL-C, HDL-C, and phospholipids [PLs]), total apolipoproteins (apoB, apoA1, and apoA2), lipids (TAGs, TC, FC, and PLs) in lipoprotein main fractions (VLDLs, IDLs, LDLs, and high-density lipoprotein particles [HDLs]), apolipoproteins in lipoprotein main fractions (apoB in VLDLs, IDLs, and LDLs; apoA1 and apoA2 in HDLs), and lipids (TAGs, TC, FC, and PLs) in VLDL subfractions (VLDL-1, VLDL-2, VLDL-3, VLDL-4, and VLDL-5), in LDL subfractions (LDL-1 to LDL-6), and in HDL subfractions (HDL-1, HDL-2, HDL-3, and HDL-4), as well as apoB in LDL subfractions and apoA1 and apoA2 in HDL subfractions. Measurement of TC within VLDLs obtained from the NMR analysis was used as an estimate for remnant cholesterol (remnant-C). Non-HDL-C was calculated by subtracting HDL-C from TC.

The concentrations of total apoB-containing lipoprotein particles (VLDLs, IDLs, LDLs, and LDL subfractions) were calculated from their respective apoB concentrations, considering that each VLDL, IDL, and LDL particle carries only one apoB molecule.^15^ The lipoprotein subfraction analysis was performed using the Bruker's IVDr Lipoprotein Subclass Analysis™ (B.I.-LISA™) platform, which applies a partial least-squares regression model to predict the concentrations of the reported parameters.^20^ The lipoprotein fractions were classified based on the following densities (in kg/L): VLDL (0.950–1.006), IDL (1.006–1.019), LDL (1.019–1.063), HDL (1.063–1.210), LDL-1 (1.019–1.031), LDL-2 (1.031–1.034), LDL-3 (1.034–1.037), LDL-4 (1.037–1.040), LDL-5 (1.040–1.044), LDL-6 (1.044–1.063), HDL-1 (1.063–1.100), HDL-2 (1.100–1.112), HDL-3 (1.112–1.125), and HDL-4 (1.125–1.210).^21^ We grouped LDL-1 and LDL-2 into large LDLs, LDL-3 and LDL-4 into medium LDLs, LDL-5 and LDL-6 into small LDLs, HDL-1 and HDL-2 into large HDLs, and HDL-3 and HDL-4 into small HDLs.

### Coronary artery calcium score

CAC score was assessed at baseline and at 6 and 12 months using non-contrast scans obtained with a Siemens dual source 256 Slice Somatom FORCE (www.siemens-healthineers.com). ECG-triggered scans of the heart region were analysed by an experienced reader using dedicated analysis software syngo.via (www.siemens-healthineers.com), to measure area-density (Agatston score^22^) in Hounsfield units (HU) and to calculate the total CAC score of all coronary arteries.

### Ethics

The study was approved by the Regional Ethics Committee in Western Norway (2017/621/REC West) and conducted in accordance with the Declaration of Helsinki (2024 version). All participants provided written informed consent. The trial record was first submitted to ClinicalTrials.gov on 23 August 2017, before enrolment of the first participant on 3 January 2018 (NCT03401970). The initial registry entry specified measures of glucose, insulin, lipids, and apolipoproteins, as well as CAC, as secondary outcomes. The uploaded protocol (v2.0, November 2025) is a retrospective update of the original protocol. No adverse events were recorded.

### Statistics

As the present analyses were secondary to the prespecified primary endpoint of the original CARBFUNC trial (change in visceral adipose tissue volume), no separate sample size calculation was performed for the outcomes reported here. The reported outcomes are between-group differences for relative and absolute change scores in blood levels of NMR-measured lipoprotein subclass concentrations and sizes. The analyses followed the intention-to-treat (ITT) principle and included all available outcome measurements. Data are presented as raw unadjusted means (standard deviation [SD]), geometric means (1 SD ranges), or mean score differences (±absolute/relative effect estimates [95% confidence intervals]). All inferential tests were two-tailed with a nominal alpha level of 0.05. The statistical analyses were conducted with R v4.5.2 (https://www.r-project.org), using the *tidyverse* packages (https://tidyverse.tidyverse.org) for data transformation and exploration, and plots were made using the *ggplot2* package v3.4.1.

Continuous study outcomes were analysed by baseline-adjusted constrained linear mixed effects models (cLMMs), a constrained longitudinal data analysis technique,^23^, ^24^, ^25^ with subjects as the random factor by using the lme function in the nlme package v3.1–168. We followed a design-driven approach with pre-defined fixed effects (‘time’, ‘sex’, ‘age’, ‘rac’, and ‘group × time’), random effects structure (random intercepts and slope for ‘time’), and covariance structure (first-order autoregressive correlation matrix). Sex was used as a stratum in the randomisation to ensure balanced representation of males and females and was, therefore, included in the model to give valid inference. Additionally, the model included the stratification variable ‘rac’, used to indicate whether participants were randomised as a couple or not. In case of heterogeneity, the model included a variance function allowing for different variances per stratum of ‘group’ and/or ‘time’. By excluding the main term ‘group’ and thereby constraining the baseline values to be equal across diet arms, a reasonable assumption in RCTs, the cLMM inherently adjusts for baseline differences.^23^ To obtain both baseline-adjusted changes within each group and differences in change scores between groups, we replaced the ‘group × time’ interaction term in the cLMM with a new variable that represents the treatment effect over time. This was achieved by adding a new column to the dataset in long format and setting this covariate equal to the ‘time’ variable for one diet group and to zero (representing baseline) at all time points for the other (comparator) group. This was done for each treatment arm, so that the intercept from the model output was equal regardless of the chosen comparator group. In planned comparisons showing absolute or relative within- and between-group differences, the categorical main terms (factors) in the models were encoded by user-defined simple contrasts where we applied the indicator type to code the constant term (intercept; e.g., 1, 0, 0, 0) and the other parameters (coefficient contrasts; e.g., −1, 1, 0, 0). To obtain the final coding scheme for the design matrix, the *base::t* function first transposed the initial contrast coefficient matrix, and then we used the *MASS::ginv* function to get the generalised inverse of the matrix before it was attached to the factor by the *stats::contrasts* function. In the between-group comparisons, the A-HCLF intervention was defined as the comparator group.

Values were transformed by the natural logarithm before analyses of responses in relative terms. Relative within-group changes from baseline to follow-up and between-group differences in change scores are reported in the text and tables as percentages calculated from the regression coefficients (i.e., the average of log-ratios) by formula 100 × (exp^estimate^ −1). However, in the figures we show results in relative terms as sympercents (s%), which are additive and symmetric percentage differences on the 100 log_e_ scale.^26^ This relative measure is calculated as the difference between the natural logs of two numbers multiplied by 100, i.e., 100 × ln(a) – 100 × ln(b), making it straightforward to present without back transformation. Linear mixed-effects modelling has been shown to efficiently deal with data sets containing missing outcome values and to be the optimal estimator in trials with repeated outcome measures comprising a large portion of missing data.^27^ Therefore, we did not pre-specify another strategy for dealing with potential intermittent missing repeated outcome measurements or missing data resulting from dropout, such as multiple imputation before mixed modelling, as this has shown to add no obvious gain compared to a standard mixed model approach without imputed values.^27^

We conducted additional exploratory analyses adjusting for diet adherence and sensitivity analyses using inverse probability weighting (IPW) to address potential bias due to dropout. The diet adherence–adjusted models repeated the primary cLMMs with additional adjustment for self-reported diet adherence and deviations between planned and reported intake derived from repeated dietary records. Self-reported adherence was assessed at each study visit on a 0–100% scale (in 20% increments). Dietary deviations were defined as the difference between planned and reported intake for total energy (kcal) and macronutrients (expressed as percentage of total energy intake), with baseline deviations set to zero. In the IPW analyses, observations with available follow-up outcome data were weighted by the inverse of the estimated probability of having observed follow-up data. Stabilised weights were estimated using logistic regression models, with baseline covariates (group, sex, RAC, and baseline outcome value) included in the numerator and both baseline covariates and time (visit) included in the denominator. The stabilised weights were incorporated into the cLMMs via the weights argument in the lme function using a fixed variance structure (varFixed(∼sw)), thereby incorporating the stabilised weights into the estimation. The IPW analyses were conducted using the *ipw* package v1.3.0. Both the exploratory diet adherence-adjusted analyses and the IPW sensitivity analyses were compared with the primary ITT analyses to evaluate whether estimated between-group differences and nominal significance status were materially altered. Although not part of the prespecified pairwise comparisons, we also performed a post hoc exploratory comparison between the C-HCLF and LCHF groups for key lipid outcomes using the same modelling framework as the primary ITT and diet-adherence adjusted analyses.

As the present analyses were secondary to the primary outcome of visceral adipose tissue volume,^18^ the results should be considered exploratory. Accordingly, adjustments for multiplicity were not performed, given the exploratory intent, and because a general adjustment method for mixed modelling of repeated measurements has not yet been developed due to difficulties associated with the correlation structure, which has to be taken into account.^28^ The R code used for the cLMM contrast coding and IPW sensitivity analyses can be made available from the corresponding author upon request.

### Role of funders

This work was supported by the Trond Mohn Research Foundation, Bergen (Norway), the University of Bergen (Norway), Kostfonden, Stockholm (Sweden), and the Norwegian Health Association. The funders had no role in the study design, data collection, data analysis, data interpretation, or writing of the manuscript.

## Results

### Study participants

Of the 193 participants randomly assigned to one of the three study diets, one participant withdrew consent, leaving 192 participants in the study after randomisation, of whom 30 completed the 24-month study period (Fig. 1). The study participants included in these analyses were middle aged males and females (age (mean ± SD): 41.7 ± 8.7 years) with abdominal obesity (body mass index: 36.6 ± 4.7 kg/m^2^; WC: 117 ± 12 cm). Baseline characteristics by diet groups are shown in Table 1. Baseline characteristics from participants who completed 12- and 24-month follow-ups are presented in Supplementary Table S9.

**Fig. 1:**
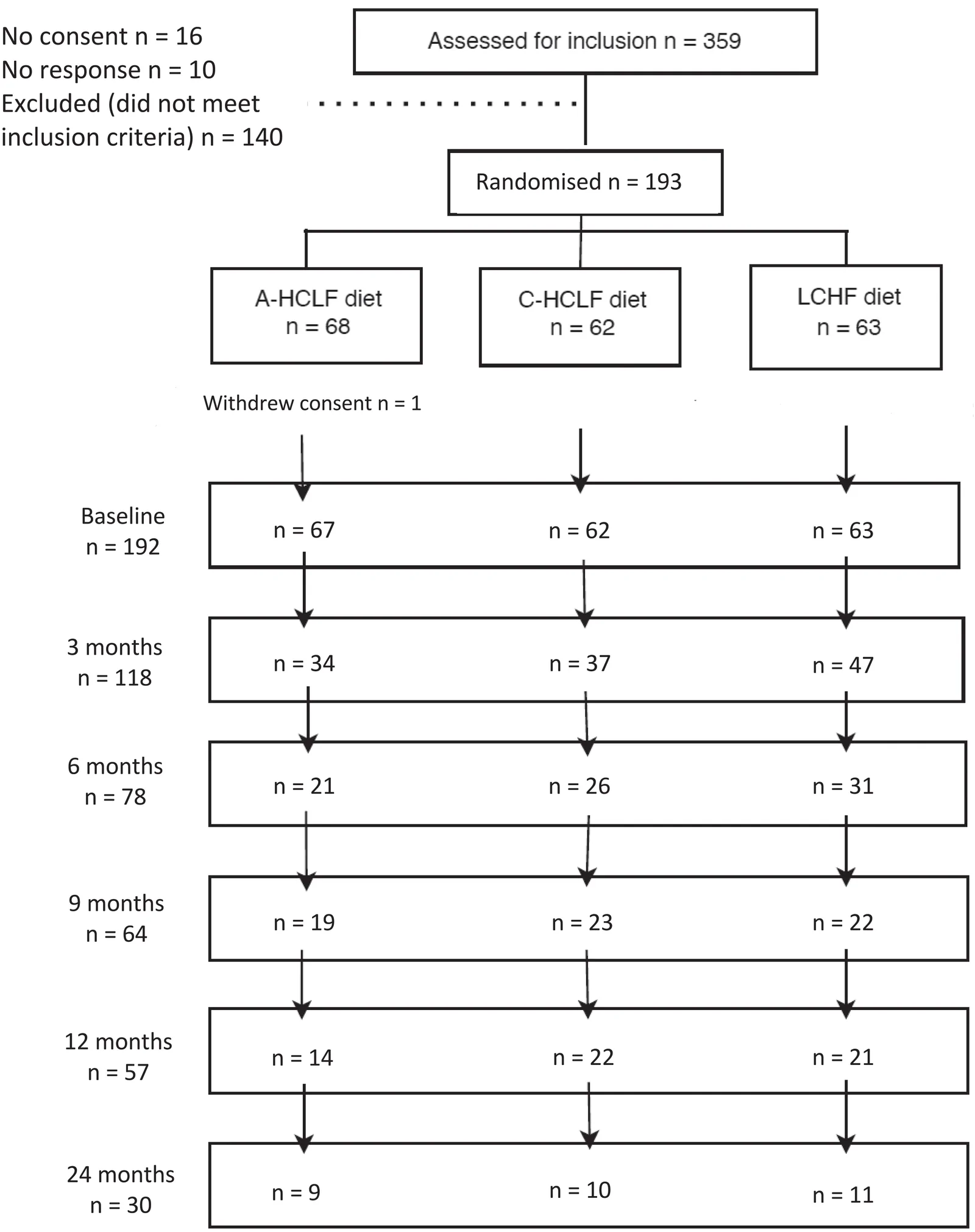
Flow diagram of the number of screened, excluded, included, and randomised participants. Ten participants did not respond to further contact after the baseline assessment and were therefore not randomised. Following the randomisation, one participant in the A-HCLF group withdrew consent. The decrease in number of participants at each subsequent timepoint was attributed to participant dropouts. Abbreviations: A-HCLF, acellular high-carbohydrate low-fat; C-HCLF, cellular high-carbohydrate low-fat; LCHF, low-carbohydrate high-fat.

**Table 1:** Baseline characteristics by diet groups.

Characteristics	A-HCLF (n = 67)	LCHF (n = 63)	C-HCLF (n = 62)
Age^a^	41.4 (8.8)	41.2 (8.8)	42.6 (8.7)
Sex			
Female, n (%)	32 (51)	32 (53)	32 (53)
Male, n (%)	31 (49)	28 (47)	29 (47)
Race/ethnicity			
Caucasian, n (%)	62 (98)	59 (98)	59 (97)
Non-Caucasian, n (%)^b^	1 (2)	1 (2)	2 (3)
Weight (kg)^a^	111 (20)	111 (20)	114 (18)
Body mass index (kg/m^2^)^a^	36.4 (4.3)	35.9 (4.8)	37.6 (5.2)
Waist circumference (cm)^a^	116 (12)	115 (12)	120 (13)
TAG (mmol/L)^c^	1.37 (0.85, 2.19)	1.25 (0.80, 1.98)	1.26 (0.85, 1.86)
TC (mmol/L)^c^	5.04 (4.20, 6.04)	5.14 (4.29, 6.15)	5.25 (4.48, 6.16)
LDL-C (mmol/L)^c^	2.95 (2.26, 3.84)	3.10 (2.41, 3.99)	3.23 (2.57, 4.06)
HDL-C (mmol/L)^c^	1.19 (0.97, 1.46)	1.22 (1.01, 1.47)	1.26 (1.05, 1.51)
Remnant-C (mmol/L)^c^	0.48 (0.27, 0.87)	0.44 (0.25, 0.76)	0.41 (0.23, 0.74)
Total LDLs (nmol/L)^c^	1305 (996, 1709)	1361 (1045, 1774)	1408 (1121, 1769)
Large LDLs (nmol/L)^c^	343 (256, 458)	364 (282, 470)	381 (283, 514)
Medium LDLs (nmol/L)^c^	367 (253, 532)	406 (296, 555)	422 (316, 565)
Small LDLs (nmol/L)^c^	572 (379, 865)	568 (370, 872)	589 (427, 812)
ApoB (g/L)^c^	0.86 (0.67, 1.10)	0.88 (0.69, 1.13)	0.91 (0.74, 1.12)
ApoA1 (g/L)^c^	1.31 (1.16, 1.48)	1.31 (1.16, 1.48)	1.33 (1.16, 1.52)
Glucose (mmol/L)^d^	5.35 (4.78, 5.98)	5.29 (4.71, 5.95)	5.25 (4.66, 5.92)
HbA1c (mmol/mol)^d^	34.0 (30.9, 37.3)	33.9 (30.5, 37.6)	35.1 (31.4, 39.1)
Insulin C-peptide (nmol/L)^d^	0.88 (0.60, 1.30)	0.80 (0.55, 1.16)	0.86 (0.59, 1.25)
HOMA2-IR (index)^d^	1.98 (1.32, 2.97)	1.79 (1.21, 2.63)	1.92 (1.30, 2.85)

Abbreviations: A-HCLF, acellular high-carbohydrate low-fat; apoA1, apolipoprotein A1; apoB, apolipoprotein B; C-HCLF, cellular high-carbohydrate low-fat; HbA1c, haemoglobin A1c; HDL-C, high-density lipoprotein cholesterol; HOMA2-IR, Homeostatic Model Assessment of Insulin Resistance 2; LCHF, low-carbohydrate high-fat; LDL-C, low-density lipoprotein cholesterol; LDLs, low-density lipoprotein particles; Remnant-C, remnant cholesterol; TAGs, triacylglycerols; TC, total cholesterol.

a Data are presented as mean (SD).

b Non-Caucasian ethnic backgrounds included Asian (n = 1 in the A-HCLF group; n = 1 in the LCHF group) and Somali and African (n = 2 in the C-HCLF group).

c NMR-derived lipid and lipoprotein variables are presented as geometric means (1 SD ranges) for participants with available baseline plasma data (A-HCLF, n = 61; LCHF, n = 53; C-HCLF, n = 60). Arithmetic means (SDs) of these variables are shown in Supplementary Table S1.

d Glucose, HbA1c, insulin C-peptide, and HOMA2-IR are presented as geometric means (1 SD ranges). Arithmetic means (SDs) of these variables are shown in Supplementary Table S1.

### Diet adherence and physical activity levels

Median self-reported diet adherence ranged from 63 to 85% up to 12 months and 40–60% by 24 months (Supplementary Table S2). Dietary records (available up to 12 months) have been described previously,^18^ indicating minimal changes in total energy and protein intake from baseline with no significant group differences. Intake of carbohydrates and total fat was similar and unchanged from baseline on the two higher-carbohydrate diets (41–43 E% carbohydrates, 36–38 E% fat), while carbohydrate intake was reduced to 10–15 E% on the LCHF diet. On the higher-carbohydrate diets, intake of SFA (11–13 E%), MUFA (12–14 E%) and PUFA (5–6 E%) also remained similar to baseline, while on the LCHF diet there was a sustained increase in intake of SFA (30–31 E%) and MUFA (22–23 E%), but not PUFA which showed more variation (Fig. 2). The calculated average physical activity level was similar in all three diet groups (1.5–1.6, i.e., “low active”), as reported previously.^18^

**Fig. 2:**
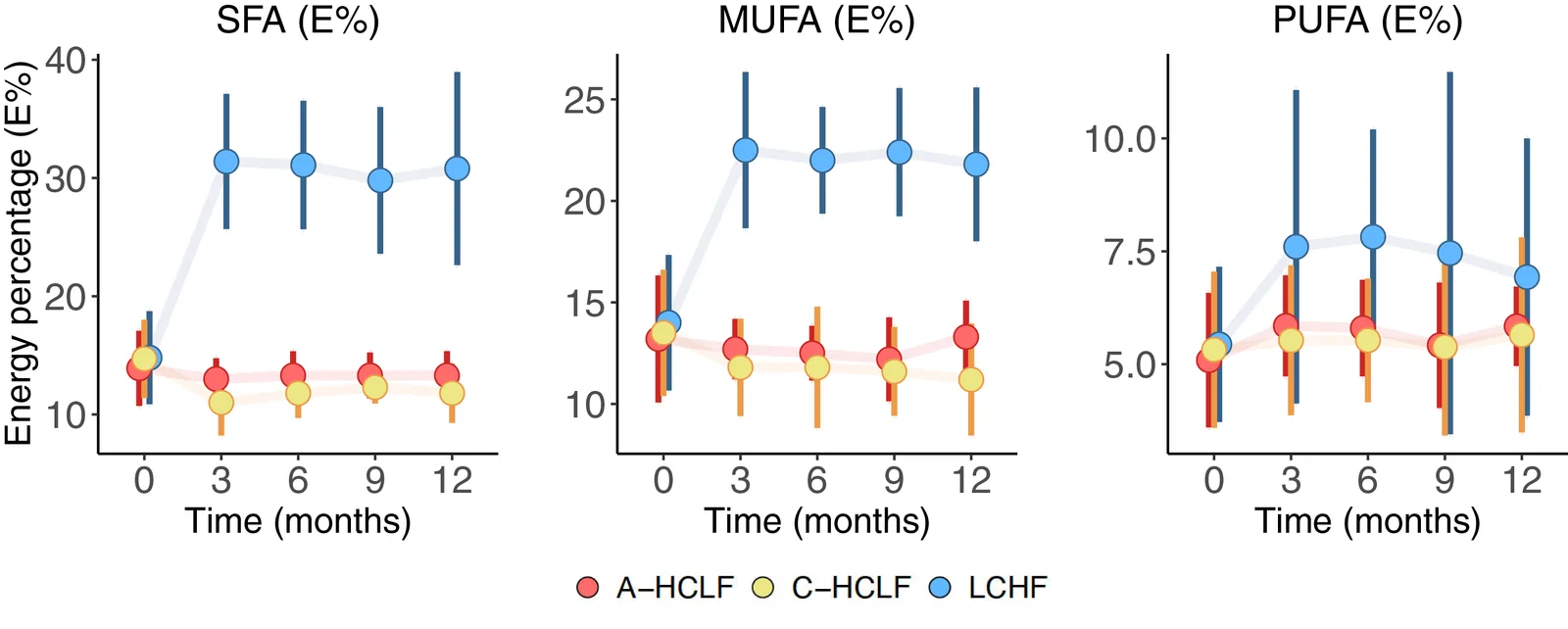
Dietary intake of SFA, MUFA, and PUFA at baseline and follow-ups. Values are presented as mean (SD) E% for each diet group. Intakes are estimated from three-day dietary records every second week during the intervention. The numbers of participants with available dietary records at baseline and between baseline and 3, 6, 9 and 12 months were 67, 53, 23, 19 and 12 in the A-HCLF group; 62, 53, 29, 23 and 21 in the C-HCLF group; and 63, 57, 30, 21 and 19 in the LCHF group, respectively. Because dietary records were collected throughout the follow-up periods, participants contributed dietary records even if they did not attend the corresponding follow-up visit, in line with the intention-to-treat principle. Abbreviations: A-HCLF, acellular high-carbohydrate low-fat; C-HCLF, cellular high-carbohydrate low-fat; E%, energy percentage; LCHF, low-carbohydrate high-fat; MUFA, monounsaturated fatty acid; PUFA, polyunsaturated fatty acid; SFA, saturated fatty acid.

Consistent with the planned reduction in carbohydrates, the LCHF group demonstrated a significantly greater increase in total ketones compared to the A-HCLF group from baseline to 3, 6 and 9 months (LCHF vs. A-HCLF at 9 months: +74.9% [+77 μmol/L] vs. −23.2% [−39 μmol/L], p < 0.001). Within groups, total ketones increased significantly up to 9 months in the LCHF group, and up to 3 months in the A-HCLF and C-HCLF groups (Supplementary Figure S1, Supplementary Table S3).

### Anthropometry, blood pressure and insulin resistance

No significant between-group differences in change scores for body weight were observed at any time point. Within groups, all three diet groups showed comparable and significant reductions in body weight of 5–7% over the course of 12 months. By 24 months, a significant reduction in body weight was sustained only in the A-HCLF group. For WC, the LCHF group exhibited significantly greater reductions at 6 and 9 months compared with the A-HCLF group. Significant reductions in WC were seen within all three diet groups at each time point over the 24-month period, amounting to reductions of 5–6% (Supplementary Figure S2, Supplementary Table S3).

For systolic blood pressure, a significant between-group difference in change scores was observed between the LCHF group and the A-HCLF group only from baseline to 9 months (LCHF vs. A-HCLF at 9 months: −2.63% [−3.78 mmHg] vs. +2.34% [+2.87 mmHg], p = 0.033). No significant between-group differences were observed in change scores for diastolic blood pressure at any time point, although a significant reduction was observed within the LCHF group up to 9 months, and within the C-HCLF group at 3 months (Supplementary Figure S2, Supplementary Table S3). HOMA2-IR, an estimate of insulin resistance, showed no significant between-group differences in any of the group comparisons at any time point. However, significant reductions in HOMA2-IR were demonstrated within all three diet groups up to 12 months (Supplementary Figure S1, Supplementary Table S3).

### Standard lipids

To assess the overall impact of the diets on lipid metabolism and lipid profiles, we first examined standard lipid measures. We observed a significant between-group difference in relative change scores from baseline to 6 and 9 months between the LCHF group and the A-HCLF comparator group for TC (LCHF vs. A-HCLF at 9 months: +5.03% [+0.24 mmol/L] vs. −5.62% [−0.26 mmol/L], p = 0.001), and LDL-C (+12.8% [+0.36 mmol/L] vs. −8.51% [−0.21 mmol/L], p < 0.001), and for HDL-C from baseline to 3, 6, 9 and 12 months (LCHF vs. A-HCLF at 12 months: +13.5% [+0.17 mmol/L] vs. +5.61% [+0.077 mmol/L], p = 0.009) (Fig. 3, Supplementary Table S4). Within the LCHF group, there was a significant increase in TC only at 9 months, in LDL-C at 3, 9, 12 and 24 months, and in HDL-C throughout the whole 24-month intervention period. In the C-HCLF group, TC was significantly reduced up to 12 months, whereas LDL-C was significantly reduced only at 3 and 6 months. In the A-HCLF group, TC was significantly reduced up to 9 months, while LDL-C was significantly reduced at 6 and 9 months. Both carbohydrate groups also showed a significant decrease in HDL-C at 3 months, followed by a slight, significant increase at 9 and 12 months in the A-HCLF group and a greater, significant increase in the C-HCLF group at 24 months (Fig. 3, Supplementary Table S4).

**Fig. 3:**
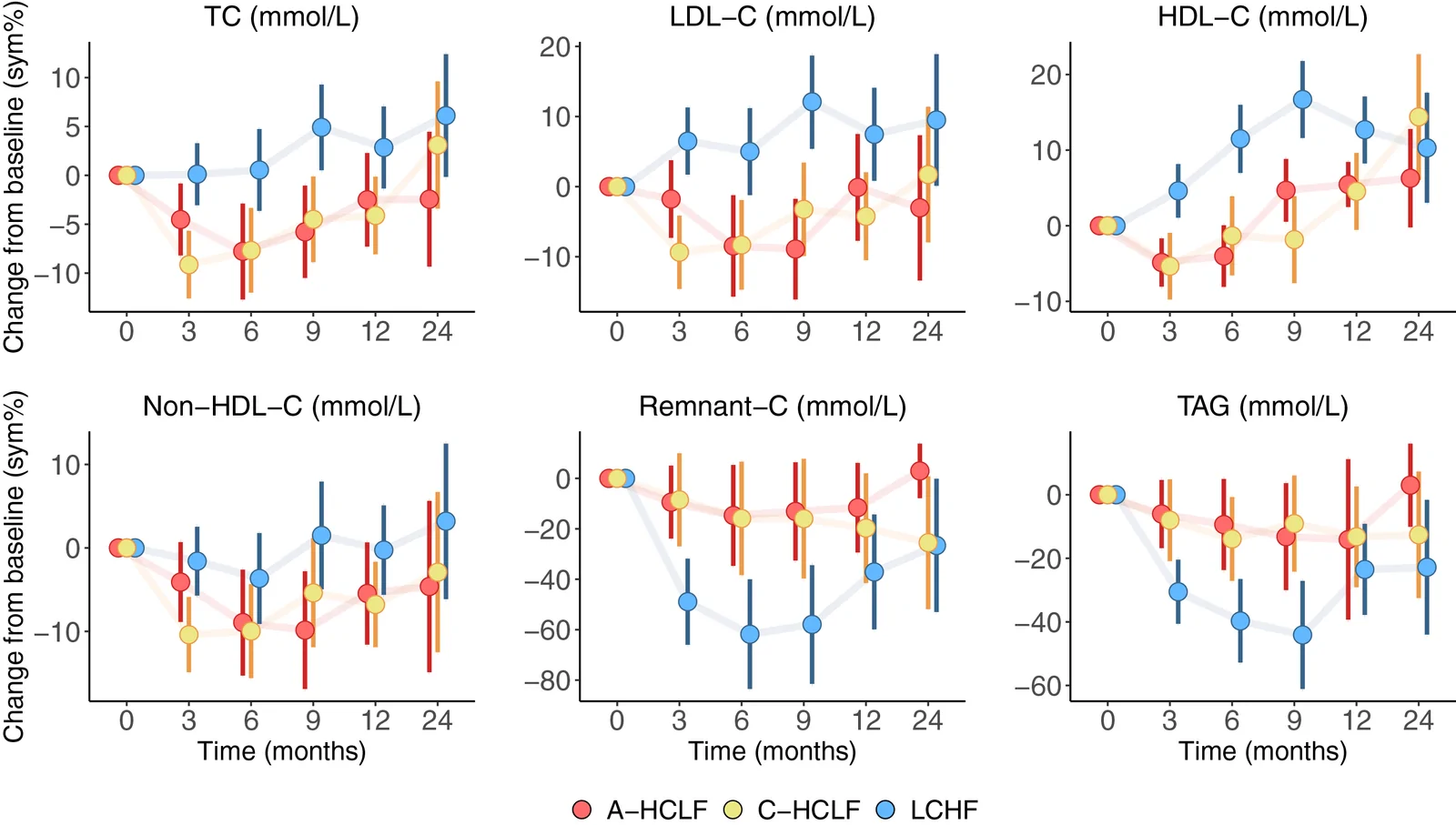
Relative model-adjusted changes from baseline to follow-ups in standard lipids, presented as sympercents (s%) with 95% confidence intervals. Values were natural log-transformed and analysed with constrained linear mixed-effects models adjusted for baseline values, age, sex, and couple-randomisation status, with time and group-by-time interaction included in the models. Between-group estimates and corresponding 95% confidence intervals are reported in Supplementary Table S4. The numbers of participants with available plasma data at baseline and 3, 6, 9, 12 and 24 months were 61, 24, 18, 18, 14 and 9 in the A-HCLF group; 60, 26, 24, 19, 21 and 10 in the C-HCLF group; and 53, 31, 21, 17, 17 and 9 in the LCHF group, respectively. Abbreviations: A-HCLF, acellular high-carbohydrate low-fat; C-HCLF, cellular high-carbohydrate low-fat; HDL-C, high-density lipoprotein cholesterol; LCHF, low-carbohydrate high-fat; LDL-C, low-density lipoprotein cholesterol; remnant-C, remnant cholesterol; s%, sympercent; TAG, triacylglycerol; TC, total cholesterol.

Furthermore, we calculated non-HDL-C, which encompasses the cholesterol content of all apoB-containing lipoproteins. A significant between-group difference between the LCHF and A-HCLF groups was observed only at 9 months (LCHF vs. A-HCLF at 9 months: +1.51% [+0.029 mmol/L] vs. −9.38% [−0.33 mmol/L], p = 0.019). A significant between-group difference from baseline was also found between the LCHF and the A-HCLF group at 3, 6, 9 and 24 months for TAGs (LCHF vs. A-HCLF at 24 months: −20.4% [−0.25 mmol/L] vs. −3.06% [−0.029 mmol/L], p = 0.037) and remnant-C (−23.3% [−0.21 mmol/L] vs. +3.01% [+0.066 mmol/L], p = 0.041) (Fig. 3, Supplementary Table S4). Significantly greater reductions in TAGs and remnant-C in the LCHF group were additionally observed at 12 months, but only after adjustment for diet adherence (Supplementary Table S10) and not in the primary ITT analysis (Supplementary Table S4). Within the LCHF group, TAGs and remnant-C significantly declined throughout the whole 24-month intervention period, while in the C-HCLF carbohydrate group, TAG concentrations were significantly reduced at 6 months only. A significantly greater reduction was observed in the C-HCLF group compared with the A-HCLF group only for LDL-C at 3 months and remnant-C at 24 months. No other significant between-group differences were observed between the C-HCLF and A-HCLF groups for other standard lipid measures at any time point (Fig. 3, Supplementary Table S4).

### Lipoprotein particles and LDL subfractions

To gain more detailed insight into the dynamic responses in lipid metabolism, we measured concentrations of different lipoprotein particles and major LDL subfractions. For total VLDLs, significant between-group differences in change scores were found between the LCHF and A-HCLF groups from baseline to 3, 6, 9 and 24 months (LCHF vs. A-HCLF at 24 months: −15.4% [−11.6 nmol/L] vs. +8.41% [+5.59 nmol/L], p = 0.014), and between the C-HCLF and A-HCLF groups only at 24 months (C-HCLF vs. A-HCLF at 24 months: −18.5% [−16.6 nmol/L] vs. +8.41% [+5.59 nmol/L], p = 0.004). After adjustment for diet adherence there was a significantly greater reduction in VLDLs on the LCHF compared to the A-HCLF diet also at 12 months (Supplementary Table S10), but not in the primary ITT analysis (Supplementary Table S5). Within the LCHF group, there was a large and significant reduction from baseline in total VLDLs at each timepoint up to 12 months, while VLDLs increased significantly within the A-HCLF group and decreased significantly within the C-HCLF group at 24 months only. No between-group differences were observed for total IDLs, whereas total LDLs differed between the LCHF and A-HCLF groups at 6 and 9 months, but only the 9-month difference remained significant after adjustment for diet adherence (Fig. 4, Supplementary Tables S5 and S10).

**Fig. 4:**
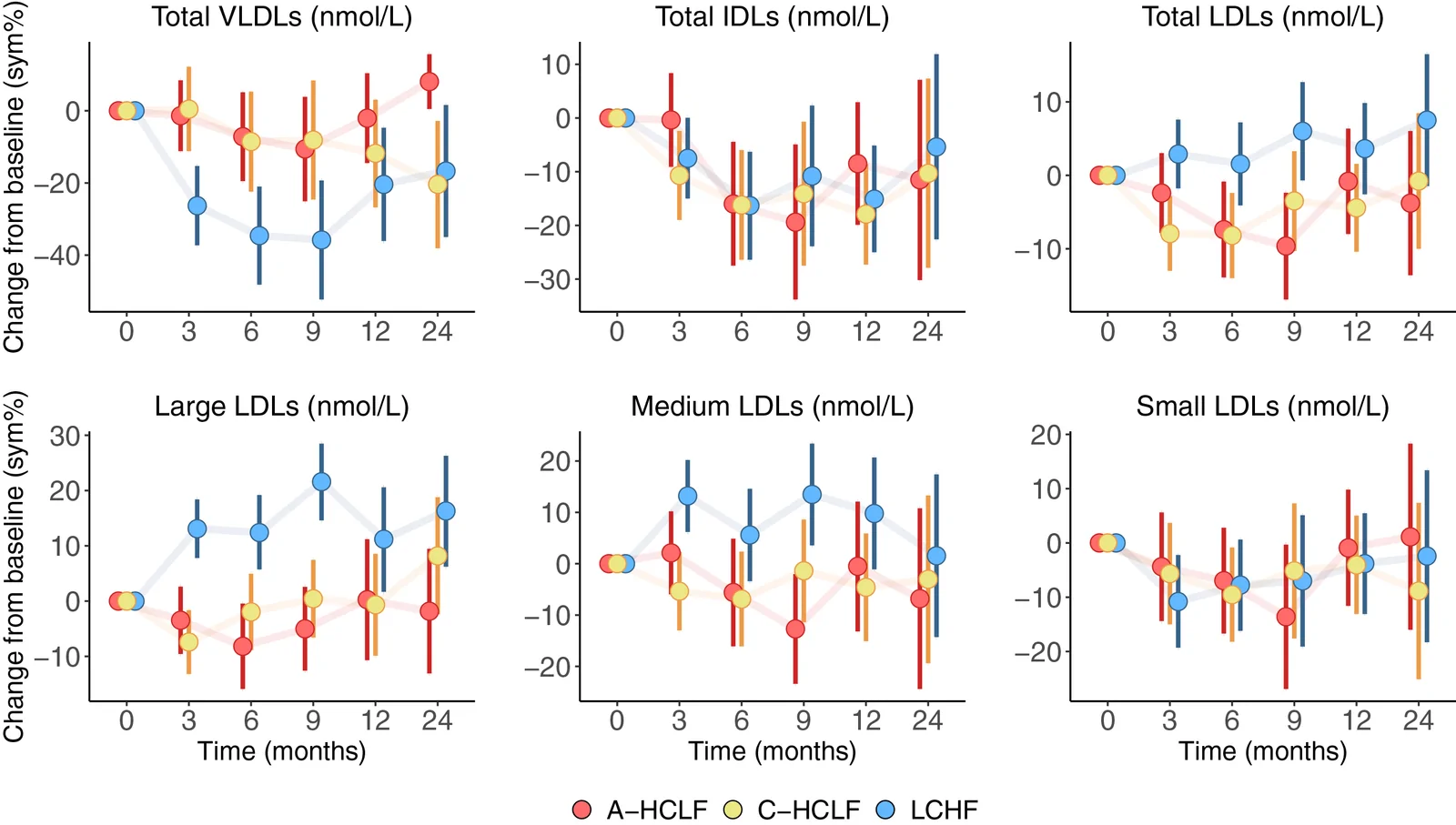
Relative model-adjusted changes from baseline to follow-ups in lipoprotein particles and LDL subclass particles, presented as sympercents (s%) with 95% confidence intervals. Values were natural log-transformed and analysed with constrained linear mixed-effects models adjusted for baseline values, age, sex, and couple-randomisation status, with time and group-by-time interaction included in the models. Between-group estimates and corresponding 95% confidence intervals are reported in Supplementary Table S5. The numbers of participants with available plasma data at baseline, 3, 6, 9, 12 and 24 months were 61, 24, 18, 18, 14 and 9 in the A-HCLF group; 60, 26, 24, 19, 21 and 10 in the C-HCLF group; and 53, 31, 21, 17, 17 and 9 in the LCHF group, respectively. Abbreviations: A-HCLF, acellular high-carbohydrate low-fat; C-HCLF, cellular high-carbohydrate low-fat; IDLs, intermediate-density lipoprotein particles; LCHF, low-carbohydrate high-fat; LDLs, low-density lipoprotein particles; s%, sympercent; VLDLs, very-low-density lipoprotein particles.

For the major LDL subfractions, a differential response in relative change scores from baseline to 3, 6, 9 and 24 months was observed between the LCHF group and A-HCLF group for large LDLs (LCHF vs. A-HCLF at 24 months: +17.7% [+63.9 nmol/L] vs. −1.81% [−15.7 nmol/L], p = 0.018) and from baseline to 3 and 9 months for medium LDLs (LCHF vs. A-HCLF at 9 months: +14.5% [+52.0 nmol/L] vs. −11.9% [−36.6 nmol/L], p < 0.001), but not at any timepoint for small LDLs in any of the group comparisons. In the diet adherence adjusted analysis, additional significant between-group differences were observed for medium LDLs between LCHF and A-HCLF at 6 months (Supplementary Table S10). Within the LCHF group, a significant increase in large LDLs was observed throughout the timepoints up to 24 months, while a significant reduction was observed in the C-HCLF group at 3 months and in the A-HCLF group at 6 months. For small LDLs, a reduction was observed in all diet groups throughout the intervention, but only significantly in the LCHF at 3 months, in the C-HCLF group at 6 months, and in the A-HCLF group at 9 months. At 9 months, there was a significant reduction in a broad spectrum of LDLs (total, medium, and small LDLs) in the A-HCLF group (Fig. 4, Supplementary Table S5).

### Apolipoprotein B and lipid ratios

For apoB, we found a significant between-group difference in relative change scores only at 9 months between the LCHF group and A-HCLF group (LCHF vs. A-HCLF at 9 months: +2.57% [+0.014 g/L] vs. −9.00% [−0.071 g/L], p = 0.014), although this was no longer significant after adjusting for diet adherence (Fig. 5, Supplementary Tables S6 and S10). No significant change in apoB was observed within the LCHF group at any timepoint. Within the carbohydrate groups, a significant reduction in apoB was observed in the C-HCLF group at 3 and 6 months, and at 6 and 9 months in the A-HCLF group (Fig. 5, Supplementary Table S6).

**Fig. 5:**
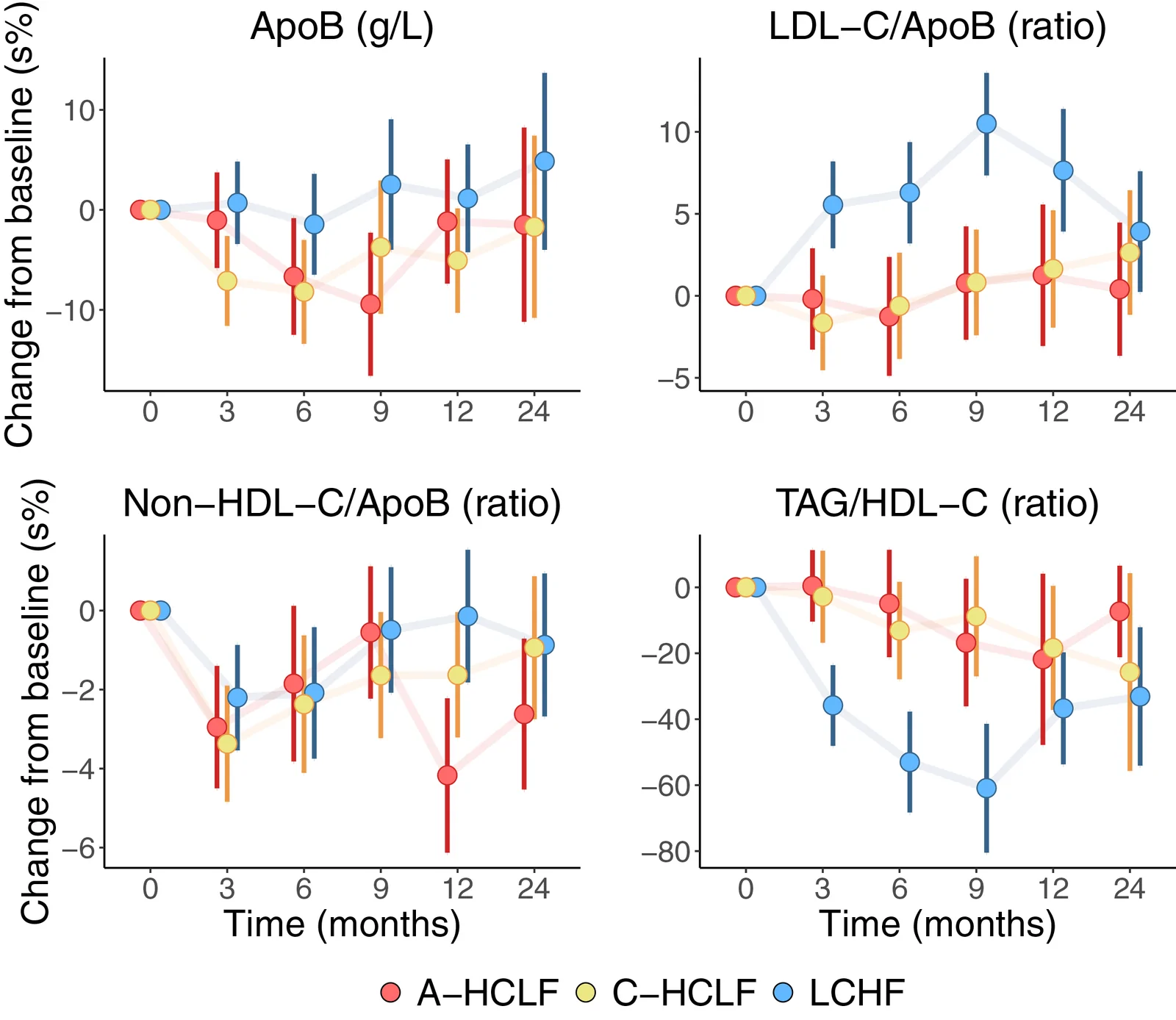
Relative model-adjusted changes from baseline to follow-ups in apoB and lipid ratios, presented as sympercents (s%) with 95% confidence intervals. Values were natural log-transformed and analysed with constrained linear mixed-effects models adjusted for baseline values, age, sex, and couple-randomisation status, with time and group-by-time interaction included in the models. Between-group estimates and corresponding 95% confidence intervals are reported in Supplementary Table S6. The numbers of participants with available plasma data at baseline, 3, 6, 9, 12 and 24 months were 61, 24, 18, 18, 14 and 9 in the A-HCLF group; 60, 26, 24, 19, 21 and 10 in the C-HCLF group; and 53, 31, 21, 17, 17 and 9 in the LCHF group, respectively. Abbreviations: A-HCLF, acellular high-carbohydrate low-fat; apoB, apolipoprotein B; C-HCLF, cellular high-carbohydrate low-fat; HDL-C, high-density lipoprotein cholesterol; LCHF, low-carbohydrate high-fat; LDL-C, low-density lipoprotein cholesterol; s%, sympercent; TAG, triacylglycerol.

Furthermore, we examined three surrogate markers derived from standard lipid measures: the LDL-C/apoB ratio, indicating cholesterol content per LDL particle and hence LDL size; the non-HDL-C/apoB ratio, indicating average cholesterol content per apoB-containing particle across LDL, VLDL, IDL, and Lp(a); and the TAG/HDL-C ratio, a proxy for LDL particle size and insulin resistance. For the LDL-C/apoB ratio, we found a significant difference in relative change scores from baseline to 3, 6, 9 and 12 months between the LCHF group and the A-HCLF group (LCHF vs. A-HCLF at 12 months: +7.94% [+0.10] vs. +1.26% [+0.016], p = 0.024). Within the LCHF group, the LDL-C/apoB ratio significantly increased throughout the whole 24-month intervention period, while no significant changes were observed in the A-HCLF and C-HCLF groups. For non-HDL-C/apoB ratio, a significant between-group difference was observed only at 12 months between the LCHF and A-HCLF groups (LCHF vs. A-HCLF at 12 months: −0.14% [−0.003] vs. −4.09% [−0.07], p = 0.020) and between the C-HCLF and A-HCLF groups (C-HCLF vs. A-HCLF at 12 months: −1.61% [−0.027] vs. −4.09% [−0.07], p = 0.043) (Fig. 5, Supplementary Table S6).

For the TAG/HDL-C ratio, we found a large and significant group difference in change score from baseline to 3, 6, 9 and 24 months between the LCHF and A-HCLF groups (LCHF vs. A-HCLF at 24 months: −28.2% [−0.69] vs. −7.05% [−0.34], p = 0.043). After adjustment for diet adherence, a significant between group difference was also observed at 12 months between the LCHF and A-HCLF groups (Supplementary Table S10). Within groups, a marked and significant reduction was observed within the LCHF group throughout the whole 24-month intervention period, while no significant changes were observed within the C-HCLF or A-HCLF groups (Fig. 5, Supplementary Table S6).

### HDL particle composition and distribution

To gain insight into the composition of HDLs, we measured total apoA1 and total apoA2, as well as their distribution within distinct HDL subfractions. For total apoA1, we found a significant between-group difference in change scores from baseline to 3, 6 and 12 months between the LCHF and A-HCLF groups (LCHF vs. A-HCLF at 12 months: +7.61% [+0.10 g/L] vs. +1.96% [+0.031 g/L], p = 0.028) and between the C-HCLF and A-HCLF groups only from baseline to 9 months (C-HCLF vs. A-HCLF at 9 months: −2.37% [−0.03 g/L] vs. +2.71% [+0.040 g/L], p = 0.049). Within groups, apoA1 significantly increased in the LCHF group at 6, 9 and 12 months, while a significant decrease was found in the A-HCLF and C-HCLF groups at 3 months, and then a significant increase at 24 months in the C-HCLF group (Fig. 6, Supplementary Table S7).

**Fig. 6:**
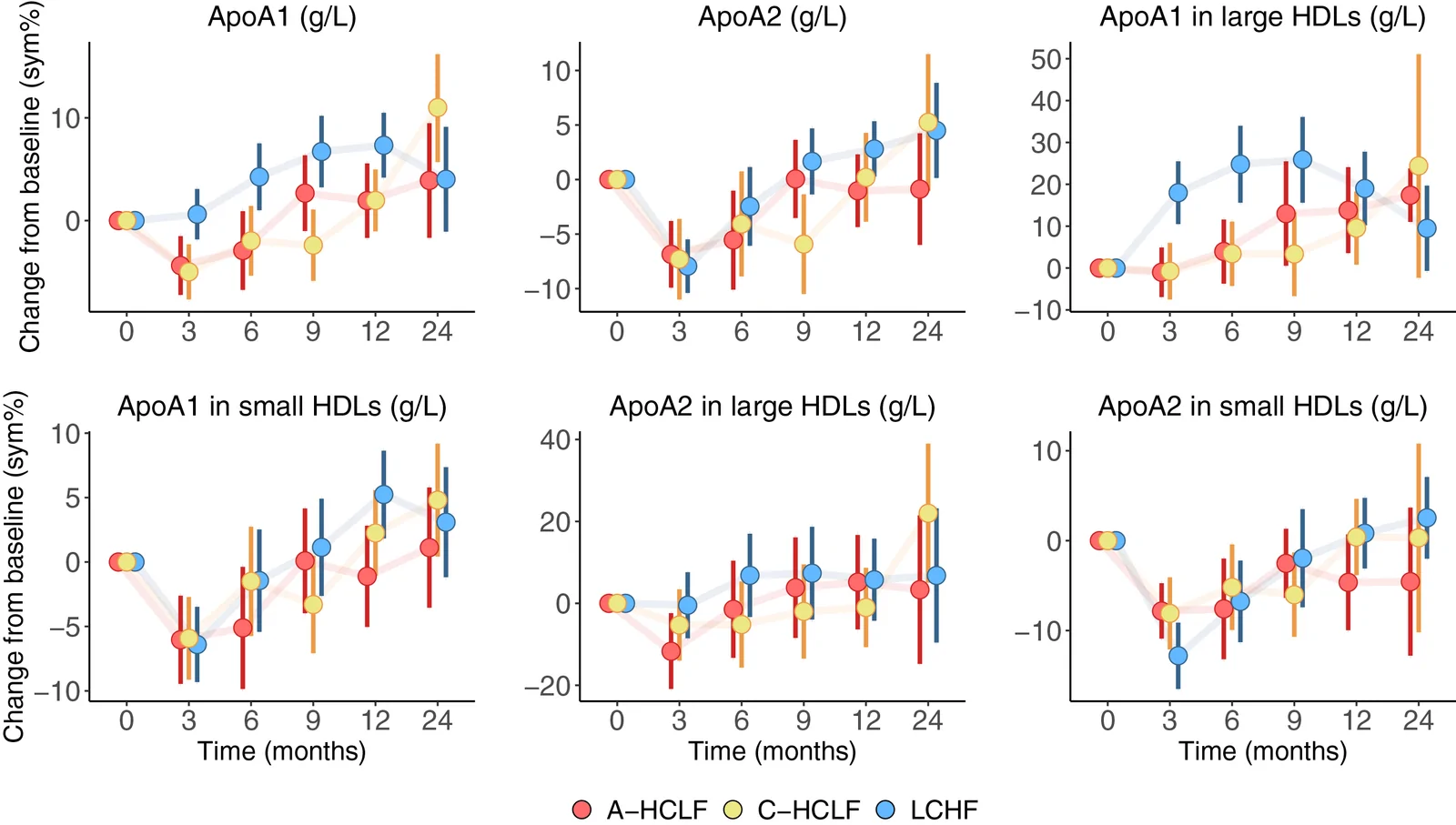
Relative model-adjusted changes from baseline to follow-ups in apoA1 and apoA2 within HDL subfractions, presented as sympercents (s%) with 95% confidence intervals. Values were natural log-transformed and analysed with constrained linear mixed-effects models adjusted for baseline values, age, sex, and couple-randomisation status, with time and group-by-time interaction included in the models. Between-group estimates and corresponding 95% confidence intervals are reported in Supplementary Table S7. The numbers of participants with available plasma data at baseline, 3, 6, 9, 12 and 24 months were 61, 24, 18, 18, 14 and 9 in the A-HCLF group; 60, 26, 24, 19, 21 and 10 in the C-HCLF group; and 53, 31, 21, 17, 17 and 9 in the LCHF group, respectively. Abbreviations: A-HCLF, acellular high-carbohydrate low-fat; apoA1, apolipoprotein A1; apoA2, apolipoprotein A2; C-HCLF, cellular high-carbohydrate low-fat; HDL, high-density lipoprotein; LCHF, low-carbohydrate high-fat; s%, sympercent.

For apoA1 in large HDLs, we found a significant between-group difference in change scores from baseline to 3 and 6 months between the LCHF and A-HCLF groups (LCHF vs. A-HCLF at 6 months: +28.2% [+0.083 g/L] vs. +4.03% [+0.019 g/L], p = 0.001), and for apoA1 in small HDLs only at 12 months (LCHF vs. A-HCLF at 12 months: +5.37% [+0.05 g/L] vs. −1.10% [−0.012 g/L], p = 0.014). After adjustment for diet adherence, the between-group difference for apoA1 in large HDLs was also significant at 9 and 12 months between the LCHF and A-HCLF groups (Supplementary Table S10). Within the LCHF group, apoA1 in large HDLs significantly increased up to 12 months, while apoA1 in small HDLs showed a significant decrease at 3 months before increasing at 12 months. In the A-HCLF group, apoA1 in large HDLs increased significantly at 9, 12 and 24 months, while in the C-HCLF group, apoA1 in large HDLs increased significantly only at 12 months. ApoA1 in small HDLs decreased in the A-HCLF group at 3 and 6 months, and in the C-HCLF group only at 3 months. In the primary analysis, a significant between-group difference in total apoA2 was observed between the C-HCLF and A-HCLF groups at 9 months, while apoA2 in small HDLs differed significantly between the LCHF and A-HCLF groups at 3 months (Fig. 6, Supplementary Table S7). After adjustment for diet adherence, the between-group difference in apoA2 in small HDLs at 3 months did not reach statistical significance, whereas apoA2 in large HDLs showed a significant between-group difference between the LCHF and A-HCLF groups at 3 months (Supplementary Table S10).

### Coronary artery calcium score

Finally, we analysed changes in CAC score data obtained by computed tomography (CT) imaging at 6 and 12 months. Overall, there were no notable changes observed in CAC score across any of the diet groups. The majority of the participants had a CAC score equal to 0 and did not exhibit any deterioration throughout the study period. Among the few individuals showing a deterioration in CAC score, it was notable that they also presented higher CAC score at baseline, indicating pre-existing arterial calcification (Table 2, Supplementary Table S8).

**Table 2:** CAC score progression.^a^

	Baseline		6M		12M	
**Diet**	n	CAC = 0	n	CAC = 0	n	CAC = 0
A-HCLF	67	59 (88%)	20	14 (70%)	14	10 (71%)
LCHF	63	54 (86%)	29	23 (79%)	21	15 (71%)
C-HCLF	62	51 (81%)	26	19 (73%)	22	16 (73%)

Abbreviations: A-HCLF, acellular high-carbohydrate low-fat; CAC, coronary artery calcium; C-HCLF, cellular high-carbohydrate low-fat; LCHF, low-carbohydrate high-fat; M, months.

Summary of all participants with CAC score >0 is shown in Supplementary Table S8.

a Table is showing the total number of participants with available CAC score measure and the number and percentage of participants with a CAC of 0 at baseline and 6 and 12 months across each diet group.

### Sensitivity/exploratory analyses

In the exploratory sensitivity analyses adjusting for diet adherence, the overall pattern of between-group differences was similar to that observed in the primary ITT analysis, with some additional nominally significant between-group differences, most notably at 12 months as noted above (Supplementary Table S10). In the IPW sensitivity analyses applied to assess robustness to potential dropout-related bias, we found overall minimal changes in nominal significance status. Only sporadic changes from significance to non-significance were observed for TC at 6 months for LCHF vs. A-HCLF, remnant-C at 24 months for both LCHF and C-HCLF vs. A-HCLF, total VLDLs at 24 months for C-HCLF vs. A-HCLF, total LDLs at 6 months for LCHF vs. A-HCLF, medium LDLs at 3 months for LCHF vs. A-HCLF, non-HDL-C/apoB at 12 months for C-HCLF vs. A-HCLF, and TAG/HDL-C at 24 months for LCHF vs. A-HCLF (Supplementary Table S11).

Although not prespecified, we performed a post hoc exploratory comparison between the C-HCLF and LCHF groups for key lipid outcomes. As for the A-HCLF comparison, significant between-group differences in relative change scores were observed for TAG-rich lipoprotein markers, including VLDLs, TAGs, remnant-C, and TAG/HDL-C ratio, from baseline to 3, 6 and 9 months (C-HCLF vs. LCHF at 9 months: +31.9%, +41.9%, +52.2%, and, +68.6%, respectively). After diet adherence adjustment, these differences were also significant at 12 months (Supplementary Table S12). This pattern primarily reflected pronounced reductions in these markers in the LCHF group and no or minimal change in the C-HCLF group (Supplementary Tables S4 and S5). Significant between-group differences in relative change scores were also observed for TC, LDL-C, HDL-C, apoA1, and the LDL-C/apoB ratio from baseline to 3, 6, 9 and 12 months (C-HCLF vs. LCHF at 12 months: −6.72%, −11.0%, −7.82%, −5.23%, and −5.82%, respectively), and for apoA1 in large HDLs, large LDLs, and total LDLs from baseline to 3, 6 and 9 months (C-HCLF vs. LCHF at 9 months: −20.2%, −19.0%, and −9.05%, respectively). After diet adherence adjustment, additional significant between-group differences were observed at 12 months for apoA1 in large HDLs and for large LDLs, whereas the between-group differences for total LDLs were no longer significant at any timepoint. For apoB and non-HDL-C, significant between-group differences in relative change scores were observed at 3 months (C-HCLF vs. LCHF: −7.51% and −8.44%, respectively), although these did not remain significant after diet adherence adjustment (Supplementary Table S12).

## Discussion

In the present study, we examined detailed lipid profile changes in standard lipids, major lipoprotein subfractions, and apolipoproteins in individuals with obesity following distinct dietary regimens differing in proportions of carbohydrates, total fat, and SFAs, as well as carbohydrate quality. Notably, the LCHF diet, providing nearly one-third of energy from SFAs, was associated with pronounced reductions in VLDLs, TAGs, and remnant-C, along with increases in HDL-C, apoA1, large LDL particles, and LDL-C, without significant changes in apoB. In contrast, reductions in TC and LDL-C levels were observed with the carbohydrate-based diets, with no consistent impact on the other lipoprotein-lipid markers. No diet-dependent changes in CAC score were seen at 6 or 12 months. The longer-term lipid and lipoprotein findings should be interpreted with caution considering the high attrition rate, particularly between 12 and 24 months.

The substantial reductions in VLDLs and TAGs observed in the LCHF group are noteworthy, as elevated levels of these represent a central lipoprotein abnormality in the insulin resistance syndrome, where increased small LDLs and reduced HDL-C often are consequential events.^9^ In the LCHF group, fasting plasma TAGs were lowered by 20–35% despite relatively normal TAG values at baseline, highlighting the sensitivity of TAG levels to dietary carbohydrate amounts. Notably, no overall significant changes in TAGs were observed on the higher-carbohydrate diets despite similar weight loss as for the LCHF diet, supporting macronutrient composition as a primary determinant of TAGs. Previous studies of people with obesity have shown a TAG-lowering effect of carbohydrate reduction^29^, ^30^ and a TAG-increasing effect of higher carbohydrate intake.^31^, ^32^, ^33^, ^34^ The TAG-raising effect of carbohydrates may partly involve an increase in hepatic *de novo* lipogenesis, promoting secretion of TAG-rich VLDLs. This condition, also known as carbohydrate-induced hypertriglyceridemia,^33^ may particularly be exacerbated by fructose and other simple sugars.^34^ In our study, however, a reduction in added sugar from approximately 6 E% at baseline to a mere 1–2 E% maximum at follow-ups was insufficient to significantly impact TAG concentrations in the carbohydrate groups, and the quality of carbohydrates (A-HCLF vs. C-HCLF) also had no significant impact on TAG levels. Mechanistically, the more complex carbohydrates at baseline and on the A-HCLF and C-HCLF diets may have maintained a lower VLDL-TAG turnover relative to lower-carbohydrate intake, as supported by a previous study investigating the effect of a whole-food carbohydrate-based diet on VLDL kinetics, where VLDL-TAG turnover was reduced without the expected rise in *de novo* lipogenesis that is typically observed with higher intake of simple sugars.^32^

In the context of a low-carbohydrate diet, where glycogen stores are relatively depleted, the body undergoes metabolic and hormonal adaptations to utilise fat as the primary energy source. Low insulin levels promote lipolysis in adipose tissues, leading to increased delivery of non-esterified fatty acids (NEFAs) to the liver. While the precise kinetics of VLDL metabolism remain incompletely understood, cell culture studies have shown that enhanced NEFA influx to the liver inhibits degradation of apoB (the main protein in VLDLs, IDLs, and LDLs), thereby promoting VLDL assembly and secretion.^35^ However, recent experiments using primary hepatocytes isolated from rat liver show that low insulin levels (such as during fasting or on a low-carbohydrate diet) may, via the phosphatidic acid biosynthetic pathway, suppress the transport of lipid droplets to the smooth endoplasmic reticulum, the site of lipoprotein assembly.^36^ This reduces the release of VLDLs and TAGs into an already NEFA-rich bloodstream, providing a possible explanation for the reduction in these markers following the LCHF diet in our study.

Another possible mechanism underlying the observed reduction in VLDLs and TAGs in the LCHF group involves increased peripheral lipolysis (turnover) of TAGs from VLDL, a process mediated by lipoprotein lipase (LPL).^10^ Low insulin levels typically associated with LCHF diets have been shown to increase muscle LPL activity,^37^, ^38^ facilitating efficient delivery of TAGs to peripheral tissues, such as skeletal muscles and the heart, for energy production when glucose availability is limited. Efficient VLDL turnover is further proposed as a key mechanism contributing to the observed elevation in LDL-C and HDL-C on low-carbohydrate diets.^10^ Moreover, when circulating TAG levels are low, the liver secretes rather cholesterol-rich apoB-containing lipoproteins, such as smaller VLDLs and a greater proportion of directly secreted LDLs.^39^ Concurrently, reduced activity of cholesteryl ester transfer protein (CETP) in the context of lower TAG levels limits the exchange of cholesteryl esters and TAGs between apoB-containing lipoproteins.^40^ This leaves more cholesteryl esters in LDLs and HDLs, resulting in larger LDLs while securing adequate HDL-C levels, as observed in the LCHF group.

These mechanisms were further elucidated through exploration of lipoprotein subfraction particles, providing more detailed insight into the distribution of LDLs and HDLs across the diet groups and their connection to CVD risk beyond traditional cholesterol measures.^41^ While LDL-C increased by 5–13% in the LCHF group, peaking at 9 months with a mean concentration of 3.42 mmol/L, this increase involved large LDLs with no change in apoB, likely reflecting the lower TAG levels and reduced CETP-mediated lipid exchange. Importantly, larger LDLs have been overall less strongly associated with CVD compared with small LDLs,^41^ although the precise role of LDL particle size in CVD remains debated and may be attenuated after adjustment for total LDL particle number (i.e., apoB).^42^ Small LDLs have been reported to display potentially more atherogenic properties, including greater susceptibility to oxidation and conformational changes in apoB.^41^, ^43^, ^44^, ^45^ At the same time, a predominance of small LDLs is closely associated with the insulin resistance syndrome and often with higher circulating particle numbers, and their association with atherosclerosis may also, in part, reflect broader metabolic disturbances that are central in atherogenesis.^8^, ^46^ Furthermore, evidence for the cardioprotective benefits of elevated HDL-C is conflicting, partly due to heterogeneity in the size and composition of HDLs.^47^ In the present study, the increase in HDL-C observed in the LCHF group was accompanied by a significant increase in total apoA1 and in apoA1 within large HDLs, which have been associated with enhanced HDL functionality and cardioprotective benefits.^47^

Proper and effective regulation of lipoprotein kinetics is essential not only for maintaining energy homoeostasis according to varying physiological and nutritional needs, but also for preventing accumulation of partially metabolised lipoprotein particles such as small LDLs and remnant-C (the cholesterol component of TAG-rich lipoproteins, such as VLDLs and chylomicrons). The significant reduction in remnant-C in the LCHF group is notable given that results from observational studies have shown that TAGs and remnant-C, rather than LDL-C, independently predict CVD outcomes, with remnant-C being associated with increased risk for coronary heart disease irrespective of LDL-C or apoB levels.^48^, ^49^ Participants with discordantly high remnant-C and low LDL-C also had higher risk for atherosclerotic disease, whereas those presenting with an opposite discordance of low remnant-C and high LDL-C did not show elevated risk.^49^ Recent analyses of the Copenhagen General Population Study and UK Biobank further show that elevated remnant-C, but not LDL-C, is associated with increased risk of recurrent ASCVD events, including among individuals both with and without impaired renal function.^50^ This is further supported by findings that remnant-C, and increased TAG content in LDLs, but not LDL-C itself, correlates with systemic low-grade inflammation and vascular damage.^51^, ^52^ The heightened risk of remnant-C may partly be due to its direct uptake by macrophages, unlike LDLs which generally require modification (e.g., oxidation) first.^53^ These data suggest that elevated CVD risk may be more strongly reflected by by-products of incomplete metabolic processes such as elevated concentrations of remnant-C and small LDLs, than by LDL-C per se.

A more complex interplay among components of lipid metabolism with respect to cardiometabolic disease risk is further illustrated by the joint consideration of LDL-C and apoB, as well as HDL-C and TAGs, where different risk factor combinations may reflect distinct metabolic milieus that influence overall risk. Firstly, an RCT of LDL-C lowering by statin treatment demonstrated that patients with elevated TAGs and low HDL-C experienced greater reductions in coronary events than those having isolated LDL-C elevation, supporting that the broader lipid phenotype modifies the absolute benefit associated with LDL-C lowering.^54^ Secondly, higher levels of LDL-C relative to apoB (LDL-C/apoB ratio), which reflects LDL particle size and the efficiency of lipid turnover,^55^ have been found to predict lower rates of incident metabolic syndrome^56^ and to associate inversely with major cardiovascular events in individuals with established atherosclerosis.^15^ The significantly greater increase in the LDL-C/apoB ratio on the LCHF diet may therefore suggest a metabolic context of increased lipid turnover that might impact risk associated with absolute levels of these markers. Along with the concomitant reduction in TAGs, remnant-C, and VLDL particles, the increased LDL-C/apoB ratio is consistent with a shift on the LCHF diet toward larger, more cholesterol-rich LDL particles rather than a proportional increase in circulating apoB-containing particle number. Accordingly, recent conceptual and clinical reviews, although hypothetical, have proposed that LDL-C elevations during carbohydrate-restricted diets—usually higher in SFAs—may reflect adaptive changes in lipid trafficking, energy metabolism, and cell membrane fluidity, rather than a straightforward increase in atherogenic burden.^10^, ^57^, ^58^ Nevertheless, despite the redistribution towards large LDLs, no significant increase in apoB, and greater reductions in TAG-rich lipoproteins on the LCHF diet, the concomitant 5–10% increase in LDL-C could be clinically relevant, considering the lower CVD risk at lower LDL-C and apoB levels demonstrated in pharmacological and Mendelian randomisation studies.^59^, ^60^, ^61^, ^62^ Also, it should be noted that pharmacological reduction of TAGs and remnant-C by 20–30% has not consistently been found to lower CVD risk,^63^, ^64^ although an independent causal role of not only apoB but also TAG-rich lipoproteins is supported by Mendelian randomisation studies.^65^ Further cardiovascular outcome data from emerging TAG-lowering therapies, e.g., APOC3 inhibitors, may help clarify the clinical relevance of TAG lowering.^66^ Still, it must be considered that these therapies lower not only TAG-rich lipoproteins by 60–70% but also apoB by approximately 15%. Additionally, caution may be warranted when extrapolating findings from pharmacological interventions targeting specific lipid markers, including LDL-C and TAGs, to dietary patterns such as an LCHF diet which induce broader metabolic adaptations.

Within the broader metabolic context, the decreased TAG/HDL-C ratio on the LCHF diet relative to the A-HCLF comparator, along with the reduction in visceral fat mass by 10–15%,^18^ suggests more pronounced changes in lipid and lipoprotein markers commonly linked to the insulin resistance syndrome, which is an important independent CVD risk factor.^67^ However, improvements in insulin resistance assessed by HOMA2-IR were similar across the three diet groups, and, relative to the A-HCLF comparator, the LCHF diet showed a significant reduction in systolic blood pressure only at 9 months. This suggests that the LCHF diet particularly affected insulin-regulated lipid and lipoprotein metabolism, whereas improvements in glucose homoeostasis and blood pressure may relate more to similar weight loss on all diets.

Furthermore, CAC score provides a direct measure of calcified coronary plaque burden and a clinically relevant complement to circulating lipid markers.^68^ In our study, no diet-dependent changes in CAC score were observed at 6 or 12 months, despite differences in LDL-C between the diets. A discordance of LDL-C and CAC scores is consistent with data from the MESA prospective cohort study, showing that 37% of male participants with relatively high LDL-C (>190 mg/dL, i.e., 4.9 mmol/L) and a mean age of 63 (SD 9) still exhibited a CAC score of zero,^69^ suggesting low risk of future coronary events despite high LDL-C. However, the literature remains limited on how diet-induced LDL-C elevations in the context of concurrent changes in TAG-rich lipoproteins and HDL-related markers influence CAC progression over time. Thus, while the disconnect of CAC score progression from LDL-C is noteworthy, the relatively short follow-up duration, high attrition rate, and limited sample size at 12 months preclude firm conclusions regarding long-term atherosclerosis progression or cardiovascular risk. From the perspective of the cumulative cholesterol hypothesis, atherogenic risk is expected to accrue with prolonged exposure to higher LDL-C.^70^

Finally, although the study design did not prespecify comparison of the LCHF to the C-HCLF diet, we explored whether the C-HCLF diet—with a potentially superior carbohydrate quality (e.g., more whole plant foods instead of flour-based foods, albeit similar fibre intake)—might show additional significant differences to the LCHF diet. Overall, this comparison showed results similar to the prespecified analyses using A-HCLF as comparator, suggesting that carbohydrate quantity had a stronger influence than carbohydrate cellularity on several lipid responses, particularly TAG-rich lipoprotein markers.

Strengths of this study include its three-arm randomised controlled design with marked diet changes from baseline and contrasts between diets, repeated measures, balanced inclusion of males and females, comprehensive characterisation of lipoprotein metabolism by NMR spectroscopy alongside concurrent assessment of blood pressure, insulin resistance, anthropometrics, and CAC, detailed evaluation of diet adherence using repeated dietary records and objective ketone measurements, and a comprehensive statistical approach with constrained linear mixed-effects modelling and sensitivity analyses. Nevertheless, our study has limitations. Even though the observed changes included clear patterns of biochemically interrelated lipid parameters, the exploratory evaluation of multiple outcomes increases the likelihood of spurious significant findings. Furthermore, while the CARBFUNC study recruited 3.5 times the initially required sample size for the primary outcome measure^18^ (change in visceral fat volume; n = 18 per group) to detect a statistically significant group difference, actual loss to follow-up exceeded expectations, leading to a limited number of participants providing outcome measures at 12 and especially at 24 months. To preserve the benefits of randomisation, we employed an ITT analysis, including all randomised participants in the analyses, regardless of whether they completed the study or adhered to the prescribed diet. Nonetheless, high attrition, declining diet adherence (particularly between 12 and 24 months), and reliance on partly self-reported intake may introduce uncertainty and potential bias in long-term estimates. Given potential bias related to diet adherence and dropout, we performed sensitivity analyses with exploratory adjustment for diet adherence and using inverse probability weighting sensitivity analyses. Although we cannot entirely rule out that data were not missing at random, including possible diet-specific sex differences in attrition, these analyses gave highly similar results, with the most notable exception that the reductions in TAGs, remnant-C, VLDLs, and TAG/HDL-C ratio, as well as increase in apoA1 in large HDLs, were significant between the LCHF and A-HCLF diets also at 12 months after adjustment for diet adherence. Moreover, substantial weight loss across all three diets (5–7% over 12 months, with greater fat loss on the LCHF diet at 6 months^18^), may have influenced between-group differences in lipid markers. Also, the participants in our study had on average relatively normal lipid profiles at baseline (e.g., TAG concentrations below 1.4 mmol/L and apoB around 0.9 g/L), and the impact on CVD risk of changes from these values remains uncertain. Lastly, while our study included similar numbers of males and females, the findings may have limited generalisability to more ethnically diverse populations, to individuals with more severe comorbidities or to those without obesity.

In conclusion, compared with the A-HCLF group, an LCHF diet with 30 E% SFAs showed significantly greater reductions in TAGs and remnant-C, as well as greater increases in LDL-C, LDL-C/apoB ratio, HDL-C, total apoA1, and apoA1 in large HDLs. The increase in LDL-C involved large LDLs. Carbohydrate quality assessed by cellularity had no clear impact on the lipid profile, suggesting carbohydrate amount may be more influential than cellularity for these markers. The lipid profile changes associated with substituting carbohydrates for total- and saturated fats, including no significant increase in apoB or small LDLs, may reflect greater lipid turnover on an LCHF diet. Our study warrants further investigation into the health implications of diet- and SFA-dependent increases in LDL-C in the context of broader dietary, lipid, and metabolic profiles.

## Contributors

Cathrine Loddervik: Investigation, Data curation, Visualisation, Literature search, Writing–original draft preparation. Johnny Laupsa-Borge: Study design, Formal analysis, Software, Data curation, Validation, Literature search, Writing–review & editing. Cathrine H. Sommersten: Project administration, Investigation, Methodology, Writing–review & editing. Kristin Amundsen: Investigation, Writing–review & editing. Arve Ulvik: Writing–review & editing. Kristine Kjerpeseth Johannessen: Investigation, Literature search, Writing–review & editing. Synnøve Halvorsen: Investigation, Literature search, Writing–review & editing. Terje H. Larsen: Methodology, Writing–review & editing. Ottar K. Nygård: Supervision, Writing–review & editing. Jutta Dierkes: Resources, Funding acquisition, Writing–review & editing. Gunnar Mellgren: Supervision, Resources, Funding acquisition, Writing–review & editing. Simon N. Dankel: Conceptualisation, Investigation, Supervision, Project administration, Study design, Methodology, Resources, Funding acquisition, Literature search, Writing–review & editing. Cathrine Loddervik, Johnny Laupsa-Borge, and Simon N. Dankel accessed and verified the underlying data. All authors read and approved the final version.

## Data sharing statement

The individual participant-level biochemical and clinical data described in this manuscript are considered sensitive and cannot be made publicly available due to ethical and data protection considerations. De-identified data may be made available to qualified researchers upon request to the corresponding author, subject to submission of a research proposal and approval by the relevant institutional and ethical authorities. Data will be available following publication of the article.

## Declaration of interests

Jutta Dierkes serves as a reviewer for the Novo Nordisk Foundation's nutrition grant call. All other authors declare no competing interests.

## Appendix A Supplementary data

Supplementary Material

CARBFUNC Protocol
