# Supplementary material for: Lipid dynamics and cardiovascular risk profiles on carbohydrate- or saturated fat-based diets: secondary analysis of the CARBFUNC trial
Source: eBioMedicine. 2026 Oct 24;133:106524. doi: 10.1016/j.ebiom.2026.106524 (PMC13721003; doi:10.1016/j.ebiom.2026.106524)
Supplement: Supplementary file 1 [file mmc2.pdf]

CARBFUNC Clinical Trial Protocol

Protocol Version: 2.0 (retrospective update to comply with the eBioMedicine format)

Protocol Date: November 17, 2025

ClinicalTrials.gov Identifier: NCT03401970

1. Administrative Information

Short Title: Dietary Carbohydrate and Internal Body Fat (CARBFUNC)

Sponsor: Haukeland University Hospital, Bergen, Norway

Co-sponsor / Coordinating Institution: University of Bergen, Department of Clinical Science, Norway

Principal Investigator: Simon N. Dankel, PhD (University of Bergen / Haukeland University Hospital)

Ethics Approval: Regional Ethics Committee (REC West), Norway; approval ID 2017/621 (latest approval aligned with this version).

Trial Registration: ClinicalTrials.gov NCT03401970 (Dietary Carbohydrate and Internal Body Fat).

2. Background and Rationale

Obesity is a major driver of global morbidity and mortality, with cardiovascular disease (CVD) the leading cause of obesity-related deaths (1). Contemporary lipid guidelines emphasise lipid modification to reduce CVD risk (2) and limiting saturated fat intake in dietary advice (3). However, beyond LDL-cholesterol (LDL-C) alone, a broader spectrum of lipoprotein abnormalities—including elevated triglyceride-rich lipoproteins, remnant cholesterol, low HDL-C, and a shift towards small dense LDL—contributes to atherogenesis and residual vascular risk (4–9). Mechanistic and clinical work indicates that carbohydrate restriction can lower triglycerides and remnant cholesterol and alter lipoprotein kinetics (10–16), while inflammatory states and physiological perturbations can also influence lipid measures (17–20). Apolipoprotein-based and particle-informed markers, such as apoB and lipid ratios (e.g., LDL-C/apoB; non-HDL-C/apoB), may refine risk estimation and reflect particle composition and size (5–6, 21–22). Remnant cholesterol has emerged as an independent predictor of CVD risk beyond LDL-C and apoB (23–24), and lipid heterogeneity, including LDL particle size, may modify risk via oxidative susceptibility and metabolic context (8, 25–29). The CARBFUNC trial was designed as a two-year, isocaloric, three-arm randomised trial in adults with obesity to compare: (1) a low-fat high-carbohydrate diet with predominantly acellular (flour-based) carbohydrate sources; (2) a low-fat high-carbohydrate diet with predominantly cellular (minimally processed)

carbohydrate sources; and (3) a very-high-fat low-carbohydrate (LCHF) diet. The primary outcome was visceral adipose tissue volume (30), with prespecified secondary outcomes including detailed plasma lipid and lipoprotein profiles and coronary artery calcium (CAC) score (31–33).

3. Objectives

Primary Objective: To determine the effect of dietary carbohydrate amount and quality versus an LCHF diet on change in visceral adipose tissue up to 24 months (30–31).

Key Secondary Objectives: To compare longitudinal changes in: (a) standard lipids (TC, LDL-C, HDL-C, TAG); (b) NMR-derived lipoprotein subfractions (VLDL, IDL, LDL, HDL and subclasses); (c) apolipoproteins (apoB, apoA1, apoA2); (d) remnant cholesterol; (e) lipid ratios (LDL-C/apoB, non-HDL-C/apoB, TAG/HDL-C); (f) insulin–glucose indices (HOMA2-IR), blood pressure, anthropometry; and (g) CAC at 6 and 12 months .

4. Trial Design

Two-year, three-arm, parallel-group, isocaloric, randomised controlled trial conducted in Bergen, Norway (30). Allocation ratio 1:1:1. Open-label due to the nature of dietary interventions; the trial statistician was blinded to group allocation until primary analyses were completed.

5. Study Setting

Single-centre clinical research setting in Bergen, Norway, with visits at the Research Unit for Health Surveys (RUHS) at the University of Bergen and affiliated hospital laboratories.

6. Eligibility Criteria

Inclusion criteria: Adults aged 20–55 years; BMI ≥30 kg/m^2 and/or waist circumference ≥102 cm (men) or ≥88 cm (women); weight stable (±5%) during prior 2 months; willingness to follow assigned diet and complete study procedures.

Exclusion criteria: Use of statins or anti-diabetic medication; known diabetes; recent surgery or antibiotics (<2 months); chronic inflammatory bowel disease; serious illness; pregnancy or lactation; smoking; excessive alcohol intake (>2 units/day); relevant food allergies.

7. Interventions

Isocaloric diets (women ~2000 kcal/day; men ~2500 kcal/day) with protein ~17 E% and PUFA 7–10 E% across arms.
Acellular carbohydrate diet: 45 E% carbohydrate (predominantly flour-based sources), 38

E% fat (~10 E% SFA).
Cellular carbohydrate diet: 45 E% carbohydrate (minimally processed cellular sources), 38 E% fat (~10 E% SFA).
LCHF diet: 10 E% carbohydrate, 73 E% fat (~30 E% SFA).
Diet plans were delivered via recipes and a digital tool; adherence monitored by 3-day diet records every second week and quarterly self-rated adherence. Ketone bodies were measured as an objective marker of carbohydrate restriction (34).

8. Outcomes

Primary outcome: Change in visceral adipose tissue (CT imaging) (30–31).

Secondary outcomes: Standard lipids (TC, LDL-C, HDL-C, TAG), non-HDL-C; remnant-C; NMR-derived lipoprotein fractions and subclasses; apoB, apoA1, apoA2; lipid ratios (LDL-C/apoB, non-HDL-C/apoB, TAG/HDL-C); anthropometry; blood pressure; HOMA2-IR; CAC (Agatston) . Assessment at baseline, 3, 6, 9, 12, and 24 months for blood/anthropometry; CAC at 0, 6, and 12 months.

9. Schedule of Enrolment, Interventions, and Assessments

**Visit**	**Baseline**	**3 mo**	**6 mo**	**9 mo**	**12 mo**	**24 mo**
Informed consent & eligibility	X					
Randomization & diet start	X					
Anthropometry & blood pressure	X	X	X	X	X	X
Fasting blood (lipids, NMR, apo, ketones, glucose/C-peptide)	X	X	X	X	X	X
CT (visceral fat) & CAC	X		X		X	
Diet records / adherence	—	Every 2 weeks + quarterly adherence questionnaire				

10. Sample Size

Up to 250 participants were planned to ensure adequate precision for outcomes while allowing for attrition over 24 months. The final randomised sample was 193, with analyses including 184 participants with baseline samples for relevant secondary outcomes (intention-to-treat) (30).

11. Assignment of Interventions (Randomization and Blinding)

Computer-generated block randomization (block sizes 6–9), stratified by sex and by household pairing. Due to the dietary nature of the intervention, participants and study staff were unblinded; the trial statistician remained blinded to allocation until all planned analyses were completed.

12. Data Collection, Management, and Monitoring

Standardised anthropometry, blood pressure, fasting blood sampling, NMR lipoprotein profiling, and CT imaging were performed by trained personnel using SOPs (31, 35–36). Data were stored on secure institutional servers with de-identified participant IDs and audit trails. Adverse events related to diet were monitored at visits and by participant contact; serious AEs were reported per institutional policy.

13. Statistical Methods

Analysis populations: Primary analyses used an intention-to-treat approach including all randomised participants with available data. Per-protocol/sensitivity analyses may be reported where prespecified (30).

Primary endpoint analysis: Change in visceral adipose tissue analysed with baseline-adjusted constrained linear mixed-effects models (cLMM), including time, group×time, sex, age, and stratification factor (randomised as couple) with random intercepts/slopes and appropriate covariance structure (37–39).

Secondary endpoints: Continuous outcomes (standard lipids, NMR subfractions, apolipoproteins, ratios, HOMA2-IR) modelled with cLMM on raw or log-transformed scales as appropriate; between-group differences in change scores and within-group changes estimated with 95% CIs. CAC summarised descriptively given zero-inflation (31–33). Two-sided $\alpha=0.05$; multiplicity adjustments not planned for exploratory secondary endpoints (40).

Handling of missing data: Mixed-effects modelling accommodates intermittent missing repeated measures under a missing-at-random assumption; multiple imputation is generally not necessary before mixed modelling for longitudinal trials (41). Dropout patterns will be described; sensitivity analyses may be considered if departures are suspected.

14. Ethics and Dissemination

Approved by REC West (2017/621). Written informed consent obtained from all participants. Interventions consisted of commonly consumed foods; risk was minimal. Results will be disseminated via peer-reviewed publications and scientific meetings; authorship will follow ICMJE criteria (30).

15. Data Access and Availability

Due to the sensitive nature of participant data, de-identified datasets will be available upon reasonable request, subject to a data use application, ethics approval where required, and a data access agreement with the sponsor/institutions.

16. Roles and Responsibilities

The sponsor and coordinating investigators are responsible for study conduct, data integrity, and reporting. An independent statistician performed primary analyses while blinded to allocation.

17. Funding

Trond Mohn Research Foundation; University of Bergen; Kostfonden (Sweden); Norwegian Health Association.

18. Compliance with Journal Guidance

This protocol has been formatted to align with eBioMedicine/Lancet expectations for clinical trials and to reflect EQUATOR recommendations (SPIRIT/CONSORT) for transparency. The document retrospectively summarises approved and implemented procedures for NCT03401970 (30–31).

References

1. Afshin A, Forouzanfar MH, Reitsma MB, Sur P, Estep K, Lee A, et al. Health Effects of Overweight and Obesity in 195 Countries over 25 Years. N Engl J Med. 2017;377(1):13–27.

2. Mach F, Baigent C, Catapano AL, Koskinas KC, Casula M, Badimon L, et al. 2019 ESC/EAS Guidelines for the management of dyslipidaemias: lipid modification to reduce cardiovascular risk. Eur Heart J. 2020;41(1):111–88.

3. Sacks FM, Lichtenstein AH, Wu JHY, Appel LJ, Creager MA, Kris-Etherton PM, et al. Dietary Fats and Cardiovascular Disease: A Presidential Advisory From the American Heart Association. Circulation. 2017;136(3):e1–e23.

4. Soto-Mota A, Flores-Jurado Y, Norwitz NG, Feldman D, Pereira MA, Danaei G, et al. Increased low-density lipoprotein cholesterol on a low-carbohydrate diet in adults with normal but not high body weight: A meta-analysis. Am J Clin Nutr. 2024;119(3):740–7.

5. Johannesen CDL, Mortensen MB, Langsted A, Nordestgaard BG. Apolipoprotein B and Non-HDL Cholesterol Better Reflect Residual Risk Than LDL Cholesterol in Statin-Treated Patients. J Am Coll Cardiol. 2021;77(11):1439–50.

6. Sirimarco G, Labreuche J, Bruckert E, Goldstein LB, Fox KM, Rothwell PM, et al. Atherogenic Dyslipidemia and Residual Cardiovascular Risk in Statin-Treated Patients. Stroke. 2014;45(5):1429–36.

7. Austin MA, King MC, Vranizan KM, Krauss RM. Atherogenic lipoprotein phenotype. Circulation. 1990;82(2):495–506.

8. Fruchart JC, Sacks F, Hermans MP, Assmann G, Brown WV, Ceska R, et al. The Residual Risk Reduction Initiative: a call to action to reduce residual vascular risk in patients with dyslipidemia. Am J Cardiol. 2008;102(10 Suppl):1k–34k.

9. Adiels M, Olofsson S-O, Taskinen M-R, Borén J. Overproduction of Very Low–Density Lipoproteins Is the Hallmark of the Dyslipidemia in the Metabolic Syndrome. Arterioscler Thromb Vasc Biol. 2008;28(7):1225–36.

10. Norwitz NG, Soto-Mota A, Kaplan B, Ludwig DS, Budoff M, Kontush A, et al. The Lipid Energy Model: Reimagining Lipoprotein Function in the Context of Carbohydrate-Restricted Diets. Metabolites. 2022;12(5).

11. Gjuladin-Hellon T, Davies IG, Penson P, Amiri Baghbadorani R. Carbohydrate-restricted diets and LDL-C: meta-analysis. Nutr Rev. 2019;77(3):161–80.

12. Patikorn C, Saidoung P, Pham T, Phisalprapa P, Lee YY, Varady KA, et al. Ketogenic diet: umbrella review of RCTs. BMC Med. 2023;21(1):196.

13. Johnson RK, Appel LJ, Brands M, Howard BV, Lefevre M, Lustig RH, et al. Dietary Sugars Intake and Cardiovascular Health. Circulation. 2009;120(11):1011–20.

14. Parks EJ, Krauss RM, Christiansen MP, Neese RA, Hellerstein MK. Low-fat, high-carbohydrate diet effects on VLDL-TAG kinetics. J Clin Invest. 1999;104(8):1087–96.

15. Parks EJ, Hellerstein MK. Carbohydrate-induced hypertriacylglycerolemia. Am J Clin Nutr. 2000;71(2):412–33.

16. Welsh JA, Sharma A, Abramson JL, Vaccarino V, Gillespie C, Vos MB. Caloric Sweetener and Dyslipidemia. JAMA. 2010;303(15):1490–7.

17. Hourizadeh J, Munshi R, Zeltser R, Makaryus AN. Dietary Effects of Fasting on the Lipid Panel. Curr Cardiol Rev. 2024;20(2):82–92.

18. Wendel M, Paul R, Heller AR. Lipoproteins in inflammation and sepsis. II. Clinical aspects. Intensive Care Med. 2007;33(1):25–35.

19. Taylor R, Zhang C, George D, Kotecha S, Abdelghaffar M, Forster T, et al. Low circulatory levels of total cholesterol, HDL-C and LDL-C are associated with death of patients with sepsis and critical illness: systematic review, meta-analysis, and perspective of observational studies. EBioMedicine. 2024;100:104981.

20. Khovidhunkit W, Kim MS, Memon RA, Shigenaga JK, Moser AH, Feingold KR, et al. Effects of infection and inflammation on lipid and lipoprotein metabolism: mechanisms and consequences to the host. J Lipid Res. 2004;45(7):1169–96.

21. Drexel H, Larcher B, Mader A, Vonbank A, Heinzle CF, Moser B, et al. The LDL-C/ApoB ratio predicts major cardiovascular events in patients with established atherosclerotic cardiovascular disease. Atherosclerosis. 2021;329:44–9.

22. Zhang K, Wei C, Shao Y, Wang L, Zhao Z, Yin S, et al. Association of non-HDL-C/apoB ratio with long-term mortality in the general population: A cohort study. Heliyon. 2024;10(6):e28155.

23. Castañer O, Pintó X, Subirana I, Amor AJ, Ros E, Hernáez Á, et al. Remnant cholesterol and incident CVD. J Am Coll Cardiol. 2020;76(23):2712–24.

24. Quispe R, Martin SS, Michos ED, Lamba I, Blumenthal RS, Saeed A, et al. Remnant cholesterol predicts CVD beyond LDL and ApoB. Eur Heart J. 2021;42(42):4324–32.

25. Berneis KK, Krauss RM. Metabolic origins and clinical significance of LDL heterogeneity. J Lipid Res. 2002;43(9):1363–79.

26. Quesada JA, Bertomeu-González V, Orozco-Beltrán D, Cordero A, Gil-Guillén VF, López-Pineda A, et al. Size and number of lipoprotein particles in risk prediction: systematic review and meta-analysis. Clin Investig Arterioscler. 2023;35(4):165–77.

27. Chary A, Tohidi M, Hedayati M. LDL subfractions and CVD: systematic review. BMC Cardiovasc Disord. 2023;23(1):533.

28. Segrest JP, Jones MK, De Loof H, Dashti N. Structure of apoB-100 in LDL. J Lipid Res. 2001;42(9):1346–67.

29. Tribble DL, Holl LG, Wood PD, Krauss RM. Oxidative susceptibility of LDL subfractions. Atherosclerosis. 1992;93(3):189–99.

30. Sommersten CH, Laupsa-Borge J, Andersen AIO, Fasmer KE, Holmefjord MA, Revheim I, et al. Diets differing in carbohydrate cellularity and amount similarly reduced visceral fat in people with obesity – a randomized controlled trial (CARBFUNC). Clin Nutr. 2022;41(10):2345–55.

31. Agatston AS, Janowitz WR, Hildner FJ, Zusmer NR, Viamonte M, Detrano R. Quantification of coronary artery calcium using ultrafast CT. J Am Coll Cardiol. 1990;15(4):827–32.

32. Budoff MJ, Shaw LJ, Liu ST, Weinstein SR, Tseng PH, Flores FR, et al. Long-term prognosis and coronary calcification. J Am Coll Cardiol. 2007;49(18):1860–70.

33. Sandesara PB, Mehta A, O'Neal WT, Kelli HM, Sathiyakumar V, Martin SS, et al. Zero CAC in LDL-C ≥190 mg/dL: MESA. Atherosclerosis. 2020;292:224–9.

34. Bruker. Lipoprotein subclass analysis. https://www.bruker.com/en/products-and-solutions/magnetic-resonance.html

35. Aru V, Lam C, Khakimov B, Hoefsloot HCJ, Zwanenburg G, Lind MV, et al. Quantification of lipoprotein profiles by NMR spectroscopy and multivariate data analysis. TrAC Trends Anal Chem. 2017;94:210–9.

36. Sommersten CH, Gjerde ES, Laupsa-Borge J, Andersen AI, Lawrence-Archer L, McCann A, et al. Relationship between Ketones, Ghrelin, and Appetite on Isocaloric Diets with Varying Carbohydrate Quality and Amount: Results from CARBFUNC. J Nutr. 2023;153(2):459–69.

37. Coffman CJ, Edelman D, Woolson RF. To condition or not condition? BMJ Open. 2016;6(12):e013096.

38. Lu K. On efficiency of constrained longitudinal data analysis versus L-ANCOVA. Biometrics. 2010;66(3):891–6.

39. Twisk J, Bosman L, Hoekstra T, Rijnhart J, Welten M, Heymans M. Estimating treatment effects in RCTs. Contemp Clin Trials Commun. 2018;10:80–5.

40. Bender R, Lange S. Adjusting for multiple testing—when and how? J Clin Epidemiol. 2001;54(4):343–9.

41. Twisk J, de Boer M, de Vente W, Heymans M. Multiple imputation not necessary before longitudinal mixed-model analysis. J Clin Epidemiol. 2013;66(9):1022–8.
