# Supplementary material for: Lipid dynamics and cardiovascular risk profiles on carbohydrate- or saturated fat-based diets: secondary analysis of the CARBFUNC trial
Source: eBioMedicine. 2026 Oct 24;133:106524. doi: 10.1016/j.ebiom.2026.106524 (PMC13721003; doi:10.1016/j.ebiom.2026.106524)
Supplement: Supplementary file 2 [file mmc1.docx]

**Supplementary Material**

Lipid dynamics and cardiovascular risk profiles on carbohydrate- or saturated fat-based diets (randomized trial CARBFUNC)

Cathrine Loddervik^1,2^, Johnny Laupsa-Borge^2,5^, Cathrine H. Sommersten^2-4^, Kristin Amundsen^1,2^, Arve Ulvik^5^, Kristine K. Johannessen^2^, Synnøve Halvorsen^2^, Terje H. Larsen^6,7^, Ottar K. Nygård^2,6^, Jutta Dierkes^3^, Gunnar Mellgren^1,2^, Simon N. Dankel^1,2*^

^1^Hormone Laboratory, Department of Medical Biochemistry and Pharmacology, Haukeland University Hospital, Bergen, Norway

^2^ Department of Clinical Science, University of Bergen, Norway

^3^ Centre for Nutrition, Department of Clinical Medicine, University of Bergen, Norway

^4^ Department of Ageing, Health and Care, City of Bergen, Norway

^5^ Bevital AS, Bergen, Norway

^6^ Department of Heart Disease, Haukeland University Hospital, Bergen, Norway

^7^ Department of Biomedicine, University of Bergen, Norway

* Corresponding author: Simon N. Dankel, Department of Clinical Science, University of Bergen, Haukeland University Hospital, 5021 Bergen, Norway, [simon.dankel@uib.no](mailto:simon.dankel@uib.no)

**Contents**

**Supplementary Figure 1.** Relative model-adjusted changes as sympercents from baseline to follow-ups in HOMA-IR and total ketones.

**Supplementary Figure 2.** Relative model-adjusted changes as sympercents from baseline to follow-ups in body weight, waist circumference, and systolic and diastolic BP.

**Supplementary Table 1.** Baseline characteristics of biochemical variables presented as

means (SD).

**Supplementary Table 2.** Self-reported dietary adherence.

**Supplementary Table 3.** Relative within- and between-group changes in anthropometry, blood pressure, HOMA2-IR, and total ketones.

**Supplementary Table 4.** Relative within- and between-group changes in standard lipids.

**Supplementary Table 5.** Relative within- and between-group changes in lipoprotein particles and LDL subfractions.

**Supplementary Table 6.** Relative within- and between-group changes in apolipoprotein B and lipid ratios.

**Supplementary Table 7.** Relative within- and between-group changes in HDL particle composition and distribution.

**Supplementary Table 8.** Summary of participant with CAC score > 0 across diet groups.

**Supplementary Table 9.** Baseline characteristics of participants contributing data at 12 and 24 months.

**Supplementary Table 10.** Comparison of primary ITT and diet adherence adjusted ITT analyses for between-group differences in change scores over time.

**Supplementary Table 11.** Comparison of primary and inverse probability weighted (IPW) analyses for nominal significance.

**Supplementary Table 12.** Post hoc exploratory comparison of C-HCLF and LCHF for key lipid outcomes in primary ITT and diet adherence adjusted ITT.

**Supplementary Figures**

**Supplementary Figure 1.** Relative model-adjusted changes as sympercents from baseline to follow-ups in HOMA2-IR and total ketones. Values were naturally log-transformed and analyzed with constrained linear mixed-effects models adjusted for baseline values, age, and sex. Abbreviations: A-HCLF, acellular high-carbohydrate low-fat; C-HCLF, cellular high-carbohydrate low-fat; HOMA2-IR, Homeostatic Model Assessment of Insulin Resistance 2; LCHF, low-carbohydrate high-fat; s%, sympercents.

**Supplementary Figure 2.** Relative model-adjusted changes as sympercents from baseline to follow-ups in body weight, waist circumference, and systolic and diastolic BP. Values were naturally log-transformed and analyzed with constrained linear mixed-effects models adjusted for baseline values, age, and sex. Abbreviations: A-HCLF, acellular high-carbohydrate low-fat; BP, blood pressure; C-HCLF, cellular high-carbohydrate low-fat; LCHF, low-carbohydrate high-fat; s%, sympercents.

**Supplementary Tables**

**Supplementary Table 1.** Baseline characteristics of biochemical variables presented as

means (SD)^1^

| **Characteristics** | **A-HCLF (n = 67)** | **LCHF (n = 63)** | **C-HCLF (n = 62)** |
| --- | --- | --- | --- |
| **TAG (mmol/L)^2^** | 1.53 (0.81) | 1.39 (0.66) | 1.36 (0.58) |
| **TC (mmol/L)^2^** | 5.12 (0.94) | 5.22 (0.94) | 5.32 (0.83) |
| **LDL-C (mmol/L)^2^** | 3.04 (0.70) | 3.20 (0.79) | 3.31 (0.75) |
| **HDL-C (mmol/L)^2^** | 1.21 (0.25) | 1.24 (0.23) | 1.28 (0.24) |
| **Total LDLs (nmol/L)^2^** | 1350 (350) | 1407 (362) | 1444 (319) |
| **Large LDLs (nmol/L)^2^** | 356 (96.0) | 376 (97.5) | 398 (118) |
| **Medium LDLs (nmol/L)^2^** | 389 (116) | 425 (131) | 440 (126) |
| **Small LDLs (nmol/L)^2^** | 623 (277) | 618 (252) | 619 (193) |
| **ApoB (g/L)^2^** | 0.88 (0.22) | 0.91 (0.22) | 0.93 (0.18) |
| **ApoA1 (g/L)^2^** | 1.32 (0.16) | 1.32 (0.16) | 1.34 (0.19) |
| **Glucose (mmol/L)** | 5.38 (0.62) | 5.33 (0.71) | 5.29 (0.72) |
| **HbA1c (mmol/mol)** | 34.1 (3.22) | 34.0 (3.83) | 35.3 (4.06) |
| **Insulin C-peptide (nmol/L)** | 0.95 (0.35) | 0.86 (0.34) | 0.92 (0.33) |
| **HOMA2-IR (index)** | 2.14 (0.84) | 1.93 (0.82) | 2.07 (0.78) |

^1^ Data are presented as arithmetic means (SDs).

^2^ NMR-derived lipid and lipoprotein variables are presented for participants with available baseline plasma data (A-HCLF, n = 61; LCHF, n = 53; C-HCLF, n = 60).

Abbreviations: A-HCLF, acellular high-carbohydrate low-fat; apoA1, apolipoprotein A1; apoB, apolipoprotein B; C-HCLF, cellular high-carbohydrate low-fat; HbA1c, glycated hemoglobin; HDL-C, high-density lipoprotein cholesterol; HOMA2-IR, Homeostatic Model Assessment of Insulin Resistance 2; LCHF, low-carbohydrate high-fat; LDL-C, low-density lipoprotein cholesterol; LDLs, low-density lipoprotein particles; TAGs, triacylglycerols; TC, total cholesterol.

**Supplementary Table 2.** Self-reported dietary adherence^1^

| **Diet groups** | **3M** | **6M** | **9M** | **12M** | **24M** |
| --- | --- | --- | --- | --- | --- |
| **A-HCLF** | 76 (57, 83) | 75 (59, 81) | 75 (65, 80) | 71 (45, 81) | 40 (20, 50) |
| **LCHF** | 85 (73, 93) | 78 (65, 90) | 70 (56, 83) | 70 (53, 89) | 60 (20, 60) |
| **C-HCLF** | 78 (69, 83) | 65 (53,78) | 63 (50, 78) | 68 (49, 78) | 40 (20, 60) |

^1^ Self-reported dietary adherence was assessed at intervals of 3, 6, 9, 12, and 24 months. Data are presented as medians (quartile 1, quartile 3) and calculated from values reported by the participants using a questionnaire with a rating scale ranging from 0% (indicating no adherence) to 100% (indicating complete adherence) in 20% increments. Abbreviations: A-HCLF, acellular high-carbohydrate low-fat; C-HCLF, cellular high-carbohydrate low-fat; LCHF, low-carbohydrate high-fat; M, months.

**Supplementary Table 3.** Relative within- and between-group changes in anthropometry, blood pressure, HOMA-IR, and total ketones^1^

|  | **Change scores 3M** | | **Change scores 6M** | | **Change scores 9M** | | **Change scores 12M** | | **Change scores 24M** |  |
| --- | --- | --- | --- | --- | --- | --- | --- | --- | --- | --- |
| **Variables** | **Within group^2^** | **Between group^3^** | **Within group^2^** | **Between group^3^** | **Within group^2^** | **Between group^3^** | **Within group^2^** | **Between group^3^** | **Within group^2^** | **Between group^3^** |
| **Body weight** |  |  |  |  |  |  |  |  |  |  |
| A-HCLF | -3.96 (-5.13, -2.77) | | -5.79 (-7.62, -3.92) | | -5.22 (-7.61, -2.77) | | -5.41 (-8.10, -2.64) | | -5.50 (-9.19, -1.66) |  |
| LCHF | -5.60 (-6.54, -4.65) | **-1.71 (-3.25, -0.14)** | -7.65 (-9.13, -6.15) | -1.98 (-4.44, 0.54) | -8.29 (-10.3, -6.28) | -3.24 (-6.42, 0.049) | -7.14 (-9.38, -4.85) | -1.83 (-5.46, 1.93) | -2.92 (-6.19, 0.48) | 2.73 (-2.52, 8.26) |
| C-HCLF | -4.92 (-5.96, -3.86) | -1.00 (-2.62, 0.65) | -6.98 (-8.63, -5.31) | -1.27 (-3.85, 1.38) | -7.07 (-9.20, -4.90) | -1.95 (-5.27, 1.48) | -5.93 (-8.29, -3.51) | -0.55 (-4.29, 3.33) | -1.74 (-5.16, 1.80) | 3.98 (-1.41, 9.65) |
| **WC** | |  |  |  |  |  |  |  |  |  |
| A-HCLF | -2.83 (-4.19, -1.44) | | -5.41 (-7.26, -3.52) | | -3.71 (-6.07, -1.30) | | -4.99 (-7.43, -2.49) | | -5.78 (-9.01, -2.44) |  |
| LCHF | -4.29 (-5.26, -3.31) | -1.50 (-3.21, 0.23) | -7.84 (-9.28, -6.38) | **-2.57 (-5.00, -0.077)** | -7.45 (-9.29, -5.56) | **-3.88 (-6.89, -0.76)** | -6.94 (-8.88, -4.95) | -2.05 (-5.27, 1.28) | -4.47 (-7.18, -1.68) | 1.40 (-3.07, 6.07) |
| C-HCLF | -3.64 (-4.73, -2.54) | -0.84 (-2.62, 0.98) | -6.56 (-8.17, -4.92) | -1.21 (-3.78, 1.42) | -6.01 (-8.02, -3.96) | -2.38 (-5.54, 0.87) | -5.04 (-7.12, -2.92) | -0.057 (-3.41, 3.41) | -4.63 (-7.46, -1.72) | 1.23 (-3.32, 5.98) |
| **Systolic BP** | |  |  |  |  |  |  |  |  |  |
| A-HCLF | -0.34 (-3.01, 2.42) |  | 2.10 (-1.88, 6.23) |  | 2.34 (-1.20, 6.00) |  | 0.34 (-4.07, 4.94) |  | 5.81 (0.78, 11.1) |  |
| LCHF | -1.60 (-3.87, 0.72) | -1.27 (-4.65, 2.23) | -2.24 (-5.42, 1.05) | -4.25 (-8.98, 0.74) | -2.63 (-5.68, 0.51) | **-4.85 (-9.11, -0.40)** | -3.25 (-6.77, 0.41) | -3.57 (-8.96, 2.14) | 2.53 (-1.79, 7.05) | -3.10 (-9.13, 3.34) |
| C-HCLF | -1.71 (-4.22, 0.87) | -1.38 (-4.92, 2.30) | 0.90 (-2.68, 4.62) | -1.17 (-6.25, 4.19) | 2.66 (-0.62, 6.04) | 0.31 (-4.21, 5.05) | -1.07 (-4.67, 2.66) | -1.40 (-6.91, 4.43) | 2.19 (-2.18, 6.76) | -3.42 (-9.47, 3.04) |
| **Diastolic BP** | |  |  |  |  |  |  |  |  |  |
| A-HCLF | -2.12 (-5.13, 0.97) |  | -0.071 (-3.75, 3.74) | | 0.30 (-3.62, 4.38) |  | -0.17 (-4.27, 4.10) | | 2.93 (-1.49, 7.55) |  |
| LCHF | -4.22 (-6.74, -1.63) | -2.14 (-5.98, 1.86) | -3.80 (-6.75, -0.75) | -3.73 (-8.24, 1.00) | -4.84 (-8.17, -1.38) | -5.12 (-10.0, 0.052) | -0.13 (-3.56, 3.42) | 0.042 (-5.22, 5.59) | 7.13 (3.07, 11.3) | 4.07 (-1.79, 10.3) |
| C-HCLF | -4.11 (-6.90, -1.24) | -2.03 (-6.07, 2.18) | -2.66 (-5.92, 0.72) | -2.59 (-7.34, 2.40) | -0.62 (-4.17, 3.07) | -0.91 (-6.08, 4.54) | -0.83 (-4.18, 2.64) | -0.65 (-5.84, 4.82) | 2.98 (-0.91, 7.02) | 0.041 (-5.59, 6.00) |
| **HOMA2-IR** |  |  |  |  |  |  |  |  |  |  |
| A-HCLF | -11.8 (-20.6, -2.07) | | -17.0 (-26.7, -5.96) | | -15.4 (-26.0, -3.23) | | -15.2 (-25.4, -3.51) | |  |  |
| LCHF | -12.4 (-19.8, -4.22) | -0.62 (-13.3, 13.9) | -16.4 (-25.1, -6.62) | 0.73 (-14.6, 18.8) | -22.7 (-31.4, -12.9) | -8.68 (-23.5, 9.01) | -14.7 (-23.7, -4.70) | 0.47 (-15.1, 18.9) |  |  |
| C-HCLF | -11.4 (-19.8, -2.21) | 0.42 (-13.0, 15.9) | -16.7 (-25.0, -7.57) | 0.29 (-14.7, 17.9) | -18.7 (-28.0, -8.07) | -3.90 (-19.6, 14.9) | -21.4 (-29.3, -12.6) | -7.35 (-21.4, 9.29) |  |  |
| **Total ketones** | |  |  |  |  |  |  |  |  |  |
| A-HCLF | 36.8 (0.42, 86.5) |  | -1.06 (-30.5, 40.8) | | -23.2 (-40.7, -0.47) | | 5.39 (-22.2, 42.8) |  |  |  |
| LCHF | 218 (145, 313) | **133 (58.7, 241)** | 97.9 (48.5, 164) | **100.0 (29.5, 209)** | 74.9 (37.9, 122) | **128 (63.0, 218)** | 16.2 (-9.69, 49.5) | 10.2 (-25.0, 62.0) |  |  |
| C-HCLF | 36.4 (2.02, 82.2) | -0.36 (-33.4, 49.1) | 19.4 (-11.3, 60.6) | 20.6 (-22.3, 87.4) | -1.61 (-22.8, 25.3) | 28.1 (-8.60, 79.4) | 22.6 (-4.39, 57.3) | 16.4 (-20.6, 70.6) |  |  |

^1^ Relative within-group change scores from baseline and between-group differences for body weight, waist circumference, blood pressure, HOMA2-IR, and total ketones at 3, 6, 9, 12, and 24 months. Values were natural log-transformed and analysed with constrained linear mixed-effects models adjusted for baseline values, age, sex, and couple-randomisation status, with time and group-by-time interaction included in the models. Bold values indicate statistically significant between-group differences. Abbreviations: A-HCLF, acellular high-carbohydrate low-fat; BP, blood pressure; C-HCLF, cellular high-carbohydrate low-fat; HOMA2-IR, Homeostatic Model Assessment of Insulin Resistance 2; LCHF, low-carbohydrate high-fat; M, months; WC, weight circumference.

^2^ Relative model-adjusted mean change scores (95% CIs) within groups from baseline to follow-ups as percentages calculated from the model estimates of natural log-transformed values: % = (exp^estimate^ −1) × 100.

^3^ Estimates (95% CIs) for model-adjusted between-group differences in relative changes from baseline to follow-ups (the A-HCLF intervention as comparator).

**Supplementary Table 4.** Relative within- and between-group changes in standard lipids^1^

|  | **Change scores 3M** |  | **Change scores 6M** |  | **Change scores 9M** |  | **Change scores 12M** | | **Change scores 24M** | |
| --- | --- | --- | --- | --- | --- | --- | --- | --- | --- | --- |
| **Variables** | **Within group^2^** | **Between group^3^** | **Within group^2^** | **Between group^3^** | **Within group^2^** | **Between group^3^** | **Within group^2^** | **Between group^3^** | **Within group^2^** | **Between group^3^** |
| **TC** |  |  |  |  |  |  |  |  |  |  |
| A-HCLF | -4.41 (-7.87, -0.83) |  | -7.48 (-11.9, -2.84) |  | -5.62 (-9.99, -1.04) |  | -2.47 (-7.03, 2.32) | | -2.41 (-8.91, 4.56) | |
| LCHF | 0.12 (-3.01, 3.35) | 4.74 (-0.18, 9.90) | 0.55 (-3.58, 4.86) | **8.68 (2.00, 15.8)** | 5.03 (0.53, 9.73) | **11.3 (4.38, 18.6)** | 2.89 (-1.34, 7.30) | 5.50 (-0.98, 12.4) | 6.30 (-0.16, 13.2) | 8.93 (-0.74, 19.5) |
| C-HCLF | -8.74 (-11.9, -5.51) | -4.52 (-9.19, 0.39) | -7.39 (-11.3, -3.28) | 0.090 (-6.17, 6.77) | -4.40 (-8.51, -0.11) | 1.29 (-5.01, 8.00) | -4.02 (-7.76, -0.13) | -1.59 (-7.51, 4.70) | 3.14 (-3.35, 10.1) | 5.69 (-3.85, 16.2) |
| **LDL-C** |  |  |  |  |  |  |  |  |  |  |
| A-HCLF | -1.75 (-7.05, 3.84) |  | -8.12 (-14.6, -1.21) |  | -8.51 (-14.8, -1.72) |  | -0.11 (-7.44, 7.80) | | -2.97 (-12.5, 7.61) | |
| LCHF | 6.70 (1.72, 11.9) | **8.60 (1.03, 16.8)** | 5.13 (-1.22, 11.9) | **14.4 (4.10, 25.8)** | 12.8 (5.54, 20.6) | **23.3 (12.0, 35.8)** | 7.74 (0.83, 15.1) | 7.86 (-2.44, 19.2) | 9.97 (0.11, 20.8) | 13.3 (-1.42, 30.3) |
| C-HCLF | -8.92 (-13.6, -4.03) | **-7.30 (-14.0, -0.030)** | -7.98 (-13.7, -1.88) | 0.16 (-9.01, 10.2) | -3.19 (-9.43, 3.49) | 5.82 (-3.93, 16.6) | -4.15 (-10.0, 2.09) | -4.05 (-13.0, 5.86) | 1.74 (-7.66, 12.1) | 4.85 (-8.98, 20.8) |
| **HDL-C** |  |  |  |  |  |  |  |  |  |  |
| A-HCLF | -4.75 (-7.74, -1.65) |  | -3.93 (-7.77, 0.066) | | 4.79 (0.52, 9.24) |  | 5.61 (2.50, 8.81) |  | 6.48 (-0.23, 13.6) | |
| LCHF | 4.72 (1.07, 8.51) | **9.94 (4.85, 15.3)** | 12.2 (7.23, 17.3) | **16.8 (9.89, 24.1)** | 18.2 (12.3, 24.4) | **12.8 (5.58, 20.4)** | 13.5 (8.58, 18.7) | **7.50 (1.88, 13.4)** | 10.9 (3.07, 19.3) | 4.13 (-5.55, 14.8) |
| C-HCLF | -5.21 (-9.30, -0.94) | -0.49 (-5.73, 5.05) | -1.32 (-6.36, 3.98) | 2.71 (-3.87, 9.76) | -1.82 (-7.32, 4.00) | -6.31 (-12.7, 0.57) | 4.65 (-0.56, 10.1) | -0.91 (-6.61, 5.14) | 15.4 (6.23, 25.5) | 8.43 (-2.43, 20.5) |
| **Non-HDL-C** |  |  |  |  |  |  |  |  |  |  |
| A-HCLF | -4.00 (-8.49, 0.70) |  | -8.55 (-14.2, -2.57) |  | -9.38 (-15.6, -2.75) |  | -5.32 (-10.9, 0.67) | | -4.52 (-13.8, 5.80) | |
| LCHF | -1.57 (-5.55, 2.57) | 2.53 (-3.67, 9.13) | -3.60 (-8.71, 1.81) | 5.42 (-2.94, 14.5) | 1.51 (-4.84, 8.28) | **12.0 (1.89, 23.1)** | -0.26 (-5.47, 5.24) | 5.34 (-2.85, 14.2) | 3.25 (-5.96, 13.4) | 8.13 (-5.86, 24.2) |
| C-HCLF | -9.88 (-13.8, -5.72) | -6.12 (-12.0, 0.19) | -9.49 (-14.4, -4.25) | -1.02 (-8.99, 7.64) | -5.24 (-11.3, 1.19) | 4.57 (-4.96, 15.1) | -6.56 (-11.2, -1.64) | -1.31 (-8.85, 6.85) | -2.87 (-11.8, 6.93) | 1.72 (-11.6, 17.1) |
| **Remnant-C** |  |  |  |  |  |  |  |  |  |  |
| A-HCLF | -9.00 (-21.3, 5.20) |  | -13.6 (-29.3, 5.50) |  | -12.3 (-27.8, 6.59) |  | -11.0 (-25.5, 6.34) | | 3.01 (-7.54, 14.8) | |
| LCHF | -38.7 (-48.3, -27.3) | **-32.6 (-46.1, -15.9)** | -46.1 (-56.6, -33.0) | **-37.6 (-53.3, -16.5)** | -44.0 (-55.7, -29.1) | **-36.1 (-52.6, -13.9)** | -31.0 (-45.1, -13.4) | -22.5 (-41.6, 2.89) | -23.3 (-41.2, -0.12) | **-25.6 (-44.0, -1.15)** |
| C-HCLF | -8.15 (-23.6, 10.5) | 0.92 (-20.1, 27.4) | -14.7 (-31.9, 6.88) | -1.25 (-26.6, 32.9) | -14.8 (-32.8, 8.09) | -2.78 (-28.1, 31.4) | -17.9 (-33.9, 2.05) | -7.78 (-30.0, 21.5) | -22.5 (-40.5, 0.84) | **-24.8 (-43.4, -0.18)** |
| **TAG** |  |  |  |  |  |  |  |  |  |  |
| A-HCLF | -5.88 (-15.4, 4.77) |  | -8.96 (-21.1, 5.09) |  | -12.3 (-25.9, 3.73) |  | -13.1 (-32.5, 11.8) | | 3.06 (-9.60, 17.5) | |
| LCHF | -26.3 (-33.4, -18.5) | **-21.7 (-32.1, -9.64)** | -32.7 (-41.0, -23.3) | **-26.1 (-39.0, -10.6)** | -35.7 (-45.7, -23.7) | **-26.6 (-42.0, -7.14)** | -20.9 (-31.5, -8.74) | -8.95 (-31.7, 21.4) | -20.4 (-35.6, -1.59) | **-22.8 (-39.5, -1.62)** |
| C-HCLF | -7.69 (-18.8, 4.97) | -1.94 (-16.9, 15.7) | -13.0 (-23.7, -0.70) | -4.43 (-21.1, 15.8) | -8.67 (-21.5, 6.26) | 4.17 (-16.7, 30.3) | -12.4 (-25.2, 2.66) | 0.85 (-25.0, 35.6) | -11.8 (-27.8, 7.58) | -14.5 (-32.2, 7.95) |

^1^ Relative within-group change scores from baseline and between-group differences for TC, LDL-C, HDL-C, remnant-C, and TAG at 3, 6, 9, 12, and 24 months. Fasting plasma levels of standard lipids were measured with nuclear magnetic resonance (NMR) spectroscopy. Values were natural log-transformed and analysed with constrained linear mixed-effects models adjusted for baseline values, age, sex, and couple-randomisation status, with time and group-by-time interaction included in the models. Bold values indicate statistically significant between-group differences. Abbreviations: A-HCLF, acellular high-carbohydrate low-fat; C-HCLF, cellular high-carbohydrate low-fat; HDL-C, high-density lipoprotein cholesterol; LCHF, low-carbohydrate high-fat; LDL-C, low-density lipoprotein cholesterol; M, months; remnant-C, remnant cholesterol; TAGs, triacylglycerols; TC, total cholesterol.

^2^ Relative model-adjusted mean change scores (95% CIs) within groups from baseline to follow-ups as percentages calculated from the model estimates of natural log-transformed values: % = (exp^estimate^ −1) × 100.

^3^ Estimates (95% CIs) for model-adjusted between-group differences in relative changes from baseline to follow-ups (the A-HCLF intervention as comparator).

**Supplementary Table 5.** Relative within- and between-group changes in lipoprotein particles and LDL subfractions^1^

|  | **Change scores 3M** |  | **Change scores 6M** |  | **Change scores 9M** |  | **Change scores 12M** |  | **Change scores 24M** |  |
| --- | --- | --- | --- | --- | --- | --- | --- | --- | --- | --- |
| **Variable** | **Within group^2^** | **Between group^3^** | **Within group^2^** | **Between group^3^** | **Within group^2^** | **Between group^3^** | **Within group^2^** | **Between group^3^** | **Within group^2^** | **Between group^3^** |
| **Total VLDLs** |  |  |  |  |  |  |  |  |  |  |
| A-HCLF | -1.36 (-10.6, 8.81) |  | -6.92 (-17.7, 5.27) |  | -10.1 (-22.2, 3.93) |  | -2.04 (-13.5, 11.0) |  | 8.41 (0.48, 17.0) |  |
| LCHF | -23.1 (-31.1, -14.2) | **-22.0 (-32.6, -9.86)** | -29.2 (-38.2, -18.9) | **-24.0 (-36.6, -8.96)** | -30.1 (-40.7, -17.6) | **-22.3 (-37.3, -3.57)** | -18.4 (-30.3, -4.60) | -16.7 (-31.6, 1.37) | -15.4 (-29.5, 1.62) | -**22.0 (-35.9, -5.04)** |
| C-HCLF | 0.46 (-10.6, 12.9) | 1.82 (-12.4, 18.4) | -8.18 (-20.1, 5.46) | -1.40 (-17.9, 18.4) | -7.79 (-21.8, 8.76) | 2.44 (-17.5, 27.2) | -11.2 (-23.5, 3.13) | -9.33 (-25.1, 9.73) | -18.5 (-31.7, -2.78) | **-24.7 (-37.8, -8.94)** |
| **Total IDLs** |  |  |  |  |  |  |  |  |  |  |
| A-HCLF | -0.36 (-8.70, 8.73) |  | -14.8 (-24.1, -4.33) |  | -17.6 (-28.7, -4.79) |  | -8.14 (-18.1, 2.98) |  | -10.9 (-26.0, 7.38) |  |
| LCHF | -7.22 (-14.0, 0.039) | -6.89 (-16.9, 4.32) | -15.1 (-23.2, -6.09) | -0.36 (-14.4, 16.0) | -10.2 (-21.2, 2.36) | 8.97 (-10.2, 32.2) | -14.0 (-22.1, -4.99) | -6.36 (-19.5, 8.85) | -5.24 (-20.3, 12.6) | 6.33 (-17.5, 37.0) |
| C-HCLF | -10.2 (-17.3, -2.37) | -9.83 (-20.0, 1.56) | -14.9 (-23.2, -5.78) | -0.22 (-14.4, 16.3) | -13.2 (-24.1, -0.68) | 5.41 (-13.3, 28.1) | -16.4 (-23.9, -8.18) | -8.98 (-21.4, 5.42) | -9.77 (-24.4, 7.63) | 1.25 (-21.6, 30.8) |
| **Total LDLs** |  |  |  |  |  |  |  |  |  |  |
| A-HCLF | -2.38 (-7.55, 3.07) |  | -7.12 (-13.0, -0.83) |  | -9.17 (-15.5, -2.34) |  | -0.81 (-7.68, 6.56) |  | -3.71 (-12.7, 6.23) |  |
| LCHF | 2.94 (-1.77, 7.88) | 5.46 (-1.78, 13.2) | 1.57 (-4.02, 7.48) | **9.36 (0.37, 19.1)** | 6.19 (-0.69, 13.5) | **16.9 (6.05, 28.9)** | 3.70 (-2.55, 10.3) | 4.55 (-4.86, 14.9) | 7.80 (-1.45, 17.9) | 11.9 (-1.96, 27.8) |
| C-HCLF | -7.62 (-12.2, -2.77) | -5.37 (-12.1, 1.90) | -7.86 (-13.0, -2.36) | -0.79 (-9.04, 8.21) | -3.42 (-9.74, 3.35) | 6.33 (-3.61, 17.3) | -4.32 (-9.87, 1.56) | -3.54 (-12.1, 5.84) | -0.78 (-9.55, 8.84) | 3.04 (-9.95, 17.9) |
| **Large LDLs** |  |  |  |  |  |  |  |  |  |  |
| A-HCLF | -3.41 (-9.13, 2.66) |  | -7.85 (-14.7, -0.44) |  | -4.89 (-11.8, 2.62) |  | 0.26 (-10.2, 11.9) |  | -1.81 (-12.3, 9.91) |  |
| LCHF | 14.0 (8.10, 20.2) | **18.0 (9.11, 27.7)** | 13.2 (5.89, 21.1) | **22.9 (11.2, 35.8)** | 24.1 (15.7, 33.0) | **30.4 (18.0, 44.1)** | 11.8 (1.69, 22.9) | 11.5 (-3.24, 28.5) | 17.7 (6.42, 30.1) | **19.9 (3.19, 39.2)** |
| C-HCLF | -7.14 (-12.3, -1.63) | -3.86 (-11.4, 4.34) | -1.94 (-8.49, 5.08) | 6.42 (-3.83, 17.8) | 0.43 (-6.39, 7.74) | 5.59 (-4.53, 16.8) | -0.67 (-9.43, 8.93) | -0.92 (-13.9, 14.0) | 8.53 (-2.37, 20.6) | 10.5 (-5.19, 28.8) |
| **Medium LDLs** |  |  |  |  |  |  |  |  |  |  |
| A-HCLF | 2.15 (-5.79, 10.8) |  | -5.45 (-14.8, 5.00) |  | -11.9 (-20.9, -1.99) |  | -0.50 (-12.3, 12.9) |  | -6.58 (-21.6, 11.4) |  |
| LCHF | 14.1 (6.38, 22.4) | **11.7 (0.60, 24.0)** | 5.76 (-3.36, 15.7) | 11.8 (-2.42, 28.2) | 14.5 (3.63, 26.4) | **30.0 (12.6, 50.1)** | 10.3 (-1.11, 23.0) | 10.8 (-5.93, 30.6) | 1.56 (-13.4, 19.0) | 8.71 (-14.1, 37.6) |
| C-HCLF | -5.22 (-12.2, 2.33) | -7.21 (-16.8, 3.51) | -6.65 (-14.9, 2.39) | -1.28 (-14.0, 13.4) | -1.37 (-10.8, 9.01) | 12.0 (-3.06, 29.4) | -4.49 (-14.0, 6.08) | -4.00 (-18.3, 12.8) | -2.98 (-17.6, 14.3) | 3.85 (-18.3, 31.9) |
| **Small LDLs** |  |  |  |  |  |  |  |  |  |  |
| A-HCLF | -4.27 (-13.4, 5.78) |  | -6.71 (-15.4, 2.83) |  | -12.7 (-23.6, -0.31) |  | -0.90 (-11.0, 10.3) |  | 1.13 (-14.8, 20.1) |  |
| LCHF | -10.2 (-17.6, -2.19) | -6.20 (-17.5, 6.68) | -7.47 (-14.9, 0.64) | -0.81 (-12.4, 12.3) | -6.75 (-17.4, 5.24) | 6.83 (-10.3, 27.2) | -3.74 (-12.3, 5.62) | -2.86 (-15.5, 11.6) | -2.39 (-16.7, 14.4) | -3.48 (-23.5, 21.8) |
| C-HCLF | -5.51 (-13.9, 3.75) | -1.29 (-13.7, 12.9) | -9.09 (-16.7, -0.83) | -2.55 (-14.1, 10.6) | -5.01 (-16.1, 7.57) | 8.82 (-8.88, 30.0) | -3.98 (-12.3, 5.16) | -3.10 (-15.6, 11.3) | -8.48 (-22.2, 7.66) | -9.50 (-28.5, 14.5) |

^1^ Relative within-group change scores from baseline and between-group differences for total VLDLs, IDLs, and LDLs, and for major LDL subfractions at 3, 6, 9, 12, and 24 months. Fasting plasma levels of lipoprotein particles and subfractions were measured with nuclear magnetic resonance (NMR) spectroscopy. Values were natural log-transformed and analysed with constrained linear mixed-effects models adjusted for baseline values, age, sex, and couple-randomisation status, with time and group-by-time interaction included in the models. Bold values indicate statistically significant between-group differences. Abbreviations: A-HCLF, acellular high-carbohydrate low-fat; C-HCLF, cellular high-carbohydrate low-fat; IDLs, intermediate-density lipoprotein particles; LCHF, low-carbohydrate high-fat; LDLs, low-density lipoprotein particles; M, months; VLDLs, very-low-density lipoprotein particles.

^2^ Relative model-adjusted mean change scores (95% CIs) within groups from baseline to follow-ups as percentages calculated from the model estimates of natural log-transformed values: % = (exp^estimate^ −1) × 100.

^3^ Estimates (95% CIs) for model-adjusted between-group differences in relative changes from baseline to follow-ups (the A-HCLF intervention as comparator).

**Supplementary Table 6.** Relative within- and between-group changes in apolipoprotein B and lipid ratios^1^

|  | **Change scores 3M** |  | **Change scores 6M** |  | **Change scores 9M** |  | **Change scores 12M** |  | **Change scores 24M** |  |
| --- | --- | --- | --- | --- | --- | --- | --- | --- | --- | --- |
| **Variables** | **Within group^2^** | **Between group^3^** | **Within group^2^** | **Between group^3^** | **Within group^2^** | **Between group^3^** | **Within group^2^** | **Between group^3^** | **Within group^2^** | **Between group^3^** |
| **ApoB** |  |  |  |  |  |  |  |  |  |  |
| A-HCLF | -1.04 (-5.66, 3.81) |  | -6.46 (-11.8, -0.83) |  | -9.00 (-15.3, -2.25) |  | -1.17 (-7.12, 5.17) |  | -1.47 (-10.6, 8.57) |  |
| LCHF | 0.71 (-3.36, 4.94) | 1.76 (-4.38, 8.30) | -1.43 (-6.28, 3.67) | 5.37 (-2.37, 13.7) | 2.57 (-3.91, 9.48) | **12.7 (2.42, 24.0)** | 1.15 (-4.15, 6.75) | 2.35 (-5.69, 11.1) | 4.98 (-3.92, 14.7) | 6.55 (-6.56, 21.5) |
| C-HCLF | -6.86 (-11.0, -2.58) | -5.88 (-11.8, 0.43) | -7.86 (-12.5, -2.97) | -1.50 (-8.83, 6.42) | -3.66 (-9.88, 2.98) | 5.86 (-3.90, 16.6) | -4.93 (-9.76, 0.15) | -3.81 (-11.3, 4.27) | -1.68 (-10.2, 7.70) | -0.21 (-12.6, 14.0) |
| **LDL-C/apoB** |  |  |  |  |  |  |  |  |  |  |
| A-HCLF | -0.19 (-3.23, 2.94) |  | -1.25 (-4.77, 2.40) |  | 0.78 (-2.65, 4.33) |  | 1.26 (-3.02, 5.73) |  | 0.41 (-3.59, 4.57) |  |
| LCHF | 5.71 (2.95, 8.54) | **5.91 (1.79, 10.2)** | 6.49 (3.25, 9.84) | **7.84 (2.89, 13.0)** | 11.1 (7.61, 14.6) | **10.2 (5.32, 15.3)** | 7.94 (3.99, 12.0) | **6.59 (0.86, 12.6)** | 4.00 (0.24, 7.90) | 3.58 (-1.85, 9.30) |
| C-HCLF | -1.64 (-4.44, 1.25) | -1.45 (-5.44, 2.72) | -0.61 (-3.77, 2.66) | 0.65 (-4.07, 5.61) | 0.82 (-2.38, 4.12) | 0.040 (-4.45, 4.74) | 1.65 (-1.92, 5.36) | 0.39 (-4.92, 5.99) | 2.67 (-1.15, 6.65) | 2.26 (-3.18, 8.01) |
| **Non-HDL-C/apoB** |  |  |  |  |  |  |  |  |  |  |
| A-HCLF | -2.91 (-4.40, -1.39) |  | -1.83 (-3.75, 0.12) |  | -0.55 (-2.20, 1.13) |  | -4.09 (-5.94, -2.19) |  | -2.59 (-4.43, -0.71) |  |
| LCHF | -2.18 (-3.48, -0.86) | 0.75 (-1.24, 2.78) | -2.06 (-3.68, -0.42) | -0.24 (-2.74, 2.33) | -0.49 (-2.06, 1.10) | 0.061 (-2.18, 2.35) | -0.14 (-1.80, 1.55) | **4.12 (1.53, 6.78)** | -0.86 (-2.64, 0.95) | 1.77 (-0.86, 4.48) |
| C-HCLF | -3.31 (-4.73, -1.88) | -0.42 (-2.48, 1.69) | -2.34 (-4.03, -0.62) | -0.52 (-3.07, 2.10) | -1.62 (-3.18, -0.038) | -1.08 (-3.30, 1.20) | -1.61 (-3.16, -0.038) | **2.58 (0.083, 5.14)** | -0.94 (-2.71, 0.88) | 1.70 (-0.94, 4.40) |
| **TAG/HDL-C** |  |  |  |  |  |  |  |  |  |  |
| A-HCLF | 0.44 (-9.89, 12.0) |  | -4.81 (-19.1, 12.0) |  | -15.4 (-30.3, 2.64) |  | -19.6 (-38.0, 4.21) |  | -7.05 (-19.1, 6.78) |  |
| LCHF | -30.1 (-38.2, -21.0) | **-30.4 (-40.7, -18.4)** | -41.1 (-49.5, -31.4) | **-38.2 (-50.3, -23.1)** | -45.6 (-55.3, -33.9) | **-35.7 (-51.0, -15.8)** | -30.7 (-41.6, -17.9) | -13.8 (-36.7, 17.3) | -28.2 (-41.8, -11.4) | **-22.7 (-39.8, -0.78)** |
| C-HCLF | -2.78 (-15.4, 11.8) | -2.68 (-18.3, 15.9) | -12.3 (-24.4, 1.68) | -7.99 (-25.8, 14.1) | -8.41 (-23.7, 9.89) | 8.39 (-16.6, 40.8) | -16.8 (-31.1, 0.46) | 3.47 (-24.7, 42.2) | -22.7 (-42.7, 4.39) | -17.4 (-40.5, 14.6) |

^1^ Relative within-group change scores from baseline and between-group differences for apoB, LDL-C/apoB, Non-HDL-C/apoB, and TAG/HDL-C at 3, 6, 9, 12, and 24 months. Fasting plasma levels of apoB, LDL-C, and HDL-C were measured with nuclear magnetic resonance (NMR) spectroscopy. Values were natural log-transformed and analysed with constrained linear mixed-effects models adjusted for baseline values, age, sex, and couple-randomisation status, with time and group-by-time interaction included in the models. Bold values indicate statistically significant between-group differences. Abbreviations: apoB, apolipoprotein B; A-HCLF, acellular high-carbohydrate low-fat; C-HCLF, cellular high-carbohydrate low-fat; LDL-C, low-density lipoprotein cholesterol; LCHF, low-carbohydrate high-fat; HDL-C, high-density lipoprotein cholesterol; M, months; TAG, triacylglycerol.

^2^ Relative model-adjusted mean change scores (95% CIs) within groups from baseline to follow-ups as percentages calculated from the model estimates of natural log transformed values: % = (exp^estimate^ −1) × 100.

^3^ Estimates (95% CIs) for model-adjusted between-group differences in relative changes from baseline to follow-ups (the A-HCLF intervention as comparator).

**Supplementary Table 7.** Relative within- and between-group changes in HDL particle composition and distribution^1^

|  | **Change scores 3M** |  | **Change scores 6M** |  | **Change scores 9M** |  | **Change scores 12M** |  | **Change scores 24M** |  |
| --- | --- | --- | --- | --- | --- | --- | --- | --- | --- | --- |
| **Variable** | **Within group^2^** | **Between group^3^** | **Within group^2^** | **Between group^3^** | **Within group^2^** | **Between group^3^** | **Within group^2^** | **Between group^3^** | **Within group^2^** | **Between group^3^** |
| **ApoA1** |  |  |  |  |  |  |  |  |  |  |
| A-HCLF | -4.28 (-6.98, -1.50) |  | -2.87 (-6.53, 0.92) |  | 2.71 (-1.01, 6.57) |  | 1.96 (-1.67, 5.73) |  | 3.97 (-1.67, 9.94) |  |
| LCHF | 0.63 (-1.81, 3.13) | **5.13 (1.29, 9.11)** | 4.35 (1.00, 7.80) | **7.43 (2.19, 13.0)** | 6.95 (3.29, 10.7) | 4.13 (-0.99, 9.52) | 7.61 (4.27, 11.1) | **5.54 (0.60, 10.7)** | 4.11 (-1.07, 9.55) | 0.13 (-7.13, 7.95) |
| C-HCLF | -4.87 (-7.39, -2.28) | -0.62 (-4.39, 3.32) | -1.95 (-5.22, 1.44) | 0.96 (-4.07, 6.24) | -2.37 (-5.70, 1.09) | **-4.94 (-9.61, -0.019)** | 1.98 (-1.05, 5.09) | 0.013 (-4.58, 4.83) | 11.6 (5.85, 17.6) | 7.32 (-0.58, 15.8) |
| **ApoA2** |  |  |  |  |  |  |  |  |  |  |
| A-HCLF | -6.63 (-9.44, -3.72) |  | -5.39 (-9.57, -1.02) |  | 0.056 (-3.47, 3.71) |  | -1.02 (-4.27, 2.34) |  | -0.88 (-5.82, 4.33) |  |
| LCHF | -7.63 (-9.88, -5.34) | -1.08 (-4.79, 2.77) | -2.43 (-5.89, 1.16) | 3.13 (-2.59, 9.19) | 1.67 (-1.36, 4.80) | 1.61 (-3.01, 6.46) | 2.84 (0.26, 5.50) | 3.91 (-0.31, 8.30) | 4.60 (0.14, 9.27) | 5.53 (-1.00, 12.5) |
| C-HCLF | -7.04 (-10.4, -3.55) | -0.45 (-5.00, 4.33) | -3.99 (-8.50, 0.75) | 1.49 (-4.92, 8.32) | -5.75 (-9.95, -1.34) | **-5.80 (-11.1, -0.21)** | 0.21 (-3.79, 4.37) | 1.24 (-3.88, 6.64) | 5.39 (-1.03, 12.2) | 6.32 (-1.67, 15.0) |
| **ApoA1 in large HDLs** |  |  |  |  |  |  |  |  |  |  |
| A-HCLF | -1.00 (-6.69, 5.04) |  | 4.03 (-3.67, 12.4) |  | 13.9 (0.54, 29.1) |  | 14.8 (3.62, 27.3) |  | 19.0 (11.6, 26.8) |  |
| LCHF | 19.7 (11.1, 29.1) | **20.9 (10.1, 32.9)** | 28.2 (16.9, 40.5) | **23.2 (9.32, 38.9)** | 29.5 (16.9, 43.5) | 13.7 (-3.19, 33.5) | 20.9 (10.7, 32.0) | 5.27 (-8.07, 20.5) | 9.96 (-0.68, 21.7) | -7.60 (-18.1, 4.22) |
| C-HCLF | -0.74 (-7.24, 6.22) | 0.26 (-8.23, 9.54) | 3.49 (-4.19, 11.8) | -0.52 (-10.8, 10.9) | 3.41 (-6.50, 14.4) | -9.24 (-22.6, 6.47) | 10.0 (0.81, 20.1) | -4.18 (-16.3, 9.68) | 27.6 (-2.31, 66.7) | 7.25 (-18.5, 41.2) |
| **ApoA1 in small HDLs** |  |  |  |  |  |  |  |  |  |  |
| A-HCLF | -5.85 (-9.03, -2.57) |  | -4.98 (-9.38, -0.37) |  | 0.090 (-3.90, 4.24) |  | -1.10 (-4.91, 2.86) |  | 1.13 (-3.48, 5.96) |  |
| LCHF | -6.20 (-8.90, -3.42) | -0.37 (-4.69, 4.15) | -1.43 (-5.27, 2.56) | 3.74 (-2.38, 10.2) | 1.15 (-2.60, 5.04) | 1.06 (-4.35, 6.77) | 5.37 (1.84, 9.03) | **6.55 (1.29, 12.1)** | 3.13 (-1.18, 7.63) | 1.99 (-4.12, 8.47) |
| C-HCLF | -5.75 (-8.73, -2.68) | 0.11 (-4.43, 4.86) | -1.49 (-5.58, 2.78) | 3.68 (-2.62, 10.4) | -3.24 (-6.84, 0.49) | -3.33 (-8.52, 2.15) | 2.28 (-1.06, 5.73) | 3.42 (-1.64, 8.74) | 4.92 (0.42, 9.62) | 3.75 (-2.54, 10.4) |
| **ApoA2 in large HDLs** |  |  |  |  |  |  |  |  |  |  |
| A-HCLF | -11.0 (-18.9, -2.36) |  | -1.46 (-12.5, 10.9) |  | 3.88 (-8.10, 17.4) |  | 5.31 (-6.17, 18.2) |  | 3.38 (-13.8, 24.0) |  |
| LCHF | -0.46 (-8.17, 7.90) | 11.8 (-0.43, 25.6) | 7.07 (-3.29, 18.5) | 8.66 (-6.83, 26.7) | 7.63 (-3.89, 20.5) | 3.61 (-11.9, 21.9) | 5.93 (-4.16, 17.1) | 0.59 (-13.4, 16.8) | 7.03 (-9.12, 26.1) | 3.53 (-18.8, 32.0) |
| C-HCLF | -5.15 (-13.1, 3.51) | 6.58 (-5.61, 20.3) | -5.04 (-14.5, 5.48) | -3.63 (-17.6, 12.7) | -1.96 (-12.6, 9.97) | -5.62 (-19.9, 11.2) | -1.01 (-10.2, 9.08) | -5.99 (-18.9, 9.00) | 24.6 (5.17, 47.7) | 20.5 (-5.87, 54.4) |
| **ApoA2 in small HDLs** |  |  |  |  |  |  |  |  |  |  |
| A-HCLF | -7.53 (-10.4, -4.62) |  | -7.31 (-12.4, -1.97) |  | -2.50 (-6.18, 1.33) |  | -4.53 (-9.49, 0.70) |  | -4.45 (-12.0, 3.75) |  |
| LCHF | -12.0 (-15.2, -8.72) | **-4.84 (-9.22, -0.24)** | -6.53 (-10.7, -2.20) | 0.84 (-6.11, 8.31) | -1.93 (-7.14, 3.57) | 0.58 (-5.90, 7.50) | 0.83 (-3.05, 4.87) | 5.62 (-1.10, 12.8) | 2.57 (-1.99, 7.35) | 7.35 (-2.17, 17.8) |
| C-HCLF | -7.78 (-11.4, -4.00) | -0.26 (-5.12, 4.85) | -5.04 (-9.46, -0.41) | 2.45 (-4.76, 10.2) | -5.84 (-10.2, -1.29) | -3.43 (-9.11, 2.60) | 0.40 (-3.78, 4.75) | 5.16 (-1.72, 12.5) | 0.32 (-9.70, 11.4) | 4.99 (-8.05, 19.9) |

^1^ Relative within-group change scores from baseline and between-group differences for apoA1, apoA2, large and small HDL apoA1, and large and small HDL apoA2 at 3, 6, 9, 12, and 24 months. Fasting plasma levels of total apoA1 and apoA2 and levels within different HDL subclasses were measured with nuclear magnetic resonance (NMR)spectroscopy. Values were natural log-transformed and analysed with constrained linear mixed-effects models adjusted for baseline values, age, sex, and couple-randomisation status, with time and group-by-time interaction included in the models. Bold values indicate statistically significant between-group differences. Abbreviations: A-HCLF, acellular high-carbohydrate low-fat; apoA1, apolipoprotein A1; apoA2, apolipoprotein A2; C-HCLF, cellular high-carbohydrate low-fat; HDLs, high-density lipoprotein particles; LCHF, low-carbohydrate high-fat; M, months.

^2^ Relative model-adjusted mean change scores (95% CIs) within groups from baseline to follow-ups as percentages calculated from the model estimates of natural log transformed values: % = (exp^estimate^ −1) × 100.

^3^ Estimates (95% CIs) for model-adjusted between-group differences in relative changes from baseline to follow-ups (the A-HCLF intervention as comparator).

**Supplementary Table 8.** Summary of participant with CAC score > 0 across diet groups^1^

| **A-HCLF** |  |  | **LCHF** |  |  | **C-HCLF** |  |  |
| --- | --- | --- | --- | --- | --- | --- | --- | --- |
| **0M** | **6M** | **12M** | **0M** | **6M** | **12M** | **0M** | **6M** | **12M** |
| 10 | 32 | 13 | 59 | 60 | 61 | 3 |  |  |
| 0 | 1 |  | 9 |  |  | 169 | 200 | 237 |
| 54 |  |  | 39 |  |  | 2 | 1 | 2 |
| 2 |  |  | 23 | 27 | 35 | 98 | 101 | 113 |
| 35 | 46 |  | 0 | 0 | 1 | 0 | 3 | 0 |
| 53 | 53 | 66 | 17 | 16 | 17 | 16 | 28 | 24 |
| 6 | 11 | 11 | 104 |  |  | 2 | 0 | 1 |
| 60 | 68 | 71 | 11 | 12 | 17 | 7 |  |  |
|  |  |  | 14 |  |  | 1961 |  |  |
|  |  |  | 40 | 46 |  | 1 |  |  |
|  |  |  | 69 | 93 | 105 | 47 | 87 |  |
|  |  |  |  |  |  | 2 |  |  |
|  |  |  |  |  |  | 0 | 3 | 11 |

^1^ Table displays all participants with available measures and CAC score greater than 0 at baseline, 6 months, or 12 months during the intervention. Abbreviations: A-HCLF, acellular high-carbohydrate low-fat; C-HCLF, cellular high-carbohydrate low-fat; LCHF, low-carbohydrate high-fat; M, months.

**Supplementary Table 9.** Baseline characteristics of participants contributing data at 12 and 24 months^1^

|  | **Participants with 12-month data** | |  | **Participants with 24-month data** | |  |
| --- | --- | --- | --- | --- | --- | --- |
| **Characteristics** | **A-HCLF (n = 14)** | **LCHF (n = 17)** | **C-HCLF (n = 21)** | **A-HCLF (n = 9)** | **LCHF (n = 11)** | **C-HCLF (n = 10)** |
| **Age** | 45.43 (6.16) | 43.10 (9.45) | 42.59 (7.94) | 42.44 (8.09) | 43.55 (8.69) | 45.40 (4.22) |
| **Sex (% females)** | 50 | 30 | 50 | 56 | 27 | 50 |
| **BMI (kg/m)** | 37.22 (5.35) | 34.59 (4.06) | 38.37 (5.71) | 37.04 (4.57) | 35.06 (4.11) | 37.61 (5.16) |
| **Waist circumference (cm)** | 117.96 (11.15) | 114.77 (13.24) | 121.66 (13.10) | 116.87 (13.19) | 117.15 (12.47) | 121.19 (10.14) |
| **TAG (mmol/L)** | 1.53 (0.70) | 1.48 (0.48) | 1.44 (0.71) | 1.45 (0.51) | 1.51 (0.52) | 1.26 (0.34) |
| **TC (mmol/L)** | 4.96 (0.70) | 5.24 (1.03) | 5.45 (0.90) | 4.90 (0.64) | 5.24 (0.93) | 5.24 (0.97) |
| **LDL-C (mmol/L)** | 2.91 (0.45) | 3.13 (0.90) | 3.42 (0.84) | 2.94 (0.51) | 3.16 (0.73) | 3.31 (0.89) |
| **HDL-C (mmol/L)** | 1.24 (0.22) | 1.22 (0.24) | 1.25 (0.20) | 1.24 (0.27) | 1.21 (0.25) | 1.26 (0.25) |
| **Remnant-C (mmol/L)** | 2.53 (1.25) | 2.45 (0.88) | 2.33 (1.27) | 2.37 (1.00) | 2.51 (1.00) | 2.09 (0.68) |
| **ApoB (g/L)** | 0.85 (0.14 | 0.90 (0.22) | 0.96 (0.19) | 0.83 (0.12) | 0.91 (0.22) | 0.91 (0.23) |
| **ApoA1 (g/L )** | 1.34 (0.14) | 1.33 (0.17 | 1.32 (0.13) | 1.32 (0.15) | 1.31 (0.15) | 1.30 (0.13) |

Abbreviations: A-HCLF, acellular high-carbohydrate low-fat; apoA1, apolipoprotein A1; apoB, apolipoprotein B; BMI, body mass index; C-HCLF, cellular high-carbohydrate low-fat; HDL-C, high-density lipoprotein cholesterol; LCHF, low-carbohydrate high-fat; LDL-C, low-density lipoprotein cholesterol; remnant C, remnant cholesterol; TAGs, triacylglycerols; TC, total cholesterol.

^1^ Data are presented as arithmetic means (SDs). Sex is presented as % females.

**Supplementary Table 10.** Comparison of primary ITT and diet adherence adjusted ITT analyses for between-group differences in change scores over time^1^

|  | **Change scores 3M** |  | **Change scores 6M** |  | **Change scores 9M** | | **Change scores 12M** | |
| --- | --- | --- | --- | --- | --- | --- | --- | --- |
| **Variable** | **Primary ITT** | **Diet adherence adjusted ITT** | **Primary ITT** | **Diet adherence adjusted ITT** | **Primary ITT** | **Diet adherence adjusted ITT** | **Primary ITT** | **Diet adherence adjusted ITT** |
| **TC** |  |  |  |  |  |  |  |  |
| LCHF vs. A-HCLF | 4.74 (-0.18, 9.90) | 4.48 (-1.49, 10.8) | 8.68 (2.00, 15.8) | 9.92 (2.02, 18.4) | 11.3 (4.38, 18.6) | 10.4 (2.05, 19.5) | 5.50 (-0.98, 12.4) | 6.52 (-1.82, 15.6) |
| C-HCLF vs. A-HCLF | -4.52 (-9.19, 0.39) | -4.33 (-9.11, 0.70) | 0.090 (-6.17, 6.77) | -1.56 (-7.76, 5.06) | 1.29 (-5.01, 8.00) | -1.22 (-7.68, 5.68) | -1.59 (-7.51, 4.70) | -2.32 (-8.89, 4.73) |
| **LDL-C** |  |  |  |  |  |  |  |  |
| LCHF vs. A-HCLF | 8.60 (1.03, 16.8) | 9.36 (0.052, 19.5) | 14.4 (4.10, 25.8) | 17.5 (4.87, 31.6) | 23.3 (12.0, 35.8) | 22.4 (9.05, 37.4) | 7.86 (-2.44, 19.2) | 11.9 (-0.58, 25.9) |
| C-HCLF vs. A-HCLF | **-7.30 (-14.0, -0.030)** | **-6.82 (-13.5, 0.31)** | 0.16 (-9.01, 10.2) | -0.52 (-10.2, 10.2) | 5.82 (-3.93, 16.6) | 1.22 (-9.63, 13.4) | -4.05 (-13.0, 5.86) | -3.59 (-14.2, 8.28) |
| **HDL-C** |  |  |  |  |  |  |  |  |
| LCHF vs. A-HCLF | 9.94 (4.85, 15.3) | 15.3 (8.91, 22.0) | 16.8 (9.89, 24.1) | 21.4 (12.0, 31.6) | 12.8 (5.58, 20.4) | 22.4 (12.7, 32.9) | 7.50 (1.88, 13.4) | 20.8 (13.3, 28.9) |
| C-HCLF vs. A-HCLF | -0.49 (-5.73, 5.05) | -2.33 (-7.20, 2.80) | 2.71 (-3.87, 9.76) | -1.02 (-7.37, 5.78) | -6.31 (-12.7, 0.57) | -7.56 (-14.9, 0.43) | -0.91 (-6.61, 5.14) | -0.064 (-6.71, 7.06) |
| **Non-HDL-C** |  |  |  |  |  |  |  |  |
| LCHF vs. A-HCLF | 2.53 (-3.67, 9.13) | 1.16 (-6.33, 9.24) | 5.42 (-2.94, 14.5) | 5.61 (-4.26, 16.5) | **12.0 (1.89, 23.1)** | **8.10 (-3.46, 21.1)** | 5.34 (-2.85, 14.2) | 3.58 (-6.75, 15.0) |
| C-HCLF vs. A-HCLF | -6.12 (-12.0, 0.19) | -5.50 (-11.6, 1.02) | -1.02 (-8.99, 7.64) | -2.21 (-10.2, 6.51) | 4.57 (-4.96, 15.1) | 0.99 (-8.56, 11.5) | -1.31 (-8.85, 6.85) | -3.61 (-11.8, 5.36) |
| **Remnant-C** |  |  |  |  |  |  |  |  |
| LCHF vs. A-HCLF | -32.6 (-46.1, -15.9) | -40.2 (-53.6, -23.1) | -37.6 (-53.3, -16.5) | -40.8 (-57.6, -17.3) | -36.1 (-52.6, -13.9) | -49.4 (-64.3, -28.2) | **-22.5 (-41.6, 2.89)** | **-47.9 (-62.1, -28.4)** |
| C-HCLF vs. A-HCLF | 0.92 (-20.1, 27.4) | 0.011 (-20.7, 26.1) | -1.25 (-26.6, 32.9) | -3.61 (-29.4, 31.7) | -2.78 (-28.1, 31.4) | -6.41 (-31.9, 28.5) | -7.78 (-30.0, 21.5) | -18.6 (-38.4, 7.59) |
| **TAG** |  |  |  |  |  |  |  |  |
| LCHF vs. A-HCLF | -21.7 (-32.1, -9.64) | -28.1 (-39.5, -14.6) | -26.1 (-39.0, -10.6) | -31.0 (-45.0, -13.5) | -26.6 (-42.0, -7.14) | -37.2 (-51.4, -19.0) | **-8.95 (-31.7, 21.4)** | **-33.2 (-45.4, -18.2)** |
| C-HCLF vs. A-HCLF | -1.94 (-16.9, 15.7) | -1.99 (-16.0, 14.4) | -4.43 (-21.1, 15.8) | -6.29 (-24.2, 15.9) | 4.17 (-16.7, 30.3) | 1.79 (-18.7, 27.4) | 0.85 (-25.0, 35.6) | -13.6 (-29.3, 5.61) |
| **Total VLDLs** |  |  |  |  |  |  |  |  |
| LCHF vs. A-HCLF | -22.0 (-32.6, -9.86) | -29.1 (-40.0, -16.3) | -24.0 (-36.6, -8.96) | -29.5 (-43.3, -12.3) | -22.3 (-37.3, -3.57) | -34.9 (-49.5, -15.9) | **-16.7 (-31.6, 1.37)** | **-37.9 (-49.7, -23.3)** |
| C-HCLF vs. A-HCLF | 1.82 (-12.4, 18.4) | 1.30 (-12.8, 17.6) | -1.40 (-17.9, 18.4) | -3.10 (-20.7, 18.4) | 2.44 (-17.5, 27.2) | -0.18 (-20.9, 26.0) | -9.33 (-25.1, 9.73) | -15.6 (-29.8, 1.45) |
| **Total IDLs** |  |  |  |  |  |  |  |  |
| LCHF vs. A-HCLF | -6.89 (-16.9, 4.32) | -0.38 (-12.7, 13.7) | -0.36 (-14.4, 16.0) | 3.79 (-13.2, 24.1) | 8.97 (-10.2, 32.2) | 11.3 (-12.0, 40.7) | -6.36 (-19.5, 8.85) | -8.59 (-24.2, 10.2) |
| C-HCLF vs. A-HCLF | -9.83 (-20.0, 1.56) | -9.00 (-19.2, 2.51) | -0.22 (-14.4, 16.3) | -6.40 (-20.9, 10.7) | 5.41 (-13.3, 28.1) | -6.71 (-25.3, 16.5) | **-8.98 (-21.4, 5.42)** | **-17.0 (-29.6, -2.04)** |
| **Total LDLs** |  |  |  |  |  |  |  |  |
| LCHF vs. A-HCLF | 5.46 (-1.78, 13.2) | 3.24 (-5.18, 12.4) | **9.36 (0.37, 19.1)** | **-4.66 (-12.2, 3.55)** | 16.9 (6.05, 28.9) | 13.4 (1.92, 26.1) | 4.55 (-4.86, 14.9) | -0.082 (-10.3, 11.3) |
| C-HCLF vs. A-HCLF | -5.37 (-12.1, 1.90) | -5.46 (-11.8, 1.30) | -0.79 (-9.04, 8.21) | 4.07 (-3.44, 12.2) | 6.33 (-3.61, 17.3) | 4.07 (-6.61, 16.0) | -3.54 (-12.1, 5.84) | -5.83 (-15.0, 4.34) |
| **Large LDLs** |  |  |  |  |  |  |  |  |
| LCHF vs. A-HCLF | 18.0 (9.11, 27.7) | 19.5 (8.41, 31.6) | 22.9 (11.2, 35.8) | 29.1 (14.3, 45.8) | 30.4 (18.0, 44.1) | 30.0 (15.2, 46.8) | 11.5 (-3.24, 28.5) | 15.8 (-3.11, 38.3) |
| C-HCLF vs. A-HCLF | -3.86 (-11.4, 4.34) | -3.70 (-11.5, 4.78) | 6.42 (-3.83, 17.8) | 5.47 (-5.14, 17.3) | 5.59 (-4.53, 16.8) | 1.75 (-8.06, 12.6) | -0.92 (-13.9, 14.0) | -5.02 (-19.4, 11.9) |
| **Medium LDLs** |  |  |  |  |  |  |  |  |
| LCHF vs. A-HCLF | 11.7 (0.60, 24.0) | 14.9 (1.38, 30.3) | **11.8 (-2.42, 28.2)** | **19.8 (1.43, 41.5)** | 30.0 (12.6, 50.1) | 29.4 (8.53, 54.3) | 10.8 (-5.93, 30.6) | 20.9 (-2.83, 50.4) |
| C-HCLF vs. A-HCLF | -7.21 (-16.8, 3.51) | -7.16 (-16.5, 3.27) | -1.28 (-14.0, 13.4) | -1.84 (-15.1, 13.4) | 12.0 (-3.06, 29.4) | 5.30 (-9.32, 22.3) | -4.00 (-18.3, 12.8) | -4.54 (-21.6, 16.2) |
| **Small LDLs** |  |  |  |  |  |  |  |  |
| LCHF vs. A-HCLF | -6.20 (-17.5, 6.68) | -9.51 (-22.3, 5.41) | -0.81 (-12.4, 12.3) | -3.12 (-17.8, 14.2) | 6.83 (-10.3, 27.2) | 8.03 (-12.7, 33.7) | -2.86 (-15.5, 11.6) | -11.1 (-25.9, 6.63) |
| C-HCLF vs. A-HCLF | -1.29 (-13.7, 12.9) | -0.44 (-13.4, 14.4) | -2.55 (-14.1, 10.6) | 0.90 (-12.5, 16.4) | 8.82 (-8.88, 30.0) | 12.0 (-7.54, 35.7) | -3.10 (-15.6, 11.3) | -3.89 (-17.7, 12.3) |
| **ApoA1** |  |  |  |  |  |  |  |  |
| LCHF vs. A-HCLF | 5.13 (1.29, 9.11) | 8.05 (3.61, 12.7) | 7.43 (2.19, 13.0) | 11.2 (5.10, 17.7) | 4.13 (-0.99, 9.52) | 8.10 (1.73, 14.9) | 5.54 (0.60, 10.7) | 10.8 (5.10, 16.7) |
| C-HCLF vs. A-HCLF | -0.62 (-4.39, 3.32) | -1.60 (-5.32, 2.26) | 0.96 (-4.07, 6.24) | -2.01 (-7.15, 3.42) | -4.94 (-9.61, -0.019) | -6.07 (-11.3, -0.53) | 0.013 (-4.58, 4.83) | -1.18 (-6.12, 4.02) |
| **ApoA2** |  |  |  |  |  |  |  |  |
| LCHF vs. A-HCLF | -1.08 (-4.79, 2.77) | 0.38 (-4.63, 5.65) | 3.13 (-2.59, 9.19) | 5.48 (-1.68, 13.2) | 1.61 (-3.01, 6.46) | 3.02 (-3.68, 10.2) | 3.91 (-0.31, 8.30) | 6.47 (-0.10, 13.5) |
| C-HCLF vs. A-HCLF | -0.45 (-5.00, 4.33) | -1.26 (-5.56, 3.23) | 1.49 (-4.92, 8.32) | -1.36 (-7.42, 5.09) | -5.80 (-11.1, -0.21) | -6.99 (-12.2, -1.50) | 1.24 (-3.88, 6.64) | 0.73 (-4.41, 6.15) |
| **ApoA1 in large HDLs** |  |  |  |  |  |  |  |  |
| LCHF vs. A-HCLF | 20.9 (10.1, 32.9) | 32.8 (18.7, 48.5) | 23.2 (9.32, 38.9) | 37.0 (18.6, 58.1) | **13.7 (-3.19, 33.5)** | **38.2 (14.7, 66.5)** | **5.27 (-8.07, 20.5)** | **25.5 (7.11, 47.1)** |
| C-HCLF vs. A-HCLF | 0.26 (-8.23, 9.54) | -4.50 (-12.7, 4.46) | -0.52 (-10.8, 10.9) | -6.85 (-17.1, 4.69) | -9.24 (-22.6, 6.47) | -7.27 (-21.8, 9.93) | -4.18 (-16.3, 9.68) | -6.46 (-18.9, 7.88) |
| **ApoA1 in small HDLs** |  |  |  |  |  |  |  |  |
| LCHF vs. A-HCLF | -0.37 (-4.69, 4.15) | 0.34 (-4.93, 5.91) | 3.74 (-2.38, 10.2) | 5.49 (-2.04, 13.6) | 1.06 (-4.35, 6.77) | 1.62 (-5.30, 9.04) | 6.55 (1.29, 12.1) | 8.66 (1.34, 16.5) |
| C-HCLF vs. A-HCLF | 0.11 (-4.43, 4.86) | -0.16 (-4.77, 4.67) | 3.68 (-2.62, 10.4) | 1.74 (-4.83, 8.75) | -3.33 (-8.52, 2.15) | -3.92 (-9.54, 2.04) | 3.42 (-1.64, 8.74) | 3.17 (-2.69, 9.39) |
| **ApoA2 in large HDLs** |  |  |  |  |  |  |  |  |
| LCHF vs. A-HCLF | **11.8 (-0.43, 25.6)** | **16.1 (0.64, 33.9)** | 8.66 (-6.83, 26.7) | 10.4 (-8.80, 33.7) | 3.61 (-11.9, 21.9) | 10.6 (-9.98, 35.8) | 0.59 (-13.4, 16.8) | 2.98 (-15.3, 25.1) |
| C-HCLF vs. A-HCLF | 6.58 (-5.61, 20.3) | 2.71 (-9.24, 16.2) | -3.63 (-17.6, 12.7) | -11.7 (-25.6, 4.82) | -5.62 (-19.9, 11.2) | -7.01 (-22.3, 11.2) | -5.99 (-18.9, 9.00) | -11.6 (-25.2, 4.48) |
| **ApoA2 in small HDLs** |  |  |  |  |  |  |  |  |
| LCHF vs. A-HCLF | -4.84 (-9.22, -0.24) | -4.65 (-10.5, 1.52) | 0.84 (-6.11, 8.31) | 1.90 (-6.33, 10.9) | 0.58 (-5.90, 7.50) | -1.21 (-8.59, 6.78) | 5.62 (-1.10, 12.8) | 7.47 (-1.10, 16.8) |
| C-HCLF vs. A-HCLF | -0.26 (-5.12, 4.85) | -0.22 (-5.54, 5.41) | 2.45 (-4.76, 10.2) | 1.26 (-6.14, 9.24) | -3.43 (-9.11, 2.60) | -5.09 (-11.1, 1.35) | 5.16 (-1.72, 12.5) | 6.54 (-0.78, 14.4) |
| **ApoB** |  |  |  |  |  |  |  |  |
| LCHF vs. A-HCLF | 1.76 (-4.38, 8.30) | -0.24 (-7.55, 7.65) | 5.37 (-2.37, 13.7) | 5.04 (-4.39, 15.4) | **12.7 (2.42, 24.0)** | **9.16 (-2.62, 22.4)** | 2.35 (-5.69, 11.1) | -1.95 (-12.0, 9.21) |
| C-HCLF vs. A-HCLF | -5.88 (-11.8, 0.43) | -5.62 (-11.6, 0.74) | -1.50 (-8.83, 6.42) | -2.44 (-9.91, 5.66) | 5.86 (-3.90, 16.6) | 2.97 (-6.80, 13.8) | -3.81 (-11.3, 4.27) | -7.14 (-15.2, 1.73) |
| **LDL-C/ApoB** |  |  |  |  |  |  |  |  |
| LCHF vs. A-HCLF | 5.91 (1.79, 10.2) | 8.64 (3.92, 13.6) | 7.84 (2.89, 13.0) | 11.4 (5.30, 17.8) | 10.2 (5.32, 15.3) | 12.9 (6.75, 19.3) | 6.59 (0.86, 12.6) | 15.0 (7.44, 23.0) |
| C-HCLF vs. A-HCLF | -1.45 (-5.44, 2.72) | -1.69 (-5.58, 2.35) | 0.65 (-4.07, 5.61) | 0.19 (-4.79, 5.44) | 0.040 (-4.45, 4.74) | -1.35 (-6.04, 3.57) | 0.39 (-4.92, 5.99) | 2.28 (-3.75, 8.69) |
| **Non-HDL-C/ApoB** |  |  |  |  |  |  |  |  |
| LCHF vs. A-HCLF | 0.75 (-1.24, 2.78) | 1.10 (-1.32, 3.57) | -0.24 (-2.74, 2.33) | -0.46 (-3.41, 2.57) | 0.061 (-2.18, 2.35) | -0.17 (-3.15, 2.90) | 4.12 (1.53, 6.78) | 5.29 (2.05, 8.64) |
| C-HCLF vs. A-HCLF | -0.42 (-2.48, 1.69) | -0.36 (-2.47, 1.80) | -0.52 (-3.07, 2.10) | -1.53 (-4.12, 1.12) | -1.08 (-3.30, 1.20) | -1.64 (-4.09, 0.88) | 2.58 (0.083, 5.14) | 3.01 (0.35, 5.74) |
| **TAG/HDL-C** |  |  |  |  |  |  |  |  |
| LCHF vs. A-HCLF | -30.4 (-40.7, -18.4) | -40.0 (-51.1, -26.5) | -38.2 (-50.3, -23.1) | -44.3 (-57.1, -27.7) | -35.7 (-51.0, -15.8) | -48.0 (-61.6, -29.6) | **-13.8 (-36.7, 17.3)** | **-41.7 (-56.1, -22.7)** |
| C-HCLF vs. A-HCLF | -2.68 (-18.3, 15.9) | 1.02 (-15.9, 21.4) | -7.99 (-25.8, 14.1) | -6.96 (-26.6, 18.0) | 8.39 (-16.6, 40.8) | 9.28 (-17.1, 44.1) | 3.47 (-24.7, 42.2) | -7.45 (-28.0, 19.0) |

**^1^** Relative between-group differences (95% CIs) from baseline for all lipid measures at 3, 6, 9, and 12 months, comparing the primary intention-to-treat analysis with the dietary-adherence adjusted intention-to-treat analysis. Values were natural log-transformed and analysed with constrained linear mixed-effects models adjusted for baseline values, age, sex, and couple-randomisation status, with time and group-by-time interaction included in the models. The A-HCLF intervention was used as the comparator. The diet adherence adjusted models were additionally adjusted for self-reported dietary adherence and deviations between planned and reported intake of energy and macronutrients expressed as energy percentage. Bold values indicate time points where nominal significance status differed between the primary ITT and diet adherence adjusted ITT.

Abbreviations: A-HCLF, acellular high-carbohydrate low-fat; apoA1, apolipoprotein A1; apoB, apolipoprotein B; C-HCLF, cellular high-carbohydrate low-fat; HDL-C, high-density lipoprotein cholesterol; HDLs, high-density lipoprotein particles; IDLs, intermediate-density lipoprotein particles; LCHF, low-carbohydrate high-fat; LDL-C, low-density lipoprotein cholesterol; LDLs, low-density lipoprotein particles; M, months; remnant-C, remnant cholesterol; TAGs, triacylglycerols; TC, total cholesterol; VLDLs, very-low-density lipoprotein particles.

**Supplementary Table 11.** Comparison of primary and inverse probability weighted (IPW) analyses for nominal significance status^1^

|  | **3M** |  | **6M** |  | **9M** |  | **12M** |  | **24M** |  |
| --- | --- | --- | --- | --- | --- | --- | --- | --- | --- | --- |
| **Variable** | **Primary ITT** | **IPW** | **Primary ITT** | **IPW** | **Primary ITT** | **IPW** | **Primary ITT** | **IPW** | **Primary ITT** | **IPW** |
| **TC** |  |  |  |  |  |  |  |  |  |  |
| LCHF vs. A-HCLF | 0,059 | 0,120 | **0,010** | **0,068** | 0,001 | 0,001 | 0,098 | 0,153 | 0,071 | 0,162 |
| C-HCLF vs. A-HCLF | 0,071 | 0,062 | 0,978 | 0,898 | 0,695 | 0,798 | 0,610 | 0,548 | 0,250 | 0,255 |
| **LDL-C** |  |  |  |  |  |  |  |  |  |  |
| LCHF vs. A-HCLF | 0,025 | 0,047 | 0,005 | 0,027 | <0.001 | <0.001 | 0,139 | 0,198 | 0,078 | 0,132 |
| C-HCLF vs. A-HCLF | 0,049 | 0,050 | 0,974 | 0,975 | 0,250 | 0,312 | 0,409 | 0,379 | 0,510 | 0,544 |
| **HDL-C** |  |  |  |  |  |  |  |  |  |  |
| LCHF vs. A-HCLF | 0,000 | 0,001 | <0.001 | <0.001 | <0.001 | 0,001 | 0,009 | 0,030 | 0,415 | 0,612 |
| C-HCLF vs. A-HCLF | 0,859 | 0,686 | 0,427 | 0,393 | 0,071 | 0,074 | 0,762 | 0,788 | 0,132 | 0,243 |
| **Non-HDL-C** |  |  |  |  |  |  |  |  |  |  |
| LCHF vs. A-HCLF | 0,431 | 0,550 | 0,209 | 0,477 | 0,019 | 0,024 | 0,207 | 0,322 | 0,268 | 0,423 |
| C-HCLF vs. A-HCLF | 0,057 | 0,057 | 0,809 | 0,734 | 0,358 | 0,443 | 0,744 | 0,651 | 0,811 | 0,860 |
| **Remnant-C** |  |  |  |  |  |  |  |  |  |  |
| LCHF vs. A-HCLF | 0,001 | 0,003 | 0,002 | 0,002 | 0,003 | 0,004 | 0,078 | 0,121 | **0,041** | **0,068** |
| C-HCLF vs. A-HCLF | 0,939 | 0,827 | 0,934 | 0,797 | 0,854 | 0,761 | 0,563 | 0,505 | **0,049** | **0,344** |
| **TAG** |  |  |  |  |  |  |  |  |  |  |
| LCHF vs. A-HCLF | 0,001 | 0,001 | 0,002 | 0,004 | 0,010 | 0,006 | 0,522 | 0,343 | 0,037 | 0,029 |
| C-HCLF vs. A-HCLF | 0,816 | 0,742 | 0,643 | 0,702 | 0,719 | 0,768 | 0,955 | 0,976 | 0,187 | 0,246 |
| **Total VLDLs** |  |  |  |  |  |  |  |  |  |  |
| LCHF vs. A-HCLF | 0,001 | 0,002 | 0,003 | 0,003 | 0,022 | 0,019 | 0,068 | 0,142 | 0,014 | 0,044 |
| C-HCLF vs. A-HCLF | 0,814 | 0,927 | 0,880 | 0,754 | 0,827 | 0,840 | 0,313 | 0,484 | **0,004** | **0,179** |
| **Total IDLs** |  |  |  |  |  |  |  |  |  |  |
| LCHF vs. A-HCLF | 0,217 | 0,317 | 0,963 | 0,500 | 0,382 | 0,733 | 0,391 | 0,396 | 0,634 | 0,840 |
| C-HCLF vs. A-HCLF | 0,088 | 0,132 | 0,978 | 0,768 | 0,596 | 0,641 | 0,208 | 0,288 | 0,924 | 0,935 |
| **Total LDLs** |  |  |  |  |  |  |  |  |  |  |
| LCHF vs. A-HCLF | 0,142 | 0,212 | **0,041** | **0,125** | 0,002 | 0,002 | 0,354 | 0,543 | 0,095 | 0,151 |
| C-HCLF vs. A-HCLF | 0,143 | 0,136 | 0,858 | 0,824 | 0,220 | 0,275 | 0,445 | 0,384 | 0,662 | 0,707 |
| **Large LDLs** |  |  |  |  |  |  |  |  |  |  |
| LCHF vs. A-HCLF | <0.001 | <0.001 | <0.001 | 0,001 | <0.001 | <0.001 | 0,132 | 0,178 | 0,018 | 0,017 |
| C-HCLF vs. A-HCLF | 0,345 | 0,431 | 0,228 | 0,287 | 0,289 | 0,328 | 0,896 | 0,929 | 0,200 | 0,187 |
| **Medium LDLs** |  |  |  |  |  |  |  |  |  |  |
| LCHF vs. A-HCLF | **0,038** | **0,090** | 0,107 | 0,210 | <0.001 | 0,001 | 0,218 | 0,273 | 0,486 | 0,557 |
| C-HCLF vs. A-HCLF | 0,179 | 0,174 | 0,855 | 0,835 | 0,124 | 0,176 | 0,619 | 0,579 | 0,756 | 0,785 |
| **Small LDLs** |  |  |  |  |  |  |  |  |  |  |
| LCHF vs. A-HCLF | 0,329 | 0,394 | 0,897 | 0,610 | 0,458 | 0,427 | 0,682 | 0,397 | 0,765 | 0,498 |
| C-HCLF vs. A-HCLF | 0,849 | 0,863 | 0,688 | 0,584 | 0,349 | 0,383 | 0,654 | 0,511 | 0,405 | 0,312 |
| **ApoA1** |  |  |  |  |  |  |  |  |  |  |
| LCHF vs. A-HCLF | 0,009 | 0,029 | 0,005 | 0,018 | 0,115 | 0,093 | 0,028 | 0,039 | 0,973 | 0,939 |
| C-HCLF vs. A-HCLF | 0,755 | 0,558 | 0,714 | 0,709 | 0,049 | 0,044 | 0,996 | 0,939 | 0,070 | 0,072 |
| **ApoA2** |  |  |  |  |  |  |  |  |  |  |
| LCHF vs. A-HCLF | 0,577 | 0,580 | 0,289 | 0,600 | 0,499 | 0,577 | 0,070 | 0,308 | 0,098 | 0,198 |
| C-HCLF vs. A-HCLF | 0,851 | 0,784 | 0,656 | 0,574 | 0,042 | 0,038 | 0,640 | 0,595 | 0,123 | 0,082 |
| **ApoA1 in large HDLs** |  |  |  |  |  |  |  |  |  |  |
| LCHF vs. A-HCLF | <0.001 | 0,001 | 0,001 | 0,003 | 0,117 | 0,082 | 0,456 | 0,502 | 0,197 | 0,544 |
| C-HCLF vs. A-HCLF | 0,954 | 0,946 | 0,925 | 0,969 | 0,233 | 0,236 | 0,534 | 0,551 | 0,617 | 0,927 |
| **ApoA1 in small HDLs** |  |  |  |  |  |  |  |  |  |  |
| LCHF vs. A-HCLF | 0,871 | 0,724 | 0,236 | 0,466 | 0,707 | 0,828 | 0,014 | 0,035 | 0,531 | 0,959 |
| C-HCLF vs. A-HCLF | 0,964 | 0,870 | 0,257 | 0,274 | 0,228 | 0,236 | 0,188 | 0,171 | 0,248 | 0,260 |
| **ApoA2 in large HDLs** |  |  |  |  |  |  |  |  |  |  |
| LCHF vs. A-HCLF | 0,059 | 0,110 | 0,289 | 0,537 | 0,667 | 0,597 | 0,939 | 0,903 | 0,779 | 0,783 |
| C-HCLF vs. A-HCLF | 0,303 | 0,250 | 0,641 | 0,626 | 0,488 | 0,461 | 0,412 | 0,411 | 0,138 | 0,157 |
| **ApoA2 in small HDLs** |  |  |  |  |  |  |  |  |  |  |
| LCHF vs. A-HCLF | 0,059 | 0,110 | 0,289 | 0,537 | 0,667 | 0,597 | 0,939 | 0,903 | 0,779 | 0,783 |
| C-HCLF vs. A-HCLF | 0,398 | 0,669 | 0,101 | 0,243 | 0,241 | 0,199 | 0,328 | 0,462 | 0,202 | 0,250 |
| **ApoB** |  |  |  |  |  |  |  |  |  |  |
| LCHF vs. A-HCLF | 0,581 | 0,677 | 0,178 | 0,395 | 0,014 | 0,019 | 0,577 | 0,841 | 0,342 | 0,520 |
| C-HCLF vs. A-HCLF | 0,067 | 0,065 | 0,701 | 0,664 | 0,248 | 0,311 | 0,344 | 0,287 | 0,975 | 0,887 |
| **LDL-C/ApoB** |  |  |  |  |  |  |  |  |  |  |
| LCHF vs. A-HCLF | 0,005 | 0,008 | 0,002 | 0,002 | <0.001 | <0.001 | 0,024 | 0,021 | 0,200 | 0,140 |
| C-HCLF vs. A-HCLF | 0,489 | 0,547 | 0,791 | 0,709 | 0,986 | 0,969 | 0,889 | 0,892 | 0,422 | 0,408 |
| **Non-HDL-C/ApoB** |  |  |  |  |  |  |  |  |  |  |
| LCHF vs. A-HCLF | 0,461 | 0,536 | 0,855 | 0,870 | 0,958 | 0,913 | 0,002 | 0,002 | 0,189 | 0,303 |
| C-HCLF vs. A-HCLF | 0,694 | 0,742 | 0,694 | 0,603 | 0,349 | 0,323 | **0,043** | **0,056** | 0,208 | 0,300 |
| **TAG/HDL-C** |  |  |  |  |  |  |  |  |  |  |
| LCHF vs. A-HCLF | <0.001 | <0.001 | <0.001 | <0.001 | 0,001 | 0,001 | 0,342 | 0,184 | **0,043** | **0,053** |
| C-HCLF vs. A-HCLF | 0,759 | 0,710 | 0,446 | 0,510 | 0,545 | 0,591 | 0,833 | 0,984 | 0,252 | 0,225 |

^1^ Values are nominal p-values for between-group differences in change from baseline from the primary intention-to-treat mixed model and the inverse probability weighted (IPW) sensitivity analysis. The A-HCLF intervention was used as the comparator. Nominal significance was defined as p < 0.05. Bold values indicate time points where nominal significance status differed between the primary intention-to-treat and IPW analyses.

Abbreviations: A-HCLF, acellular high-carbohydrate low-fat; apoA1, apolipoprotein A1; apoB, apolipoprotein B; C-HCLF, cellular high-carbohydrate low-fat; HDL-C, high-density lipoprotein cholesterol; HDLs, high-density lipoprotein particles; IDLs, intermediate-density lipoprotein particles; LCHF, low-carbohydrate high-fat; LDL-C, low-density lipoprotein cholesterol; LDLs, low-density lipoprotein particles; M, months; remnant-C, remnant cholesterol; TAGs, triacylglycerols; TC, total cholesterol; VLDLs, very-low-density lipoprotein particles.

**Supplementary Table 12.** Post hoc exploratory comparison of C-HCLF vs. LCHF for key lipid outcomes in primary ITT and diet adherence adjusted ITT^1^

|  | **Change scores 3M** |  | **Change scores 6M** |  | **Change scores 9M** | | **Change scores 12M** | | **Change scores 24M** | |
| --- | --- | --- | --- | --- | --- | --- | --- | --- | --- | --- |
| **Variable** | **Primary ITT** | **Diet adherence adjusted ITT** | **Primary ITT** | **Diet adherence adjusted ITT** | **Primary ITT** | **Diet adherence adjusted ITT** | **Primary ITT** | **Diet adherence adjusted ITT** | **Primary ITT** |  |
| **TC** |  |  |  |  |  |  |  |  |  |  |
| C-HCLF vs. LCHF | -8.84 (-13.0, -4.50) | -8.43 (-13.9, -2.61) | -7.90 (-13.2, -2.26) | -10.4 (-17.0, -3.34) | -8.98 (-14.4, -3.21) | -10.6 (-17.5, -3.00) | -6.72 (-11.9, -1.20) | -8.30 (-15.3, -0.71) | -2.97 (-11.3, 6.18) |  |
| **LDL-C** |  |  |  |  |  |  |  |  |  |  |
| C-HCLF vs. LCHF | -14.6 (-20.4, -8.45) | -14.8 (-22.1, -6.77) | -12.5 (-19.9, -4.39) | -15.3 (-24.4, -5.17) | -14.2 (-21.8, -5.82) | -17.3 (-25.4, -8.35) | -11.0 (-18.8, -2.60) | -13.8 (-24.2, -1.97) | -7.48 (-19.1, 5.85) |  |
| **HDL-C** |  |  |  |  |  |  |  |  |  |  |
| C-HCLF vs. LCHF | -9.48 (-14.4, -4.27) | -15.3 (-20.7, -9.46) | -12.0 (-17.9, -5.77) | -18.5 (-24.8, -11.6) | -16.9 (-23.0, -10.3) | -24.5 (-31.5, -16.7) | -7.82 (-13.8, -1.37) | -17.3 (-24.3, -9.71) | 4.12 (-6.75, 16.3) |  |
| **Non-HDL-C** |  |  |  |  |  |  |  |  |  |  |
| C-HCLF vs. LCHF | **-8.44 (-13.8, -2.74)** | **-6.58 (-13.8, 1.25)** | -6.12 (-13.1, 1.43) | -7.41 (-16.3, 2.38) | -6.64 (-14.8, 2.26) | -6.58 (-16.7, 4.82) | -6.32 (-13.0, 0.84) | -6.94 (-16.0, 3.11) | -5.93 (-17.7, 7.54) |  |
| **Remnant-C** |  |  |  |  |  |  |  |  |  |  |
| C-HCLF vs. LCHF | 49.8 (16.9, 92.1) | 67.4 (24.5, 125) | 58.2 (16.4, 115) | 62.7 (11.6, 137) | 52.2 (9.80, 111) | 84.8 (23.5, 177) | **19.0 (-12.5, 61.8)** | **56.3 (7.21, 128)** | 0.92 (-30.3, 46.1) |  |
| **TAG** |  |  |  |  |  |  |  |  |  |  |
| C-HCLF vs. LCHF | 25.3 (6.73, 47.0) | 36.3 (13.9, 63.1) | 29.4 (7.80, 55.3) | 35.8 (10.7, 66.5) | 41.9 (13.5, 77.5) | 62.2 (25.0, 110) | **10.8 (-10.1, 36.6)** | **29.3 (1.08, 65.4)** | 10.8 (-16.7, 47.3) |  |
| **Total VLDLs** |  |  |  |  |  |  |  |  |  |  |
| C-HCLF vs. LCHF | 30.6 (11.5, 53.0) | 43.0 (18.5, 72.5) | 29.8 (7.30, 56.9) | 37.4 (7.95, 74.9) | 31.8 (4.88, 65.6) | 53.2 (15.6, 103) | **8.91 (-11.8, 34.5)** | **35.9 (6.04, 74.1)** | -3.71 (-25.2, 23.9) |  |
| **Total IDLs** |  |  |  |  |  |  |  |  |  |  |
| C-HCLF vs. LCHF | -3.17 (-13.3, 8.14) | -8.66 (-20.2, 4.60) | 0.14 (-13.1, 15.4) | -9.81 (-24.6, 7.80) | -3.27 (-19.6, 16.4) | -16.2 (-30.9, 1.73) | -2.80 (-15.1, 11.3) | -9.17 (-22.7, 6.67) | -4.78 (-25.5, 21.7) |  |
| **Total LDLs** |  |  |  |  |  |  |  |  |  |  |
| C-HCLF vs. LCHF | **-10.3 (-16.2, -3.89)** | **-8.43 (-16.2, 0.030)** | **-9.28 (-16.3, -1.71)** | **-9.46 (-18.7, 0.84)** | **-9.05 (-17.2, -0.089)** | **-8.21 (-17.2, 1.81)** | -7.73 (-15.3, 0.50) | -5.75 (-16.3, 6.18) | -7.96 (-19.1, 4.67) |  |
| **Large LDLs** |  |  |  |  |  |  |  |  |  |  |
| C-HCLF vs. LCHF | -18.5 (-24.5, -12.1) | -19.4 (-27.2, -10.7) | -13.4 (-21.1, -4.93) | -18.3 (-27.9, -7.40) | -19.0 (-26.4, -10.9) | -21.8 (-31.0, -11.3) | **-11.2 (-21.9, 1.06)** | **-18.0 (-30.8, -2.69)** | -7.78 (-20.2, 6.56) |  |
| **Medium LDLs** |  |  |  |  |  |  |  |  |  |  |
| C-HCLF vs. LCHF | -16.9 (-25.0, -8.06) | -19.2 (-29.3, -7.72) | -11.7 (-22.3, 0.25) | -18.1 (-30.9, -2.80) | -13.8 (-25.0, -1.01) | -18.6 (-32.1, -2.45) | -13.4 (-25.3, 0.43) | -21.0 (-35.9, -2.74) | -4.47 (-23.9, 19.9) |  |
| **Small LDLs** |  |  |  |  |  |  |  |  |  |  |
| C-HCLF vs. LCHF | 5.23 (-7.03, 19.1) | 10.0 (-5.95, 28.7) | -1.75 (-12.5, 10.4) | 4.15 (-12.2, 23.5) | 1.87 (-13.9, 20.6) | 3.68 (-16.5, 28.8) | -0.24 (-12.1, 13.3) | 8.11 (-9.78, 29.5) | -6.23 (-25.2, 17.5) |  |
| **ApoA1** |  |  |  |  |  |  |  |  |  |  |
| C-HCLF vs. LCHF | -5.46 (-8.79, -2.01) | -8.93 (-13.1, -4.53) | -6.03 (-10.3, -1.53) | -11.9 (-17.2, -6.24) | -8.71 (-13.1, -4.13) | -13.1 (-19.0, -6.84) | -5.23 (-9.26, -1.03) | -10.8 (-16.4, -4.83) | 7.18 (-0.36, 15.3) |  |
| **ApoA2** |  |  |  |  |  |  |  |  |  |  |
| C-HCLF vs. LCHF | 0.64 (-3.61, 5.08) | -1.64 (-6.76, 3.76) | -1.59 (-7.25, 4.41) | -6.49 (-12.9, 0.39) | -7.30 (-12.2, -2.12) | -9.72 (-15.8, -3.19) | -2.56 (-7.06, 2.15) | -5.39 (-11.2, 0.82) | 0.75 (-6.36, 8.41) |  |
| **ApoA1 in large HDL** | |  |  |  |  |  |  |  |  |  |
| C-HCLF vs. LCHF | -17.1 (-24.9, -8.53) | -28.1 (-36.6, -18.4) | -19.3 (-28.3, -9.06) | -32.0 (-41.6, -20.7) | -20.2 (-30.7, -8.02) | -32.9 (-43.3, -20.6) | **-8.98 (-19.6, 3.00)** | **-25.5 (-37.4, -11.3)** | 16.1 (-12.8, 54.5) |  |
| **ApoA1 in small HDL** | |  |  |  |  |  |  |  |  |  |
| C-HCLF vs. LCHF | 0.47 (-3.73, 4.86) | -0.50 (-5.99, 5.30) | -0.055 (-5.60, 5.81) | -3.56 (-10.5, 3.93) | -4.34 (-9.28, 0.86) | -5.45 (-12.1, 1.73) | -2.94 (-7.31, 1.65) | -5.05 (-11.4, 1.78) | 1.73 (-4.15, 7.96) |  |
| **ApoA2 in large HDL** | |  |  |  |  |  |  |  |  |  |
| C-HCLF vs. LCHF | -4.71 (-14.8, 6.60) | -11.5 (-23.8, 2.69) | **-11.3 (-23.2, 2.40)** | **-20.0 (-34.2, -2.78)** | -8.91 (-22.1, 6.52) | -15.9 (-31.9, 3.85) | -6.54 (-18.4, 7.07) | -14.1 (-29.2, 4.06) | 16.4 (-7.88, 47.2) |  |
| **ApoA2 in small HDL** | |  |  |  |  |  |  |  |  |  |
| C-HCLF vs. LCHF | 4.81 (-0.64, 10.6) | 4.65 (-2.00, 11.8) | 1.59 (-4.76, 8.37) | -0.63 (-8.78, 8.24) | -3.99 (-10.6, 3.13) | -3.94 (-11.4, 4.17) | -0.43 (-5.91, 5.37) | -0.87 (-8.76, 7.70) | -2.20 (-12.7, 9.54) |  |
| **ApoB** |  |  |  |  |  |  |  |  |  |  |
| C-HCLF vs. LCHF | **-7.51 (-12.9, -1.78)** | **-5.39 (-12.7, 2.55)** | -6.52 (-13.0, 0.39) | -7.12 (-15.8, 2.45) | -6.07 (-14.3, 2.99) | -5.67 (-16.1, 6.08) | -6.02 (-12.8, 1.25) | -5.29 (-14.9, 5.39) | -6.35 (-17.5, 6.33) |  |
| **LDL-C/ApoB** |  |  |  |  |  |  |  |  |  |  |
| C-HCLF vs. LCHF | -6.95 (-10.4, -3.33) | -9.51 (-13.6, -5.25) | -6.67 (-10.7, -2.46) | -10.0 (-15.0, -4.76) | -9.22 (-13.1, -5.18) | -12.6 (-17.5, -7.43) | -5.82 (-10.4, -1.03) | -11.0 (-16.6, -5.11) | -1.27 (-6.26, 3.98) |  |
| **Non-HDL-C/ApoB** | |  |  |  |  |  |  |  |  |  |
| C-HCLF vs. LCHF | -1.16 (-3.06, 0.77) | -1.44 (-3.88, 1.07) | -0.28 (-2.62, 2.11) | -1.07 (-4.04, 1.99) | -1.14 (-3.30, 1.07) | -1.48 (-4.52, 1.66) | -1.48 (-3.67, 0.76) | -2.16 (-5.07, 0.83) | -0.073 (-2.59, 2.51) |  |
| **TAG/HDL-C** |  |  |  |  |  |  |  |  |  |  |
| C-HCLF vs. LCHF | 39.9 (16.6, 67.8) | 68.5 (35.0, 110) | 48.8 (21.2, 82.7) | 67.1 (26.4, 121) | 68.6 (29.8, 119) | 110 (52.1, 190) | **20.1 (-6.41, 54.1)** | **58.7 (17.6, 114)** | 6.92 (-25.6, 53.7) |  |

^1^ Values are relative between-group differences from baseline with 95% CIs for key lipid outcomes at 3, 6, 9, 12, and 24 months, comparing LCHF with C-HCLF in the primary ITT analysis and the diet-adherence adjusted ITT analysis (up to 12-months). Values were natural log-transformed and analysed with constrained linear mixed-effects models adjusted for baseline values, age, sex, and couple-randomisation status, with time and group-by-time interaction included in the models. The diet-adherence adjusted models were additionally adjusted for self-reported dietary adherence and deviations between planned and reported intake of energy and macronutrients expressed as E%. These analyses were conducted post hoc and should be interpreted as exploratory, as they were not part of the prespecified pairwise comparisons. Bold values indicate time points where nominal significance status differed between the primary ITT and diet adherence adjusted ITT.

Abbreviations: A-HCLF, acellular high-carbohydrate low-fat; apoA1, apolipoprotein A1; apoB, apolipoprotein B; C-HCLF, cellular high-carbohydrate low-fat; HDL-C, high-density lipoprotein cholesterol; HDLs, high-density lipoprotein particles; IDLs, intermediate-density lipoprotein particles; LCHF, low-carbohydrate high-fat; LDL-C, low-density lipoprotein cholesterol; LDLs, low-density lipoprotein particles; M, months; Remnant-C, remnant cholesterol; TAGs, triacylglycerols; TC, total cholesterol; VLDLs, very-low-density lipoprotein particles.
